# Reductive Dimerization of Macrocycles Activated by BBr_3_

**DOI:** 10.1021/acs.orglett.1c01047

**Published:** 2021-04-15

**Authors:** Monika Kijewska, Miłosz Siczek, Miłosz Pawlicki

**Affiliations:** † Department of Chemistry, University of Wrocław, F. Joliot-Curie 14, 50383 Wrocław, Poland; ‡ Faculty of Chemistry, Jagiellonian University, Gronostajowa 2, 30387 Kraków, Poland

## Abstract

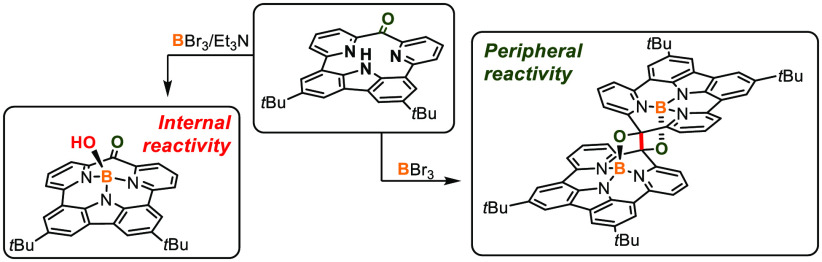

A macrocyclic motif composed of carbazole and pyridine subunits linked by a carbonyl bridge (C=O) forms a skeleton with a peripheral reactivity that leads to a pinacol-like coupling activated by BBr_3_, eventually entrapping a substantially elongated C–C bond. Slightly modified conditions lead to the efficient transformation of the C=O unit to a CH_2_ linker that, after exposure to air, gives a dimeric molecule with multiple bonds between two macrocyclic units, as documented in spectroscopy and X-ray analysis.

The precise design of novel structural motifs with envisioned properties remains the central theme of logic in chemistry. Such structures, apart from potential applications, often provide platforms for the exploration and development of novel concepts in fundamental aspects such chemical bonding or potential reactivity. The formation of unsaturated macrocycles that keep local conjugation opens a specific set of options for postsynthetic transformation^1^ in addition to opening new frontiers of reactivity. Pyridine-incorporated structures are reported as motifs where both types of behavior have been observed to show global conjugation^2^ or to keep local effects^3^ and postsynthetic modifications.^4^ The coordination plays an important role in macrocycles, strongly modulating the observed behavior, including the formation of dimeric motifs driven by coordination^5^ that can lead to μ-*oxo*-dimers.^6^ The linking of two monomeric macrocycles into covalently bound skeletons was realized via a transition-metal catalyst^7^ or with a standard condensation approach.^8^ As reported to date, the triheterocyclic/carbocyclic systems linked with carbon bridges form a perfect environment for binding boron(III), which effectively interacts with carbon^9^ or nitrogen^10^ but also stabilizes a global effect of antiaromatic delocalization.^11^ However, the triangular shape of the macrocyclic environment that is predefined for binding boron(III) can create different sets of donors very efficiently, utilizing the electron deficiency of the central ion and leading to the intriguing modulation of the final skeleton, while the global delocalization is disturbed and the local influences are crucial.^1,4^ Following these observations, here we report the studies of the precisely designed and synthesized macrocycle constructed of heterocyclic subunits linked with a carbonyl (C=O) unit, showing two reactivities distinguished as *internal* and *peripheral*.

In our approach, we applied Suzuki–Miyaura coupling as a versatile tool for the formation of π-extended molecules,^9−12^ and it is also applicable for the synthesis of macrocycles.^13^ Both required reagents **1** and **2** were obtained as previously described^14,15^ and subjected to palladium-catalyzed coupling (Scheme 1, path *a*), eventually giving macrocycle **3** in 20% yield. As documented in the ^1^H NMR analysis (Figure 1A), **3** remains locally aromatic according to the magnetic criterion,^18^ with a downfield-shifted line of H(3,20) at δ 8.18. The recorded spectroscopic properties are consistent with rather negligible macrocyclic delocalization compared with other structures of similar size^9−12^ but also containing pyridine and carbazole.^16^ The lack of macrocyclic delocalization is consistent with the presence of the carbonyl linker documented in the ^13^C NMR (δ 187.1) that efficiently blocks the delocalization. The final structure shows a strongly downfield-shifted internal proton of the NH group (δ 18.48), proving the proximity of three nitrogen centers and a strong hydrogen bond responsible for the significant deshielding of hydrogen.^4,17^ The presence of three nitrogen donors in a confined macrocyclic construction similar to triphyrins creates a perfect environment for small cations such as boron(III).^10,11^ The reaction of **3** with boron(III) tribromide (BBr_3_) in the presence of triethyl amine (Et_3_N), applied as a neutralizing medium, showed the *internal* variant of reactivity (Scheme 1, path *b*). The LC-MS analysis of the reaction mixture showed an *m*/*z* peak at 486.2330, consistent with that of monomeric skeleton **4a** with a hydroxy axial ligand isolated in 28% yield. A deeper analysis of the reaction mixture showed the presence of doubly charged motif at *m*/*z* 477.2288 (+2), eventually assigned to μ-*oxo*-dimer **5** (Scheme 1). The formation of oxygen-bridged structures was reported for different cations including transition metals^6a^ and metalloids.^6b^ As documented, the formation of **5** was observed in the presence of Et_3_N, which activates the axial hydroxy ligand at the boron(III) center. Importantly, **5** can be also directly obtained from **4a** (Scheme 1, path *e*) in 45% yield. The insertion of B(III) does not change the local character of aromaticity observed in **4a,** as documented by ^1^H NMR (Figure 1B). The presence of a carbonyl unit in both derivatives **4a** and **5** was proved by the ^13^C chemical shifts observed at δ 177.0 and δ 176.1, respectively. **4** showed a specific pattern of pyridine resonances with a significantly downfield-shifted line of H(3,20) (δ 9.54 (**4a**)), whereas **5** relocated the pyridine resonances H(3,20) up-field to δ 9.16 (Figure S39).

**Scheme 1:**
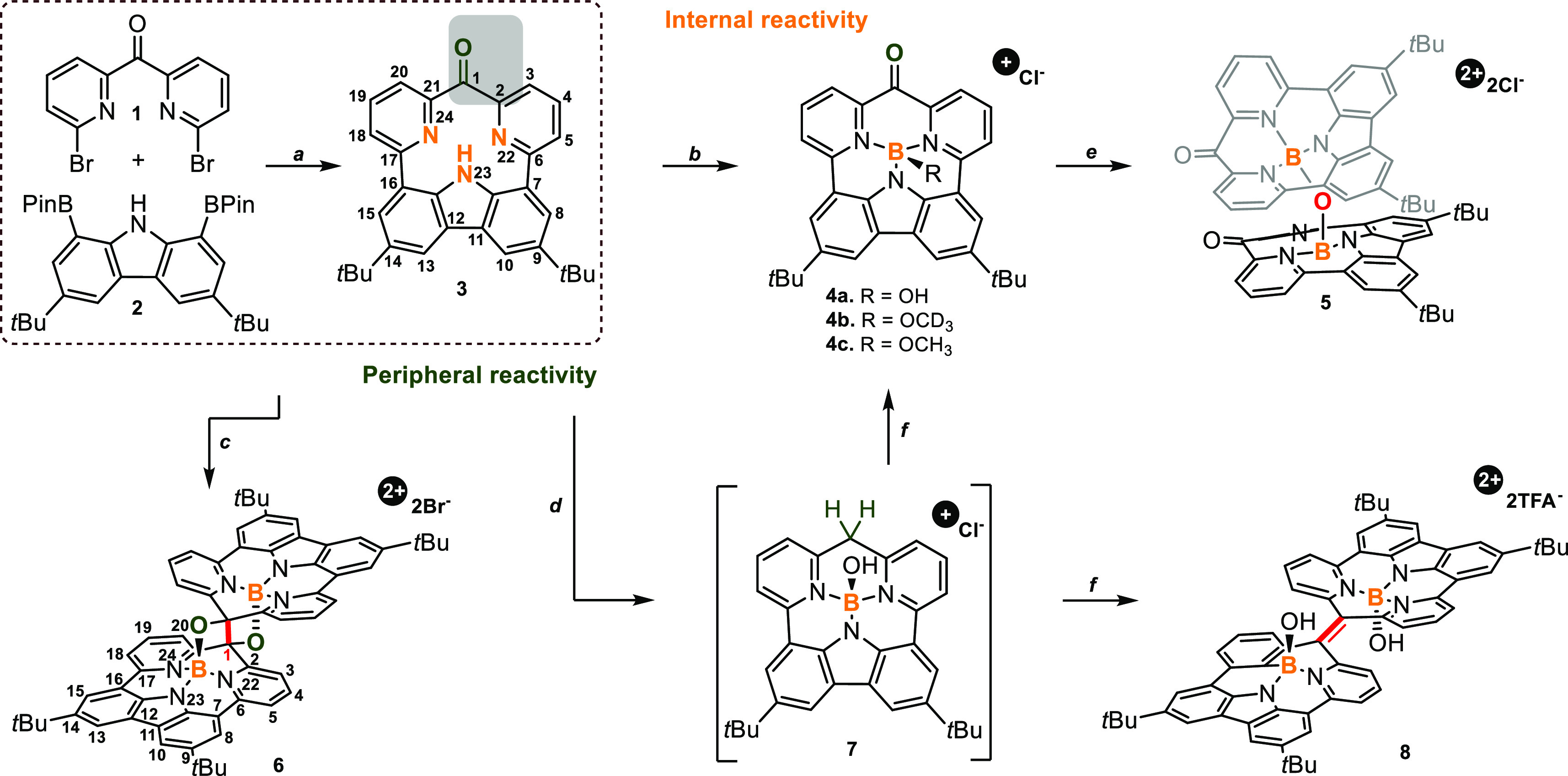
Synthetic Approach Conditions: (a) **1** (1 equiv), **2** (1 equiv), Pd(PPh_3_)_4_ (0.1 equiv), K_2_CO_3_ (3 equiv), KF (4 equiv), toluene/DMF, 110 °C, 72 h. (b) BBr_3_ (10 equiv), Et_3_N (13 equiv), toluene, reflux, Ar, 2 h. (c) BBr_3_ (10 equiv), toluene, reflux, Ar, 2 h. (d) BBr_3_ (10 equiv), *o*-dichlorobenzene, reflux, Ar, 2 h. (e) Zn(Hg), CDCl_3_, Ar, 24 h (incubation 12 h, 60°C). (f) O_2_, 24 h.

**Figure 1:**
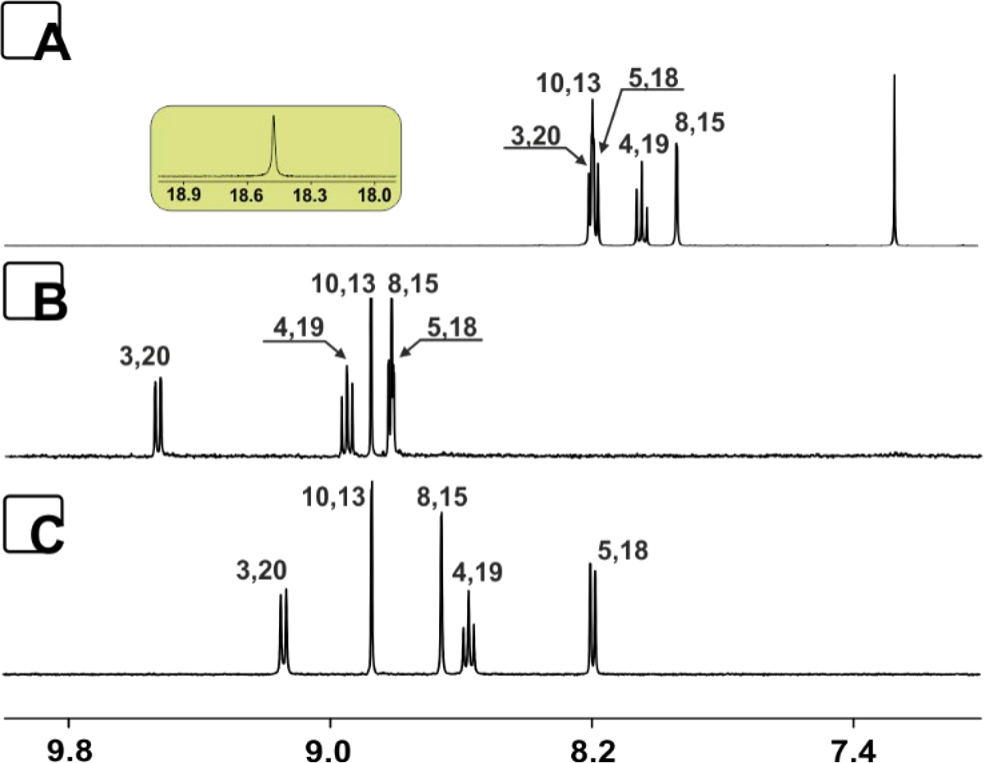
^1^H NMR spectra of **3** (A, chloroform-*d*), **4a** (B, acetone-*d*_6_), and **6** (C, acetone-*d*_6_) (600 MHz, 300 K).

While dissolved in acetone-*d*_6_, both positively charged boron(III) complexes **4** and **5** efficiently accommodate one molecule of acetone via an enolation of acetone that acts as a nucleophile toward the carbonyl unit, forming an sp^3^-hybridized carbon and showing peripheral reactivity. The ^13^C NMR spectra showed the formation of a tetrahedral carbon for the conversion of **4a** (δ 75.9 **4**-*Ac*), whereas the reaction of **5** gave two types of carbons (δ 176.1 and δ 75.4), proving the accommodation of one molecule of acetone in **5**-*Ac* (Figure 2). The significant difference in both positions (Figure S46) is consistent with keeping the sp^2^ carbonyl in one subunit and changing the hybridization to sp^3^ in the second one.

**Figure 2:**
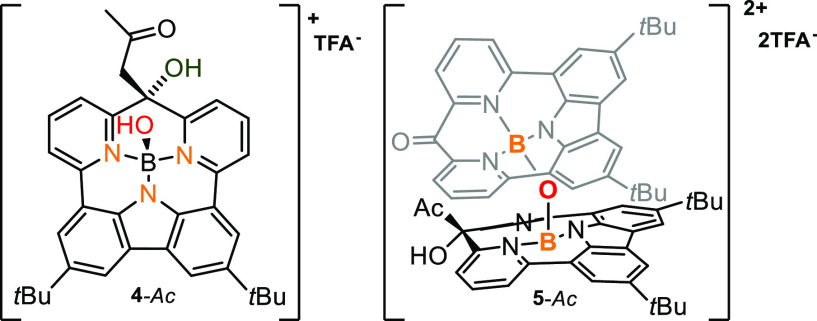
Acetone adducts of **4-***Ac* and **5-***Ac*.

The X-ray analysis performed for **3** confirmed steric confinements resulting in the proximity of three nitrogen donors (Figure 3A), and the distances observed within the coordination cavity are consistent with a strong N–H···N hydrogen bond responsible for the substantial downfield shift of H(23) in the ^1^H NMR spectrum.^4,17^ Crystal structures confirmed the presence of carbonyl units in **3** and **4a** (Figure 3A,B; C(1)–O(1) bond lengths 1.223(3) and 1.217(6) Å, respectively) and the tetrahedral geometry of C1 in **4a**-*Ac* (Figure 3C). The boron(III) environment in **4a** and **4a**-*Ac* showed the pyramidal organization of donors with characteristic bond lengths (Figure 3) and a hydroxyl group as an axial ligand. The crystal structure of **5**-*Ac* (see the Supporting Information) showed the presence of both types of subunits, confirming the formation of another C–C bond (Figure S105).

**Figure 3:**
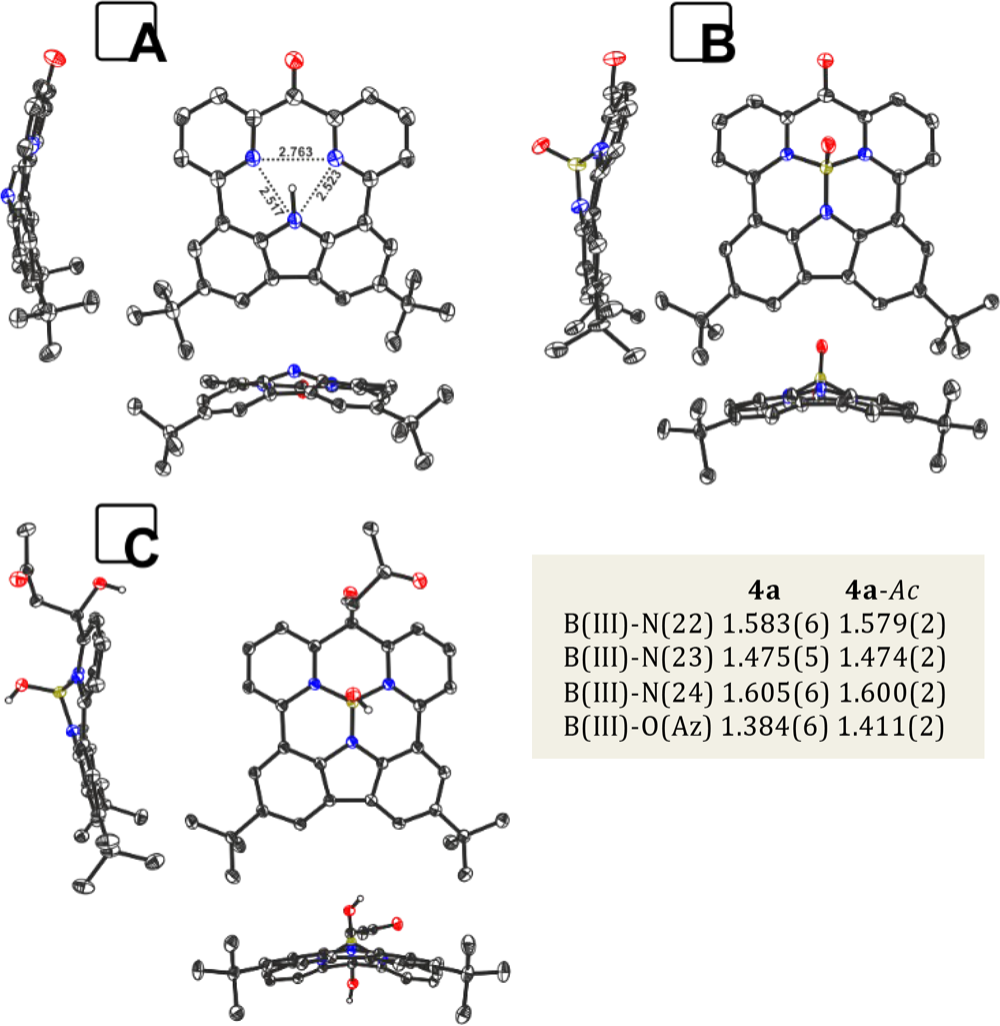
Molecular structures of **3** (A, thermal ellipsoids present 30% probability), **4a** (B, thermal ellipsoids present 50% probability), and **4-***Ac* (C, thermal ellipsoids present 50% probability). Counter ions, solvents, and hydrogen atoms are removed for clarity.

The reactivity at the carbonyl unit observed in **4** and **5** consistently suggested the potential toward the formation of new carbon–carbon bonds, opening a path for the *peripheral* reactivity (Scheme 1). Carbonyl units are effective substrates for reactions like pinacol coupling, which consists of a reduction step crucial for the formation of a C–C bond and requiring the employment of transition metals.^7,20^ The reaction of **3** with boron(III) tribromide (BBr_3_, *excess*) without triethyl amine (Scheme 1, path *c*) gave a product that showed a doubly charged peak at *m*/*z* = 469.2311 as a major component accompanied by a substantially smaller amount of **4a** and a second doubly charged structure with an *m*/*z* peak at 470.2381 (Figure S80).

The isotopic patterns recorded for both doubly charged fractions were consistent with the presence of two boron(III) cations. The NMR analysis with substantially different patterns of resonances in the ^1^H spectrum compared with **4a** with noticeably upfield-relocated pyridine lines (Figure 1C) and the presence of an sp^3^ carbon (^13^C δ 84.5, Figure S56) connected to a heteroatom allowed us to suggest the expected structure for the major fraction to be **6** (Scheme 1) stabilizing a pinacol motif. The second dimeric structure, eventually identified as **8** (Scheme 1), showed a substantially different spectroscopic pattern consistent with a double bond linking two macrocyclic subunits. The X-ray analysis of **6** (Figure 4A) confirmed the conclusions derived from spectroscopic data. An sp^3^-hybridized carbon in a pinacol-like motif that coordinates to boron(III) centers (Figure 3A) was observed. The boron(III) environment (B–N(22) (1.573(6) Å), B–N(23) (1.432(6) Å) and B–N(24) 1.569(6) Å) was comparable to that for **4a** and also that for other B(III)–N interactions. Both macrocyclic subunits are linked via two B–O–C connections that keep two sp^3^-hybridized C(1) atoms in an orientation that forms a C–C bond with a length of 1.659(6) Å, which is noticeably longer than regular sp^3^–sp^3^ interaction.^19^ The Wiberg index calculated for the elongated C–C bond (0.8416) confirmed the decreased bond order, consistent with the observed length. The crystal structure of **8** (Figure 4B) confirmed the presence of a significantly shorter C–C bond (1.354(4) and 1.344(4) Å for two independent molecules in a crystal cell) characteristic of a double interaction. Both dimeric structures were stable and could be kept in solution for several weeks while protected from the air. Unprotected from the air, the sample of **6** in methanol(methanol-*d*_4_) or the mixture of acetonitrile/water quantitatively converted to **4b**(**4c**) or **4a**, respectively (Figures S89 and S90), which can explain the presence of **4a** in the reaction mixture. Under the same conditions, **8** remained intact.

**Figure 4:**
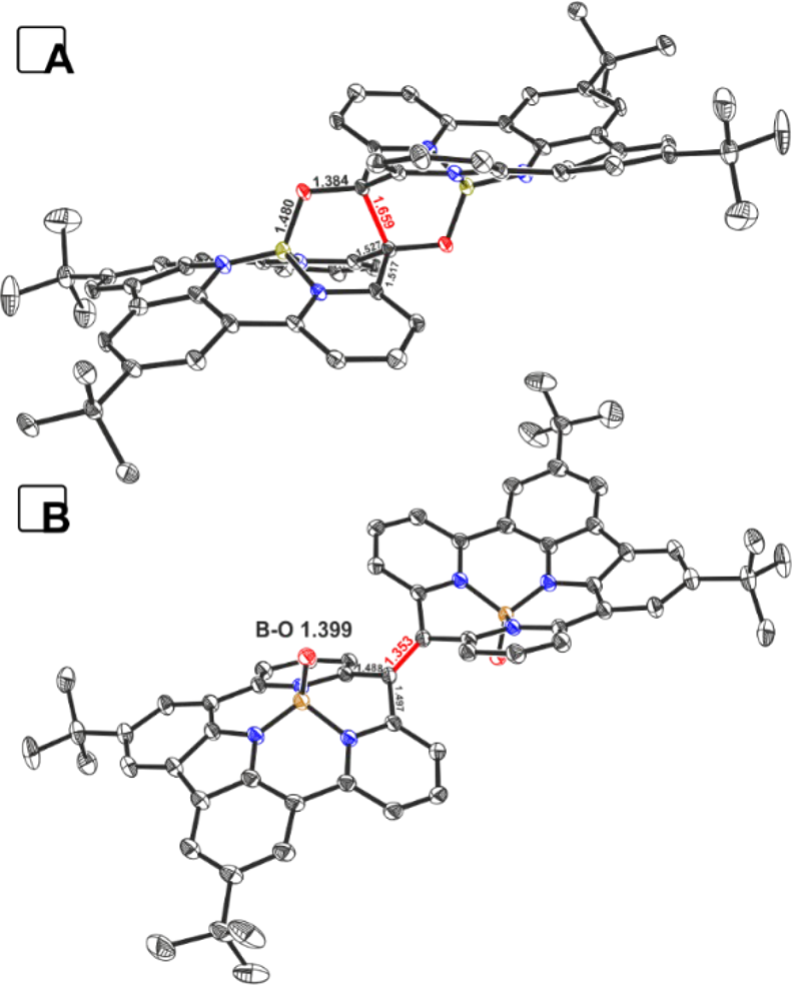
Crystal structures of **6** (A) and **8** (B) (thermal ellipsoids at 30% probability). Counter ions, solvents, and hydrogen atoms are removed for clarity.

The UV–vis properties consistently support the limited global delocalization, showing a negligible change when comparing all derivatives (Figure S99). The NH dynamic substantially affects the emission^21^ that is not observed for **3** but is detectable for boron(III) complexes (Figure S103) and recorded at λ = 506 (**4a**), 510 (**6**), and 511 nm (**8**).

The plausible mechanism leading to **6** involves the dimerization of monomers **3** activated by BBr_3_, leading to a tetracationic skeleton (Scheme 2), followed by a reductive C–C bond formation. The applied conditions do not introduce any obvious source of electrons; however, Br^–^ was reported as an electron-transfer medium acting as a reducing agent in multicharged skeletons.^23a^ Thus an excess of bromide anions plays a crucial role in dimerization, and the observed behavior shows the influence of the introduction of positive charges to the system followed by the electron transfer that reflects some analogy to the proton-coupled electron transfer (PCET).^23^

**Scheme 2:**
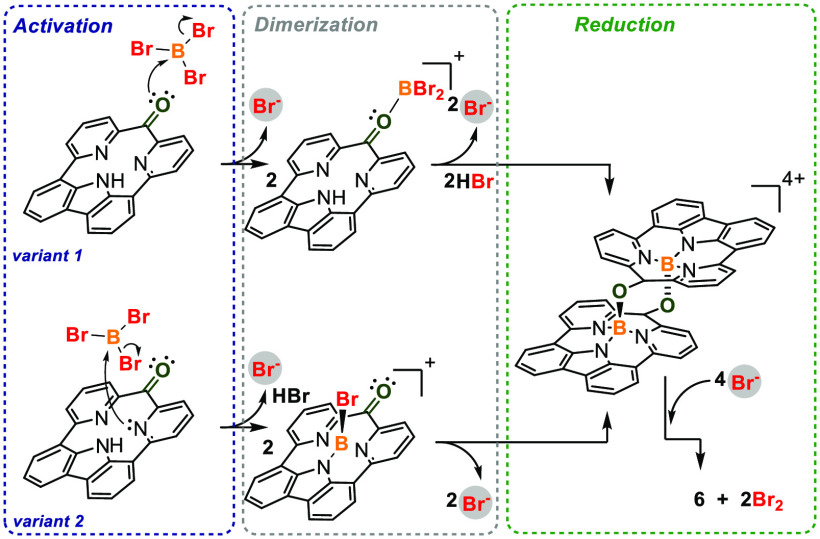
Potential Mechanism in Two Variants for the Reductive Coupling of Carbonyls Activated with BBr_3_ *t*Bu is not present for clarity.

While looking at **6** and **8**, it can be concluded that both dimeric structures can be interconverted by a redox process. Nevertheless, all reduction attempts to convert **6** to **8** were met with failure, suggesting a separate path. Because we did not observe the formation of **8** under the first conditions (with Et_3_N), we decided to modify the synthetic approach leading to **6** by extending the reaction time to 12 h of reflux in toluene. This gave **6** as a dominating component, accompanied by **4a** and **8**. The LC-MS analysis showed an additional monocationic compound at an *m*/*z* signal of 472.2553 that vanishes after exposure to air with a simultaneous increase in the amount of **4a** and **8** (Figure S95), suggesting a correlation between these species. By using a highly boiling solvent (*o*-dichlorobenzene) for the same process (Scheme 1, path *d*), we observed the formation of 472.2553 as a single product that was eventually assigned to the reduced skeleton **7** (Scheme 1) containing a −CH_2_– bridge instead of a carbonyl unit (Figures S96 and S97). As reported by Newkome and coworkers, a −CH_2_– group flanked by two pyridines is highly reactive and leads to dimerization,^16d,22^ similar to our observations where **7** converts to **8** (Scheme 1, path *f*). A potential approach to reduce the carbonyl unit to form **7** can be presented with similarities to the classic Clemmensen reduction (Scheme S2) with the involvement of Br^–^ as an electron-transfer agent. Both reductive processes leading to **6** and **7** should have similar origins, as both derivatives were observed under similar conditions. Thus while looking at the optimized conditions, we can say that depending on the presence of triethyl amine, the predominant formation of monomeric (with Et_3_N) or dimeric (without Et_3_N) boron(III) complexes can be observed. In addition, the different reaction temperatures can be applied to form either **6** or **8** (via **7**).

In conclusion, a rationally obtained macrocycle armed with peripherally located carbonyl functionality undergoes a BBr_3_-activated conversion that, depending on the applied conditions, gives monomeric or dimeric boron(III) complexes. The dimerization gives a pinacol-like coupling of two subunits with the stabilization of the elongated C–C bond, as documented in the X-ray analysis. With slightly modified conditions, two macrocycles form a double bond that bridges both subunits. The presented approach, with the possibility of controlling the type of reactivity, shows that the precise design of the skeleton opens the potential for controlled conversion to highly appreciated motifs linking π-extended systems into more complex structures. Further experiments toward this reactivity are under way in our lab.

## Supporting Information Available

General methods, synthesis, and characterization of all compounds (PDF)

## Accession Codes

CCDC 2054315–2054319 contain the supplementary crystallographic data for this paper. These data can be obtained free of charge via www.ccdc.cam.ac.uk/data_request/cif, or by emailing data_request@ccdc.cam.ac.uk, or by contacting The Cambridge Crystallographic Data Centre, 12 Union Road, Cambridge CB2 1EZ, UK; fax: +44 1223 336033.
