# Supplementary material for: Reductive Dimerization of Macrocycles Activated by BBr3
Source: Org Lett. 2021 Apr 15;23(9):3652–6. doi: 10.1021/acs.orglett.1c01047 (PMC8155568; doi:10.1021/acs.orglett.1c01047)
Supplement: Supplementary file 1 — ol1c01047_si_001.pdf [file ol1c01047_si_001.pdf]

Table of contenst

1. General information 2
2. Experimental section 3
2.1 Experimental procedures 3
3. NMR spectra 7
3.1 NMR spectra of 2 7
3.2 NMR spectra of 3 10
3.3 NMR spectra of 4a 15
3.4 NMR spectra of 4b 21
3.5 NMR spectra of 4-Ac 26
3.6 NMR spectra of 5 and 5-Ac 28
3.7 NMR spectra of 6 34
3.8 NMR spectra of 8 39
3.9 NMR spectra of incubation of 6 in MeOD 40
4. LC-MS and MS spectra 42
4.1 ESI-MS spectrum of 2 42
4.2 ESI-MS spectrum of 3 43
4.3 ESI-MS spectrum of 4a 44
4.4 ESI-MS spectra of 4b 45
4.5 ESI-MS spectra of 4c 46
4.6 ESI-MS spectrum of 4-Ac 47
4.7 ESI-MS spectrum of 5 47
4.8 ESI-MS spectrum of 5-Ac 48
4.9 ESI-MS spectrum of 6 49
4.10 ESI-MS spectrum of 8 50
4.11 LC-MS of crude mixture (Procedure 1) 51
4.12 LC-MS of crude mixture (Procedure 2) 52
4.13 LC-MS of 4a 53
4.14 LC-MS of 4b 53
4.15 LC-MS of 4c 54
4.16 LC-MS of 5 54
4.17 LC-MS of 6 55
4.18 LC-MS of 8 55
4.19 LC-MS of incubation of 6 in water-acetonitrile (v:v, 1:1) in 60°C for 12 h 56
4.20 LC-MS of incubation of 4a in water-acetonitrile (v:v, 1:1) in 60°C for 12h 57
4.21 LC-MS of incubation of 6 in CD_3OD in 60°C for 12 h 58
4.22 LC-MS of incubation of 6 in MeOH in 60°C for 2 h 59
4.23 LC-MS of incubation of mixture of 4a and 6 in CD_3OD in 60°C for 2 h 60
4.24 LC-MS of incubation of pure 5 in acetone-d6 61
4.25 LC-MS of products after reduction of 4a 62
4.26 LC-MS of products after incubation of 4a and 4b 63
4.27 LC-MS of crude products (Procedure 2a) 64
4.28 LC-MS of products before and after purification (Procedure 3) 65
5. UV-Vis spectra 66
6. Emission spectra 68
7. Crystal Data 70

1. General information

NMR Spectroscopy. ^{1}H NMR spectra were recorded on a high-field spectrometer (^{1}H 600.15 MHz and 500 MHz, ^{13}C 150 MHz and 125.75 MHz), equipped with a broadband inverse gradient probehead. Spectra were referenced to the residual solvent signal (chloroform-d, 7.24 ppm or acetone-d6 2.05 ppm). Two dimensional NMR spectra were recorded with 2048 data points in the t2 domain and up to 1024 points in the t1 domain, with a 1s recovery delay.

Mass Spectrometry. High resolution and Accurate Mass spectra were recorded on a Bruker apex ultra Apex-Qe 7T instrument (Bruker) spectrometer using the electrospray technique. The acetonitrile, dichloromethane or methanol were used as solvents for recording the mass spectra. The potential between the spray needle and the orifice was set to 4.5 kV.

LC-MS analysis. The LC-MS analysis was performed on Shimadzu LC IT-TOF. Separation was carried out on an RP-Zorbax (50×2.1 mm, 3.5 µm) column with a gradient elution of 0-80% B in A (A = 0.1% HCOOH in water; B = 0.1% HCOOH in MeCN) at room temperature over a period of 20 min (flow rate: 0.1 mL/min).

UV-Vis Spectroscopy. Electronic spectra were recorded on a Varian Carry-50 Bio spectrophotometer.

Fluorescence. Steady state fluorescence spectra were recorded with a JASCO FP-8600 Spectrofluorometer apparatus.

X-Ray Analysis. X-Ray quality crystals were prepared by precipitation from DCM/MeOH (**3**), $CDCl_3$ (**4a**, **6**) and $(CD_3)_2CO$ (**5-*Ac***, **4-*Ac***, **8**). Diffraction data were collected on a Rigaku Oxford Diffraction XtaLAB Synergy-R DW diffractometer equipped with a HyPix ARC 150° Hybrid Photon Counting (HPC) detector using CuK$_\alpha$ (λ = 1.54184 Å) for **4-*Ac*, 5-*Ac*** and **8** at **80**, **100** and **100 K**, respectively**.** Diffraction data for **3** were collected on a Kuma KM4 diffractometer equipped with Sapphire CCD detector at 100 K using MoK$_\alpha$ (λ = 0.71073 Å). Diffraction data for **4a** and **6** were collected on a Xcalibur Gemini Ultra diffractometer equipped with Ruby CCD detector at 100 K using CuK$_\alpha$ (λ = 1.54184 Å). Data were processed using the CrystAlisPro software. The structures were solved by intrinsic phasing with SHELXT (2015 release) and refined by full-matrix least-squares methods based F^2 using SHELXL. For all structures, H atoms bound to C atoms were placed in the geometrically idealized positions and treated in riding mode, with C-H = 0.95Å and $U_{iso}(H) = 1.2U_{eq}(C)$ for C-H groups, and C-H = 0.98Å and $Ui_{so}(H) = 1.5U_{eq}(C)$ for CH_3 groups, while the O- and N-bound H atoms were refined freely.

The diffraction data for crystal of **5-*Ac*** was insufficient for publication therefore only a model of structure is presented.

Theoretical calculations. Geometry optimization for **6** was carried out with the Gaussian 09[1] software package within unconstrained C1 symmetry, with starting coordinates derived from X-ray analysis. Becke's three-parameter exchange functional with the gradient-corrected correlation formula of Lee, Yang and Parr (DFT-B3LYP)[2] were used with the 6- 31G(d,p) basis set. The polarizable continuum model of solvation was used (PCM, standard dichloromethane/chloroform/acetone parametrization) for all optimizations. Harmonic vibrational frequencies were calculated using analytical second derivatives as a verification of local minimum achievement with no negative frequencies observed. Wiberg indices were calculated with application of NBO analysis implemented into Gaussian package.

[1] Gaussian 09, Revision E.01; M. J. Frisch et al., Gaussian, Inc.: Wallingford CT, 2009.
[2] a) C. T. Lee, W. T. Yang, R. G. Parr, Phys. Rev. B, 1988, 37, 785-789. b) A. D. Becke, Phys. Rev. A, 1988, 38, 3098-3100.

2. Experimental section

All solvents (MeOH, Ethyl Acetate, $CHCl_3$, n-hexane, toluene, acetone, water) if not indicated differently were used without purification. CH_2Cl_2 was distilled over CaH_2. Chloroform-d was prepared directly before using by passing through a basic alumina column. All reactions were performed under inert atmosphere.

2.1 Experimental procedures

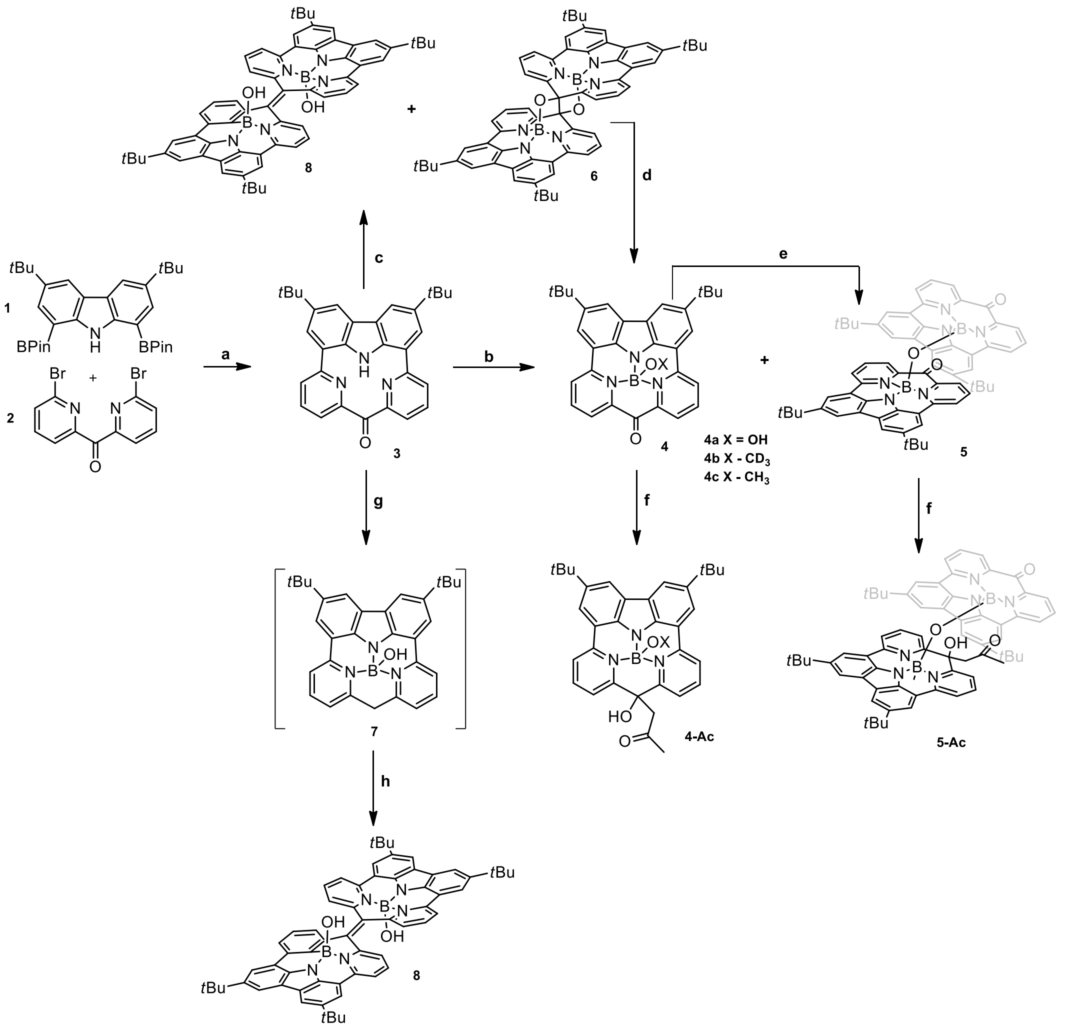

Scheme S1. *Synthetic approach. Conditions:* ***a)*** *I: 1 (1 eq.), 2 (1eq.), Pd(PPh$_3$)$_4$ (0.1 eq.), K$_2$CO$_3$ (3 eq.), KF (4 eq.), Toluene/DMF, 110 °C, 72 h;* ***b)*** *BBr$_3$ (10eq.), EtN$_3$ (13 eq.), Toluene, reflux, Ar, 2 h;* ***c)*** *BBr$_3$ (10eq.), Toluene, reflux, Ar, 2 h;* ***d)*** *MeOH (or CD$_3$OD), 4 days;* ***e)*** *Zn(Hg), CDCl$_3$, Ar, 24 h (incubation 12h , 60°C);* ***f)*** *Acetone, CF$_3$COOH (0.1 %);* ***g)*** *BBr3 (10 eq.), o-dichlorobenzene, reflux, Ar, 2 h;* ***h)*** *O$_2$, 24 h.*

Compound 1 was prepared according to previously described procedure.[3]

Compound 2[4]. In a two-necked round bottom flask 5 g (21 mmol) of 2,6-dibromopyridine was dissolved in 150 mL of THF. The mixture was degassed by pump-thaw method and then the resulting solution cooled to -78°C. A solution of *n*BuLi in hexanes (23 mmol, 9.2 mL of a 2.5 M solution) was added dropwise to the cooled solution. After a period of 5 min, a solution of diethyl carbonate (9.5 mmol, 1.15 mL in 20 mL THF) was slowly added to the solution of the lithiate. After stirring for 2 h at -78°C, the reaction was allowed to warm to ca. 0°C and quenched by adding 10% solution of HCl to reach slightly acidic pH. The resulting mixture was basified with 10% aqueous K_2CO_3, and the crude product partitioned between $CHCl_3$ and water. The aqueous layer was washed twice with $CHCl_3$, and the organic layers combined and dried with anhydrous $MgSO_4$. The resulting brown solution was decolorized with activated carbon, and the solvent removed under reduced pressure. The crude products was purified on Flash chromatography using hexane/DMC (2:8, v:v) eventually giving compound **2** as white needles in 45% yield. Mp. 155-156°C (lit. 155-156.5°C)[5]. **^{1}H NMR** (500 MHz, 300K, $CDCl_3$) δ = 8.09 (dd, ^3J = 8.0 Hz, ^4J = 0.8 Hz, 2H), 7.76 (pseudo t, ^3J = 8.0 Hz, 2H), 7.70 (dd, ^3J = 8.0, ^4J = 0.8, 2H), **^{13}C NMR** (150 MHz, 300K, $CDCl_3$) δ = 189.2, 154.1, 141.6, 139, 131.5, 124.3; **HRMS** (ESI) *m/z*: $[M+Na]^+$ Calcd for $C_{11}H_6Br_2N_2ONa$ 364.8719; Found 364.8702.

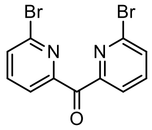

Compound 3. In 100 mL two-necked round bottom flask, was charged with **1** (0.16 g, 0.3 mmol), tetrakis(triphenylphosphine)palladium(0) (0.034 g, 0.3 mmol, 0.1 eq.), potassium fluoride (0.068 g, 1.18 mmol, 4 eq.), potassium carbonate (0.12 g, 0.88 mmol, 3 eq.) and **2** (0.1 g, 0.3 mmol, 1 eq.). The mixture was dried under vacuum overnight. Then 20 mL of dry toluene and 20 mL of dry DMF were added via syringe. The mixture was then degassed by pump-thaw method (or bubble the solution with Ar) and stirred in 110°C on heating mantle for 96 h under Ar. After cooling to room temperature, the solution was diluted with ethyl acetate and passed through pad silica gel. After evaporation to dryness product was purified with silica gel (hexane:DCM, 1:1, v:v) to give an yellow solid as the desired compound. The product was crystalized using DMC/MeOH (yield 20%, 41 mg).**^{1}H NMR** (500 MHz, 300K, $CDCl_3$) δ = 18.48 (s, 1H), 8.20 – 8.16 (m, 4H), 8.19 (d, ^4J = 1.3 Hz, 2H), 8.02 – 8.07 (m, 2H), 7.93 (d, ^4J = 1.3 Hz, 2H), 1.53 (s, 18H), **^{13}C NMR** (125.75 MHz, 300K, $CDCl_3$) δ = 187.1, 155.7, 152.1, 143.3, 142, 138.1, 125.5, 122.6, 122.4, 120.5, 119.6, 118.2, 25.3, 32.5; **HRMS** (ESI) *m/z*: $[M+H]^+$ Calcd for $C_{31}H_{30}N_3O$ 460.2383; Found 460.2367; **UV-Vis**: 338, 411 nm, **Extinction**: $0.93*10^4$ $M^{-1}cm^{-1}$, **X-ray** – SI chapter 7.

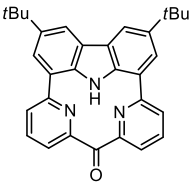

Procedures for boron insertion:

Procedure 1 (see Fig. S79): 15 mL of solution of **3** in toluene (10 mg, 0.022 mmol, 1 eq.) was placed in a 25 mL two-necked round-bottom flask and argon was bubbled through the solution for 15 min. Next, the mixture was refluxed for 15 min using heating mantle and then BBr_3 was added (21 µl, 0.21 mmol, 10 eq.) and refluxed for 15 min with the same source of heating. After that time Et_3N (30 µl, 0.21 mmol, 10 eq.) was added and the reflux condition was continued for 2 h using heating mantle. After completion, the brown solid was separated, extracted with chloroform, washed with water, dried over Na_2SO_4 and evaporated. The crude product was purified by a preparative reversed-phase HPLC on a Vydac C18 column (22 mm × 250 mm), using solvent systems: S1: 0.1% aqueous TFA, S2: acetonitrile + 0.1% TFA, linear gradient was individually set for compound, flow rate 7.0 mL/min, UV detection at 280 nm. The fractions of compounds **4a** and **5** and were collected and evaporated.

Procedure 2 (See Fig. S80): 15 mL of solution of **3** in toluene (10 mg, 0.022 mmol, 1 eq.) was placed in a 25 mL two-necked round-bottom flask and argon was bubbled through the solution for 15 min. Next, the mixture was refluxed using heating mantle for 15 min and then BBr_3 was added (21 µl, 0.21 mmol, 10 eq.) and refluxed using heating mantle for 2 h. After completion, the brown solid was separated,

---

[3] Arnold, L et. al. *Chem. Commun.* **2011**, *47*, 970-972; (b) Maeda, Ch.; et al. *Angew. Chem. Int. Ed.* **2011**, *50*, 5691-5694.

[4] Tetrahedron, **2001**, 57, 1175-1182

[5] J. Am. Chem. Soc., **1996**, 118, 5783-5790.

extracted with chloroform, washed with water, dried over Na_2SO_4 and evaporated. The crude product was purified by a preparative reversed-phase HPLC on a Vydac C18 column (22 mm × 250 mm), using solvent systems: S1: 0.1% aqueous TFA, S2: acetonitrile + 0.1% TFA, linear gradient was individually set for compound, flow rate 7.0 mL/min, UV detection at 280 nm. The fractions of compounds **4a**, **6** and **8** were collected and evaporated.

Procedure 2a (see Fig. S95): 15 mL of solution of **3** in toluene (10 mg, 0.022 mmol, 1 eq.) was placed in a 25 mL two-necked round-bottom flask and argon was bubbled through the solution for 15 min. Next, the mixture was refluxed using heating mantle for 15 min and then BBr_3 was added (21 µl, 0.21 mmol, 10 eq.) and refluxed using heating mantle for 12 h. After completion, the brown solid was separated, extracted with chloroform, washed with water, dried over Na_2SO_4 and evaporated. The crude product was analyzed by LC-MS t on an RP-Zorbax (50×2.1 mm, 3.5 µm) column with a gradient elution of 0-80% B in A (A = 0.1% HCOOH in water; B = 0.1% HCOOH in MeCN) at room temperature over a period of 20 min (flow rate: 0.1 mL/min). LC-MS analysis revealed compounds **6 (62%)**, **4a (13 %)**, **7 (18%)** and **8 (7%)**. The composition of obtained compounds was calculated based on LC-MS spectrum.

Procedure 3 (see Fig. S96-97): 15 mL of solution of **3** in *o*-dichlorobenzene (10 mg, 0.022 mmol, 1 eq.) was placed in a 25 mL two-necked round-bottom flask and argon was bubbled through the solution for 15 min. Next, the mixture was refluxed using heating mantle for 15 min and then BBr_3 was added (21 µl, 0.21 mmol, 10 eq.) and refluxed using heating mantle for 2 h. After completion, the brown solid was separated, extracted with chloroform, washed with water, dried over Na_2SO_4 and evaporated. The crude product (monomer monoisotopic mass 472.253) was purified by a preparative reversed-phase HPLC on a Vydac C18 column (22 mm × 250 mm), using solvent systems: S1: 0.1% aqueous TFA, S2: acetonitrile + 0.1% TFA, linear gradient was individually set for compound, flow rate 7.0 mL/min, UV detection at 280 nm. The fraction of unstable product **7** was collected and evaporated. Then the product **7** was analyzed by LC-MS t on an RP-Zorbax (50×2.1 mm, 3.5 µm) column with a gradient elution of 0-80% B in A (A = 0.1% HCOOH in water; B = 0.1% HCOOH in MeCN) at room temperature over a period of 20 min (flow rate: 0.1 mL/min). LC-MS analysis revealed compounds **4a** (41%) and **8** (59%).

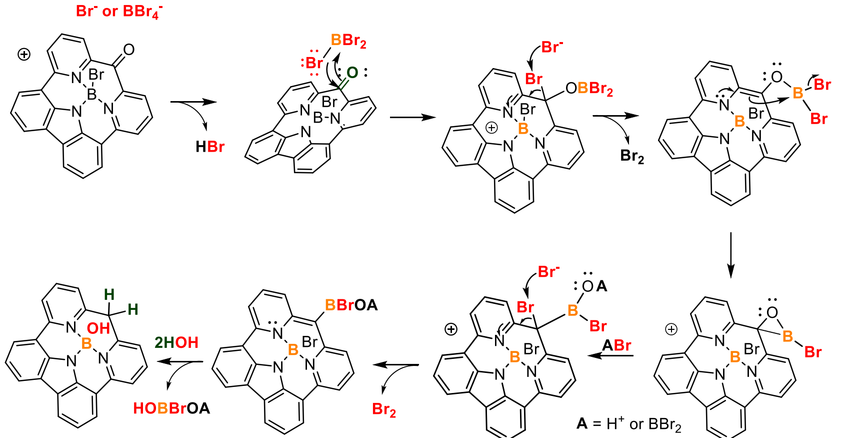

Scheme S2. Potential mechanism for C=O reduction observed for **3** and activated by BBr_3.

General procedure of reduction: 2 mg of compound **4a** was dissolved in 500 µL of $CDCl_3$ in a glove-box conditions. To this solution 50 eq. (w/w) of zinc amalgam was added and the reaction was controlled by LC-MS analysis and NMR spectroscopy.

Compound 4a. Procedure 1. Yield of isolated product: 28% (2.9 mg). **^{1}H NMR** (600 MHz, 300K, $(CD_3)_2CO$) δ = 9.53 (dd, ^3J = 8.5 Hz, ^4J = 0.9 Hz, 2H), 8.97 - 8.93 (m, 2H), 8.88 (d, ^4J = 1.2 Hz, 2H), 8.81 (d, ^4J = 1.2 Hz, 2H), 8.82 (dd, ^3J = 7.64 Hz, ^4J = 0.9 Hz, 2H), 1.60 (s, 18H), **^{13}C NMR** (150 MHz, 300K, $(CD_3)_2CO$) partial signals obtained by correlation experiments HMBC, HSQC, δ = 177.0, 148.6, 146.1, 143.7, 143.5, 138.3, 127.6, 126.3, 125.4, 125.1, 121.7, 112, 35.7, 31.6; **HRMS** (ESI) *m/z*: $[M]^+$ Calcd for

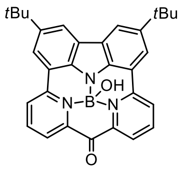

$C_{31}H_{29}BN_3O_2$ 486.2353; Found 486.2330; **UV-Vis**: 313, 342, 438 nm; **Extinction**: 1.02*10^4 $M^{-1}cm^{-1}$, **Emission**: 506 nm, **X-ray** – SI chapter 7.

Compound 4b. 3 mg of compound **6** was dissolved in 500 µL of CD_3OD in a glove-box conditions. The conversion of product **6** resulting in product **4b** was monitored using NMR and LC-MS equipment.[6] **^{1}H NMR** (500 MHz, 300K, CD_3OD) δ = 9.33 (dd, ^3J = 8.56 Hz, ^4J = 0.93 Hz, 2H), 8.83 – 8.79 (m, 2H), 8.81 (d, ^4J = 1.2 Hz, 2H), 8.67 (d, ^4J = 1.2 Hz, 2H), 8.73 (dd, ^3J = 7.63 Hz, ^4J = 0.96 Hz, 2H), 1.67 (s, 18H), **^{13}C NMR** (150 MHz, 300K, CD_3OD) δ =176, 148.8, 146.6, 143.8, 143.5, 138.5, 126.8, 126.5, 125.2, 124.9, 121.4, 111.6, 48.2, 35.5, 31.2; **HRMS** (ESI) *m/z*: $[M]^+$ Calcd for $C_{32}H_{28}D_3BN_3O_2$ 503.2698; Found 503.2671; **UV-Vis**: 313, 342, 438 nm. **Compound 4c (X = CH_3). HRMS** (ESI) *m/z*: $[M]^+$ Calcd for $C_{32}H_{31}BN_3O_2$ 500.2509; Found 500.2495; **X-ray** – SI chapter 7.

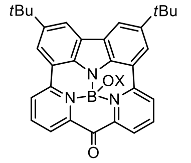

Compound 4-Ac. 1 mg of compound **4** was dissolved in 500 µl of $(CD_3)_2CO$ in NMR tube at RT. The sample was monitored by NMR in time. The compound **4**-*Ac* appeared after one month of incubation.) **^{1}H NMR:** (500 MHz, 300K, $(CD_3)_2CO$) δ = 9.55 (dd, ^3J = 8.6 Hz, ^4J = 0.9 Hz, 1H), 9.13 (dd, ^3J = 7.9 Hz, ^4J = 0.9 Hz, 1H), 8.99-8.95 (m, 1H), 8.90 (d, ^4J = 1.2 Hz, 1H), 8.85-8.82 (m, 2H), 8.77-8.72 (m, 1H), 8.65 (d, ^4J = 1.1 Hz, 1H), 8.52 (dd, ^3J = 7.6 Hz, ^4J = 0.94 Hz, 1H), 8.46 (d, ^4J = 1.0 Hz, 1H), 1.65 (s, 9H), 1.62 (s, 9H); **HRMS** (ESI) *m/z*: $[M]^+$ Calcd for $C_{34}H_{30}D_5B_2N_3O_3$ 549.3086; Found 549.3020; **X-ray** – SI chapter 7.

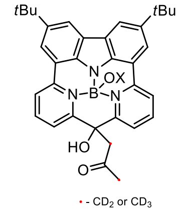

Compound 5. (Procedure 1) Yield of isolated product: 10.5% (1.1 mg); (**General procedure of reduction**) Yield: 45 %. **^{1}H NMR** (500 MHz, 300K, $(CD_3)_2CO$) δ = 9.16 (d, ^3J = 8.46 Hz, 4H), 8.76 - 8.72 (m, 4H), 8.64 (d, ^4J = 1.0, 4H), 8.50 (d, ^3J = 7.47 Hz, 4H), 8.48 (d, *J* = 1.08 Hz, 4H), 1.65 (s, 36H); **HRMS** (ESI) *m/z*: $[M]^{2+}$ Calcd for $C_{62}H_{58}B_2N_6O_3$ 477.2303; Found 477.2288; **UV-Vis**: 309, 443.

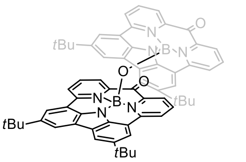

Compound 5-Ac. Obtained quantitatively from compound **5** in acetone at RT. **^{1}H NMR:** (500 MHz, 300K, $(CD_3)_2CO$) δ = 9.16 (d, ^3J = 8.04 Hz, 1H), 8.72 - 8.68 (m, 1H), 8.58 (d, ^4J = 0.9 Hz, 1H), 8.56 (d, ^4J = 1.1 Hz, 1H), 8.5 (dd, ^3J = 7.5 Hz, ^4J = 0.8 Hz, 1H), 8.39 (d, ^4J = 1.0 Hz, 1H), 8.38-8.35 (m, 1H), 8.34 (d, ^4J = 1.1 Hz, 1H), 8.26 (dd, ^3J = 7.9 Hz, ^4J = 0.9 Hz, 1H), 8.23 (d, ^4J = 1.0 Hz, 1H), 1.67 (s, 1H), 1.66 (s, 1H), **^{13}C NMR** (150 MHz, 300K, $(CD_3)_2CO$) partial signals obtained by correlation experiments HMBC, δ = 176.1, 157.5, 147.5, 146.2, 145.9, 145.4, 142.5, 142.4, 138.1, 136.8, 127.2, 126, 125.2, 125, 124.3, 124.2, 123.9, 121.8, 121.4, 120.7, 111.9, 111.3, 75.4, 35.6, 35.5, 31.8, 31.7; **HRMS** (ESI) *m/z*: $[M]^{2+}$ Calcd for $C_{65}H_{57}D_5B_2N_6O_4$ 508.7670; Found 508.7652; **UV-Vis**: 309, 443 nm, **X-ray** – SI chapter 7.

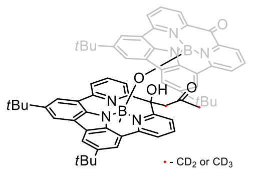

Compound 6 (Procedure 2). Yield of isolated product: 35% (3.6 mg).**^{1}H NMR** (500 MHz, 300K, $(CD_3)_2CO$) δ = 9.1 (d, ^3J = 8.3 Hz, 4H), 8.85 (d, ^4J = 1.25, 4H), 8.62 (d, ^4J = 1.1 Hz, 4H), 8.56 - 8.51(m, 4H), 8.17 (d, ^3J = 7.4 Hz, 4H), 1.60 (s, 36H), **^{13}C NMR** (150 MHz, 300K, $(CD_3)_2CO$) partial signals obtained by correlation experiments HMBC, δ = 149.5, 147.6, 147.4, 145.3, 142.5, 127.6, 125.8, 122.5, 121.3, 119.7, 112.8, 84.5, 35.5, 31.4; **HRMS** (ESI) *m/z*: $[M]^{2+}$ Calcd for $C_{62}H_{56}B_2N_6O_2$ 469.2329; Found 469.2311; **UV-Vis**: 309, 443 nm, **Extinction**: 1.8*10^4 $M^{-1}cm^{-1}$, **Emission**: 510 nm, **X-ray** – SI chapter 7.

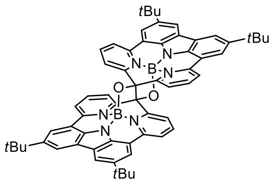

Compound 8 (Procedure 2). Yield of isolated product: 19.5% (2 mg).**^{1}H NMR** (500 MHz, 300K, $(CD_3)_2CO$) δ = 9.07 (d, ^3J = 8.2 Hz, 4H), 8.77 (d, ^4J = 1.08, 4H), 8.67 (d, ^4J = 1.1 Hz, 4H), 8.42 - 8.38 (m, 4H), 8.33 (d, ^3J = 7.47 Hz, 4H), 1.60 (s, 36H); **HRMS** (ESI) *m/z*: $[M]^{2+}$ Calcd for $C_{62}H_{58}B_2N_6O_2$ 470.2407; Found 470.2389; **UV-Vis**: 345, 443, 522 nm, **Extinction**: 1.9*10^4 $M^{-1}cm^{-1}$, **Emission**: 511 nm, **X-ray** – SI chapter 7.

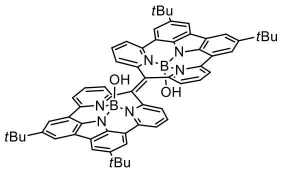

[6] For 0.5 mg of compound **6** dissolved in 0.5 ml of MeOH in a glove-box conditions, the product **4c** was monitored using LC-MS equipment.

3. NMR spectra

3.1 NMR spectra of 2

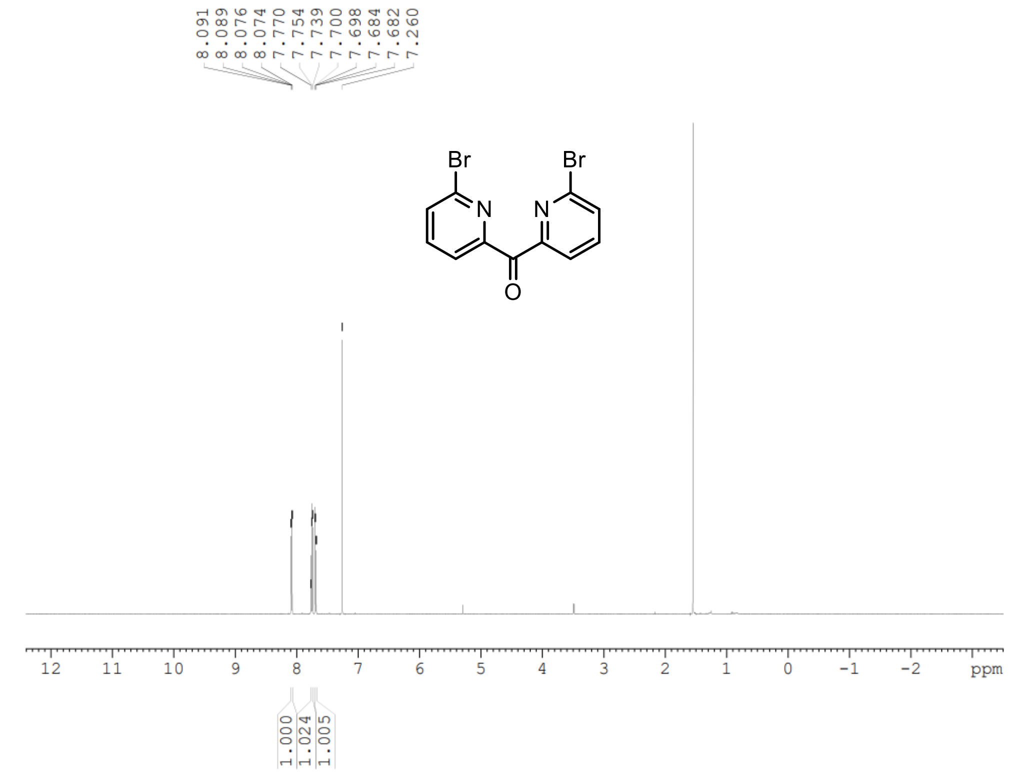

Figure S1. ^{1}H NMR spectrum of **2** ($CDCl_3$, 300K, 500 MHz)

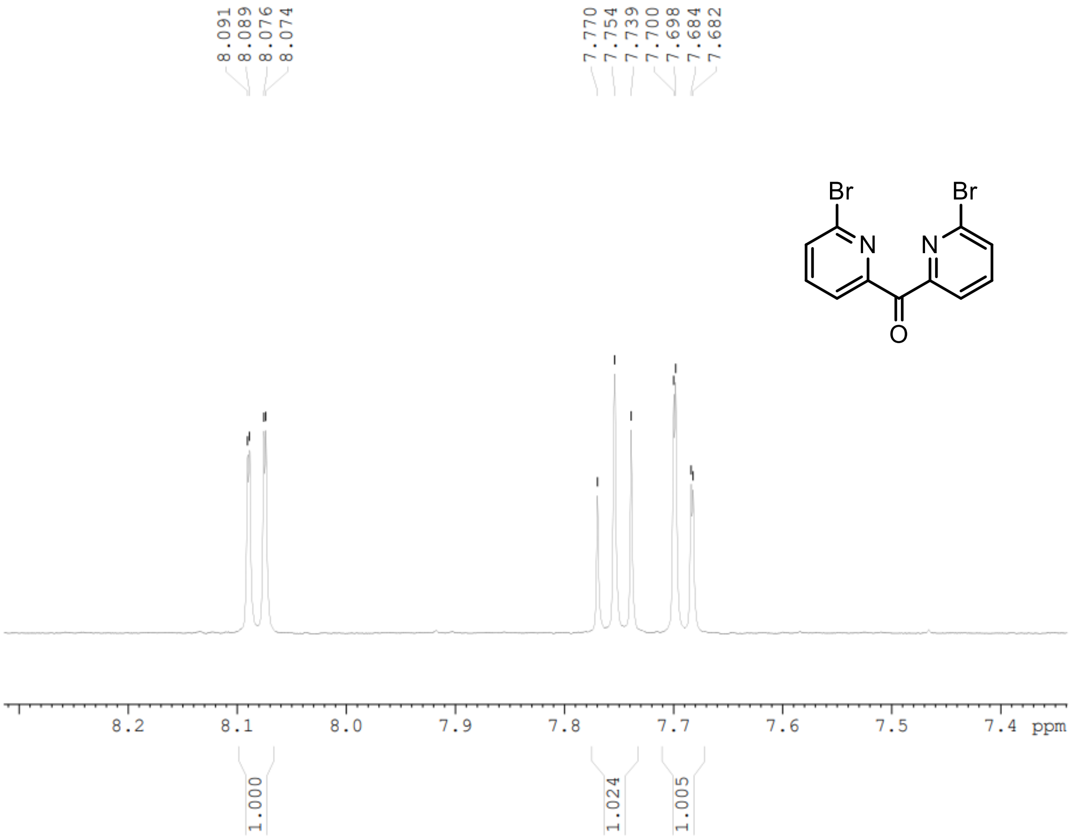

Figure S2. ^{1}H NMR spectrum (zoom, aromatic region) of **2** ($CDCl_3$, 300K, 500 MHz).

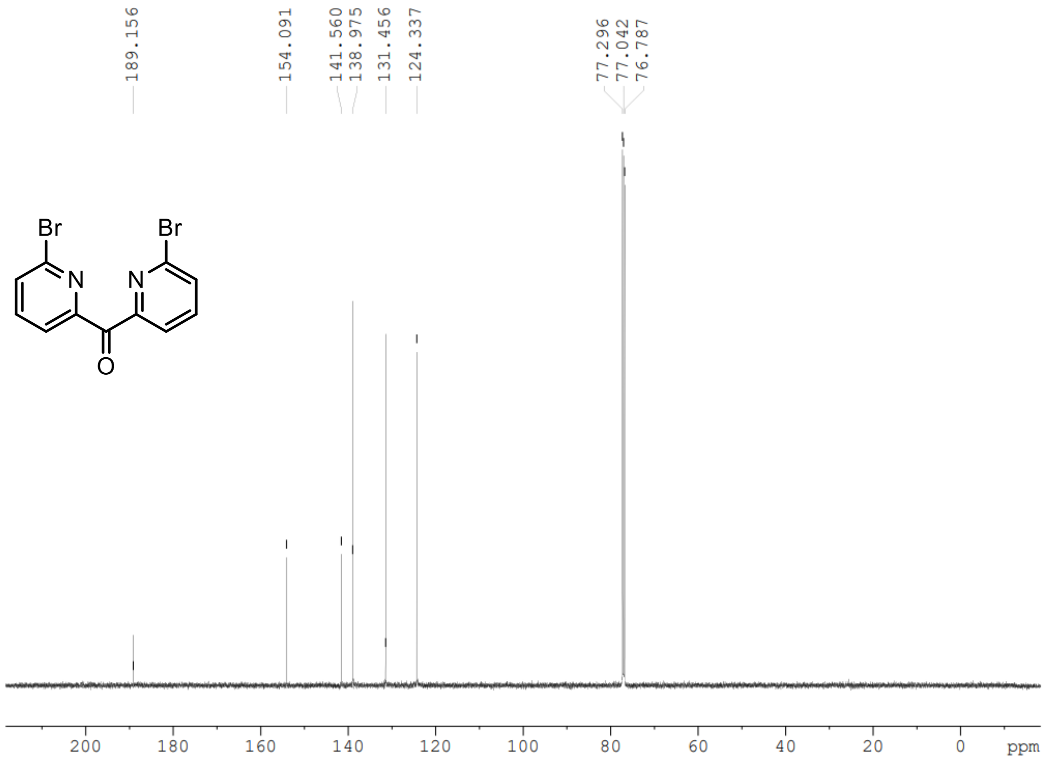

Figure S3. ^{13}C NMR spectrum of **2** ($CDCl_3$, 300K, 150 MHz)

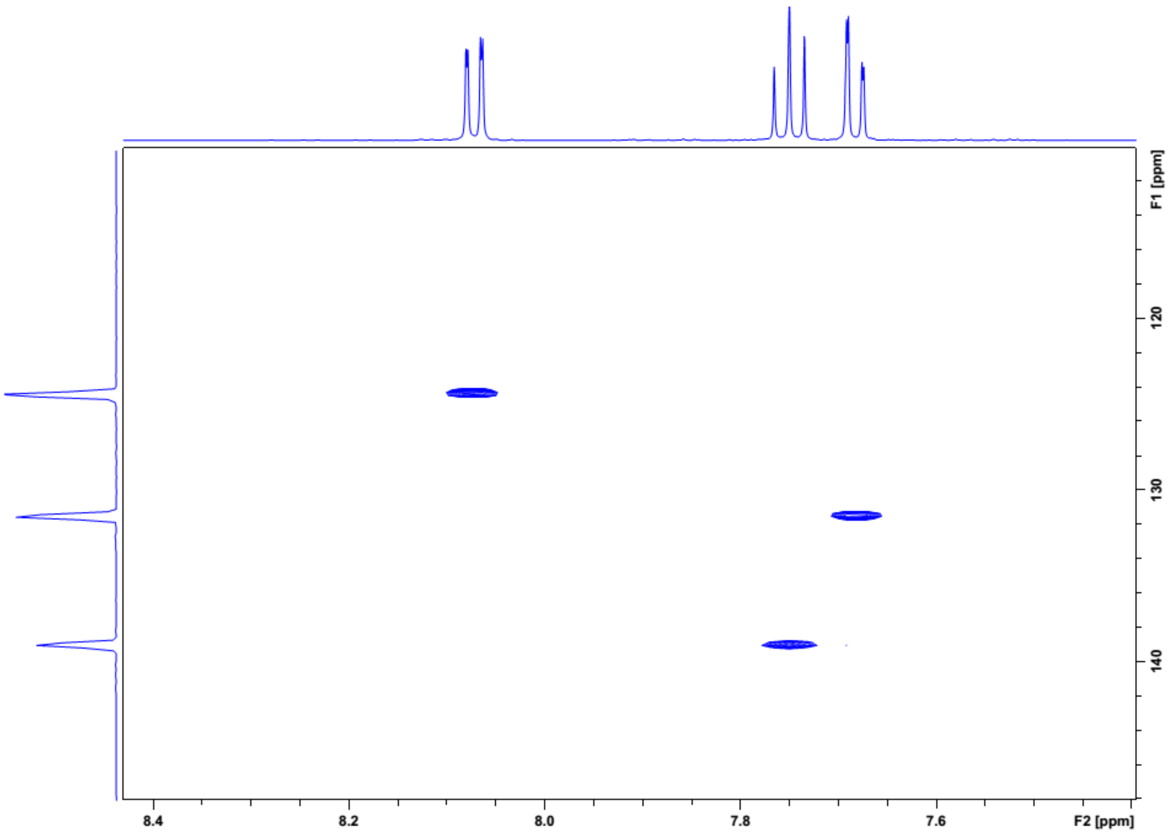

Figure S4. HSQC NMR spectrum of 2 ($CDCl_3$, 300K, 500 MHz)

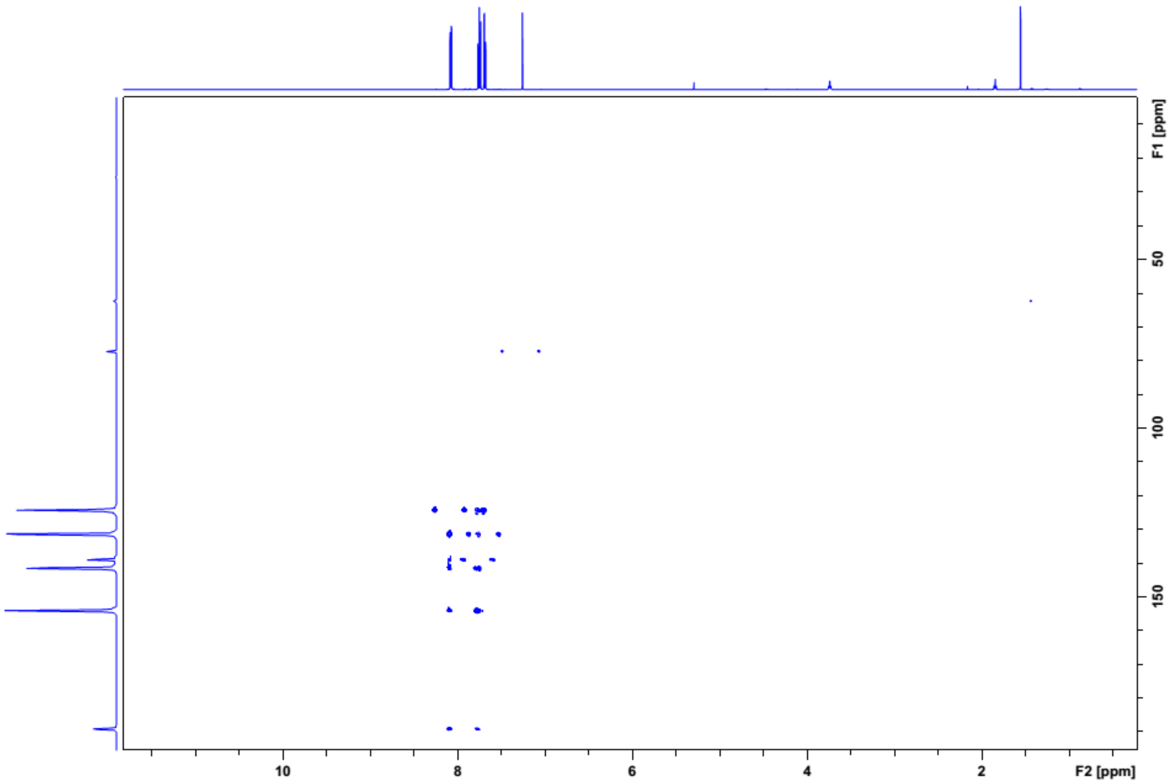

Figure S5. HMBC NMR spectrum of **2** ($CDCl_3$, 300K, 500 MHz)

3.2 NMR spectra of 3

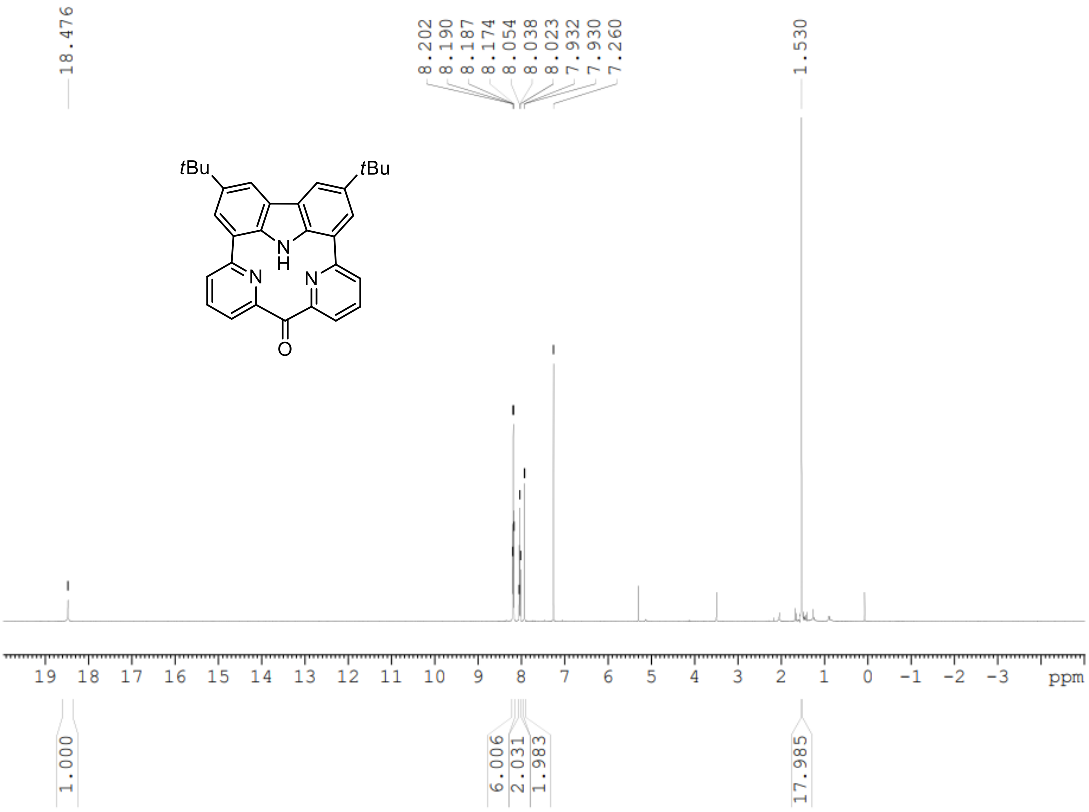

Figure S6. ^{1}H NMR spectrum of **3** ($CDCl_3$, 300K, 500 MHz)

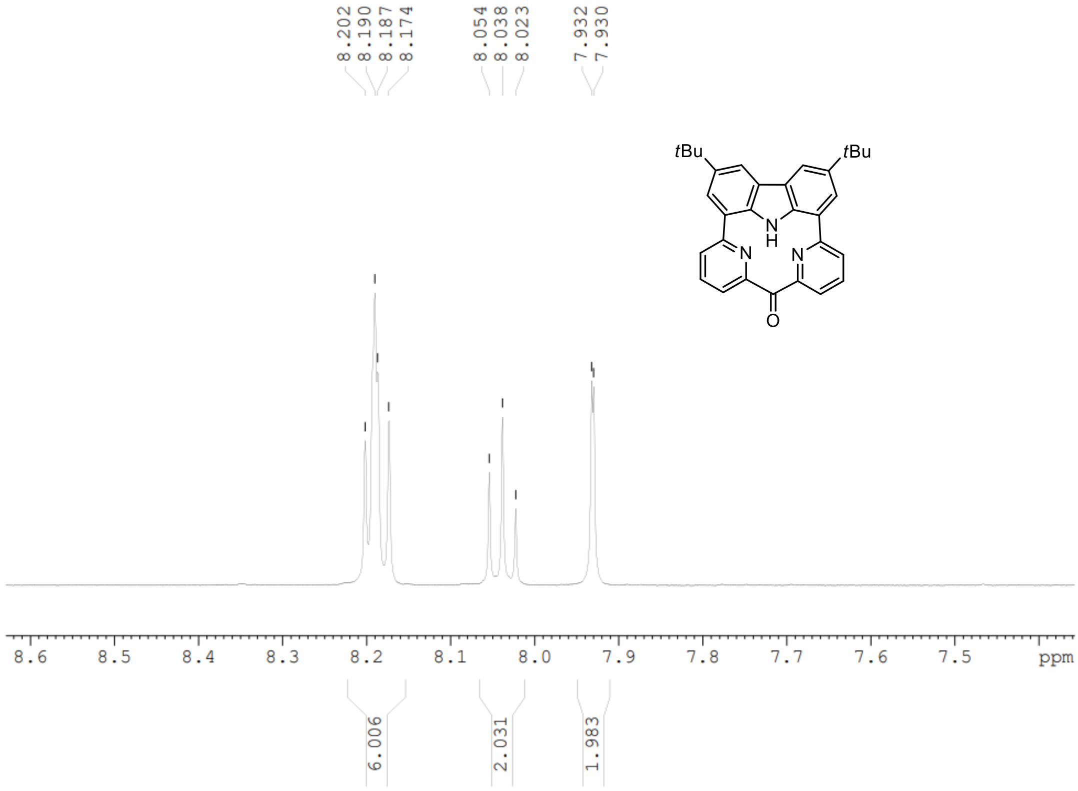

Figure S7. ^{1}H NMR spectrum (zoom, aromatic region) of **3** ($CDCl_3$, 300K, 500 MHz).

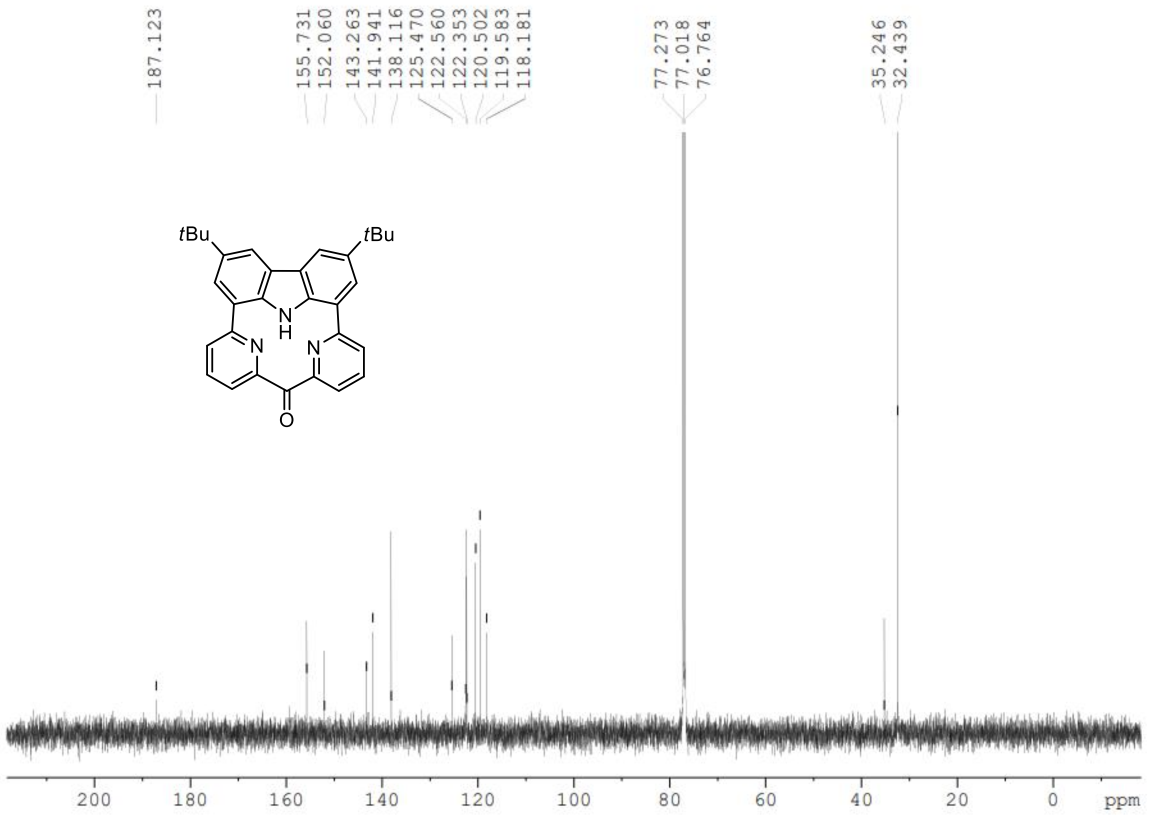

Figure S8. ^{13}C NMR spectrum of **3** ($CDCl_3$, 300K, 150 MHz).

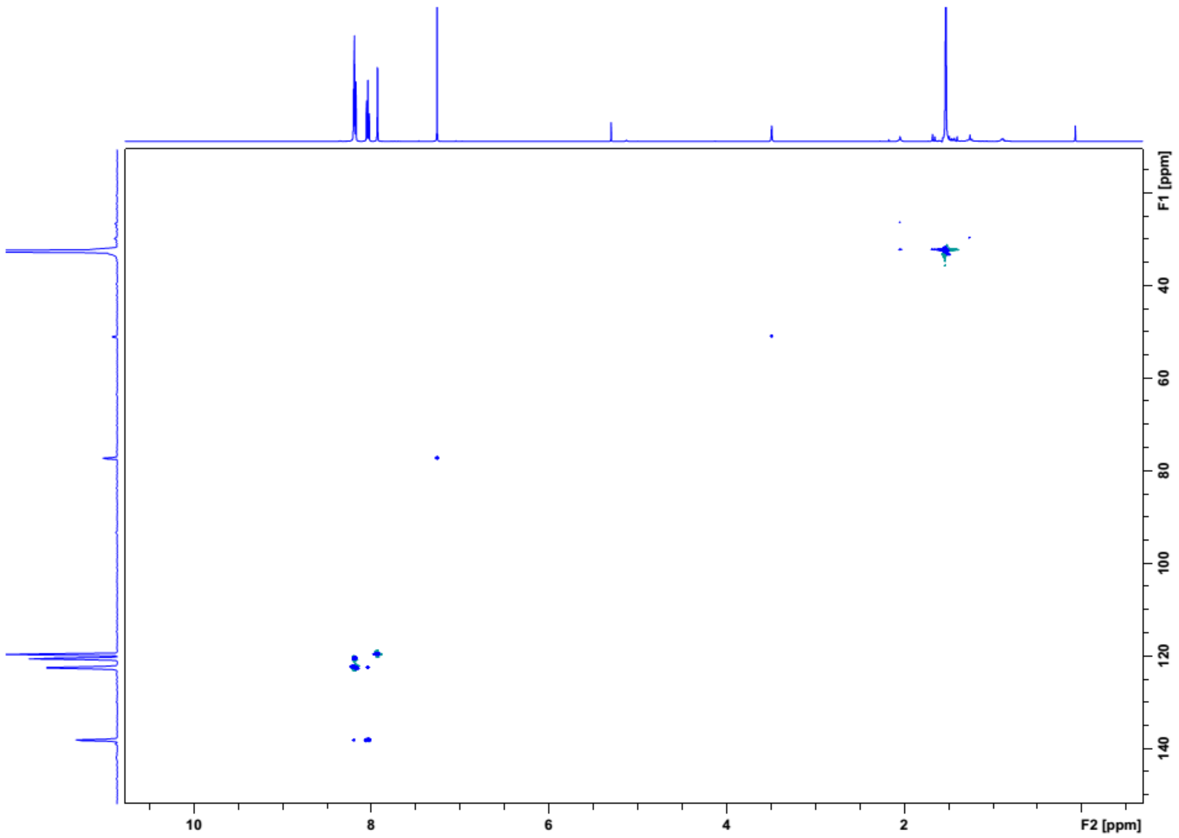

Figure S9. HSQC NMR spectrum of **3** ($CDCl_3$, 300K, 500 MHz).

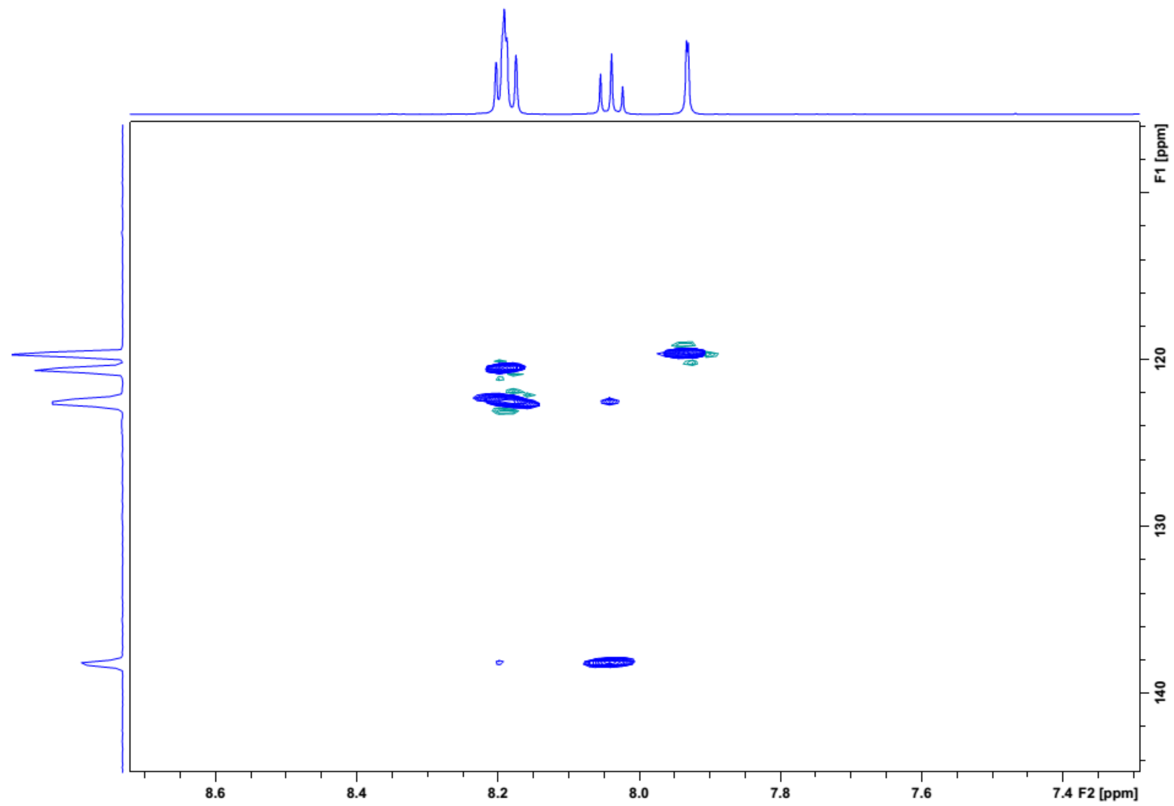

Figure S10. HSQC NMR spectrum (zoom, aromatic region) of **3** ($CDCl_3$, 300K, 500 MHz).

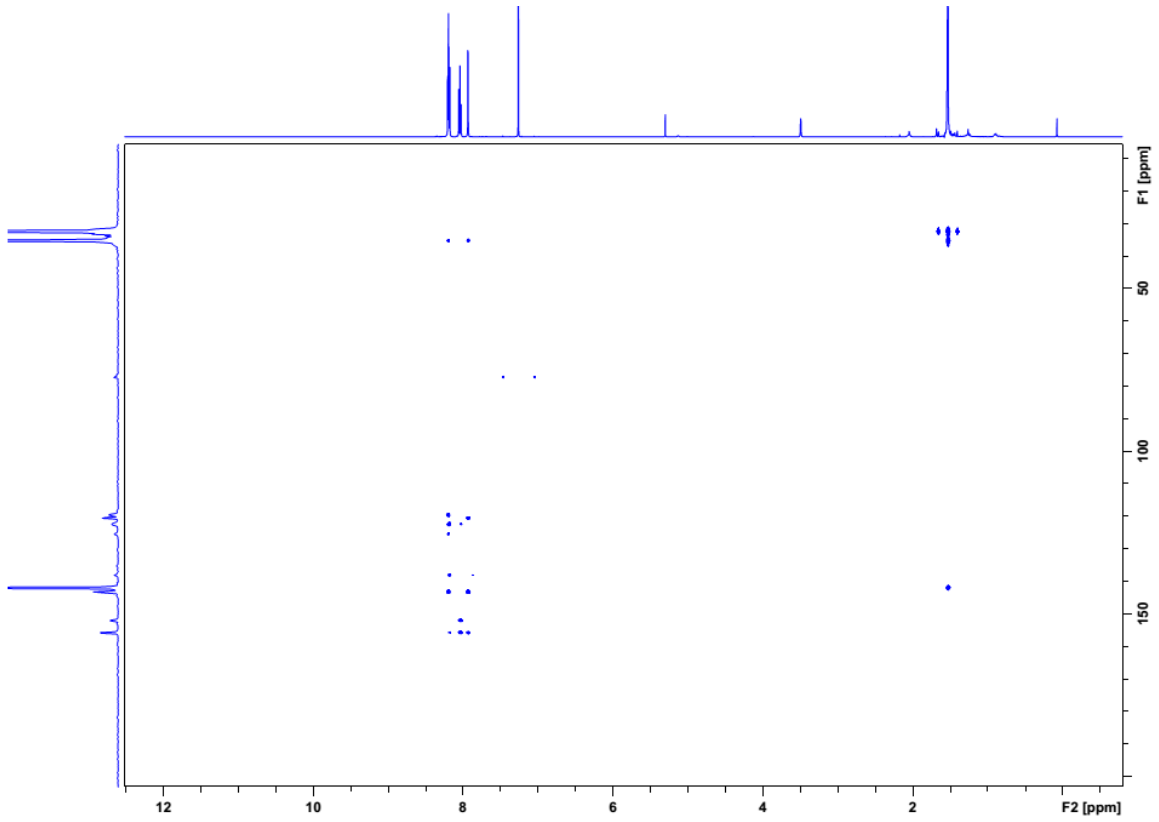

Figure S11. HMBC NMR spectrum of **3** ($CDCl_3$, 300K, 500 MHz).

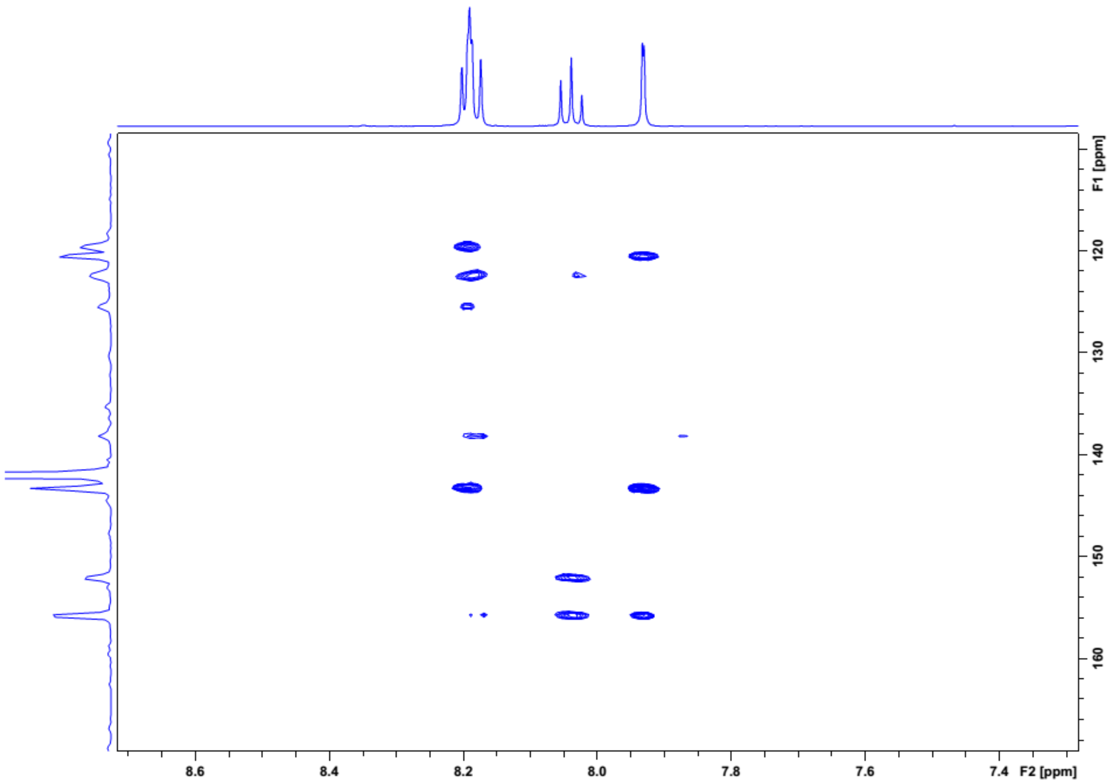

Figure S12. HMBC NMR spectrum (zoom, aromatic region) of **3** ($CDCl_3$, 300K, 500 MHz).

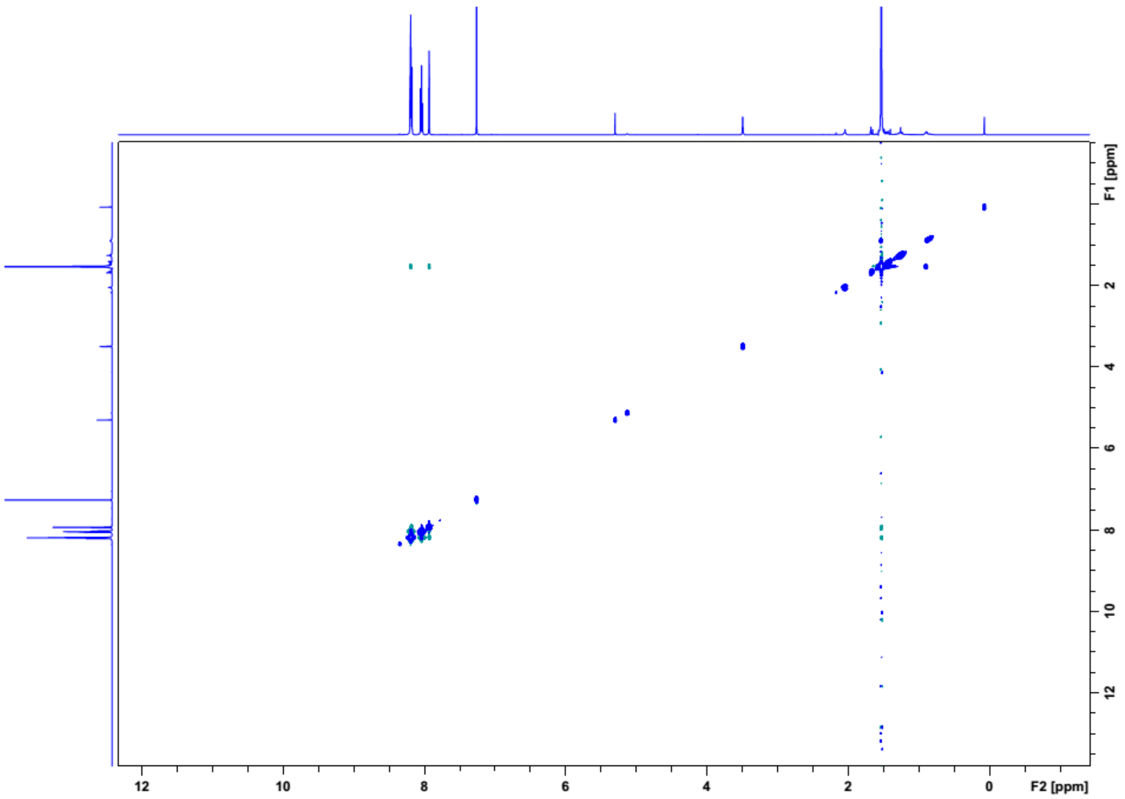

Figure S13. NOESY NMR spectrum of **3** ($CDCl_3$, 300K, 500 MHz).

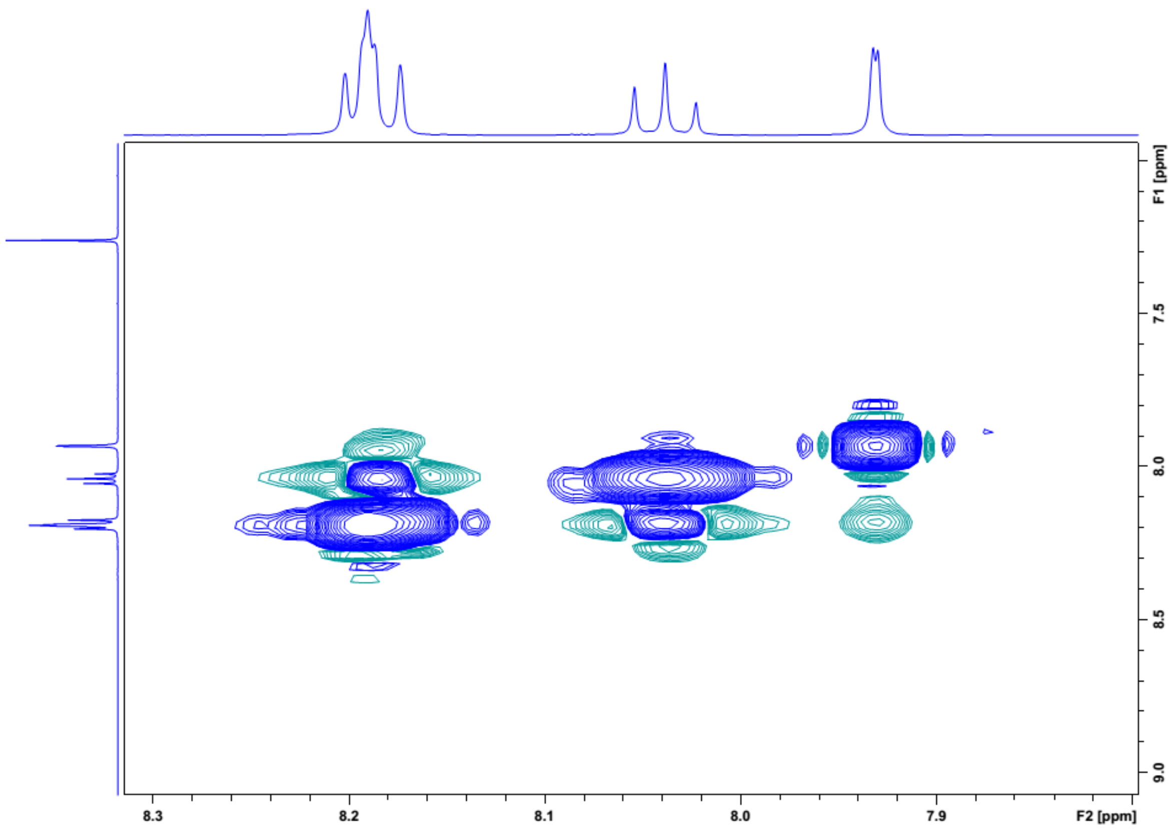

Figure S14. NOESY NMR spectrum (zoom, aromatic region) of **3** ($CDCl_3$, 300K, 500 MHz).

3.3 NMR spectra of 4a

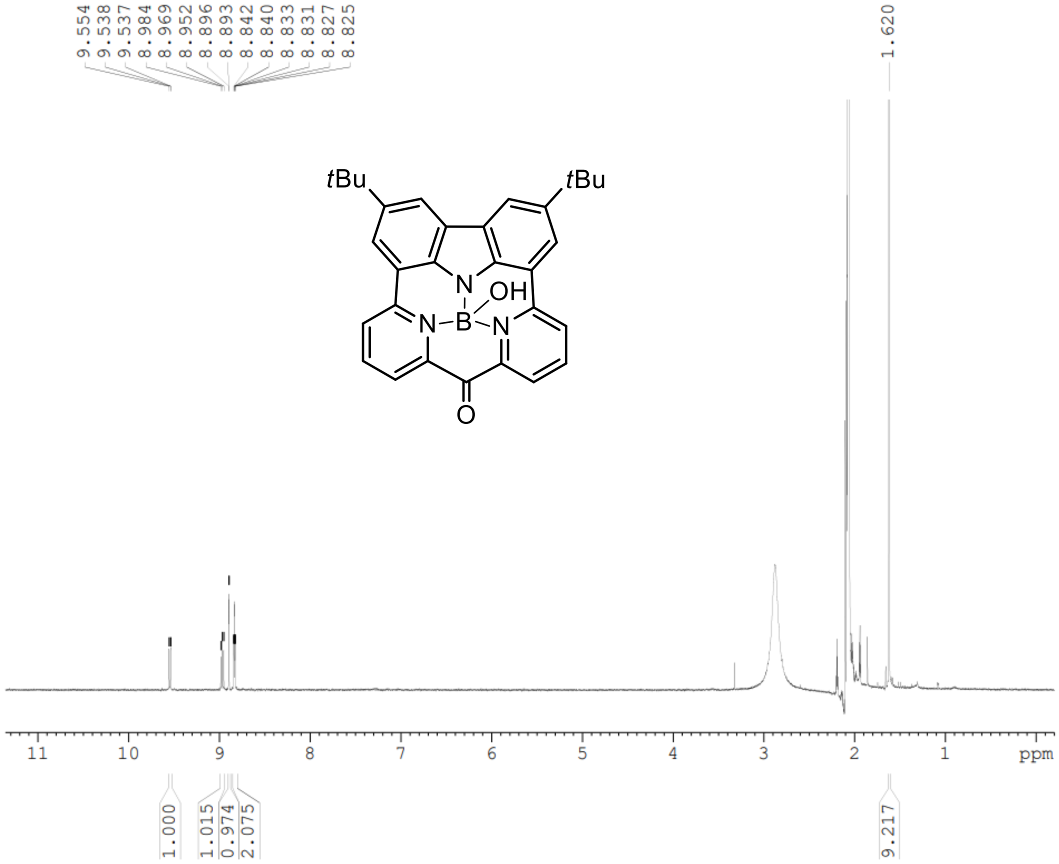

Figure S15. ^{1}H NMR spectrum of **4a** ($(CD_3)CO$, 300K, 500 MHz)

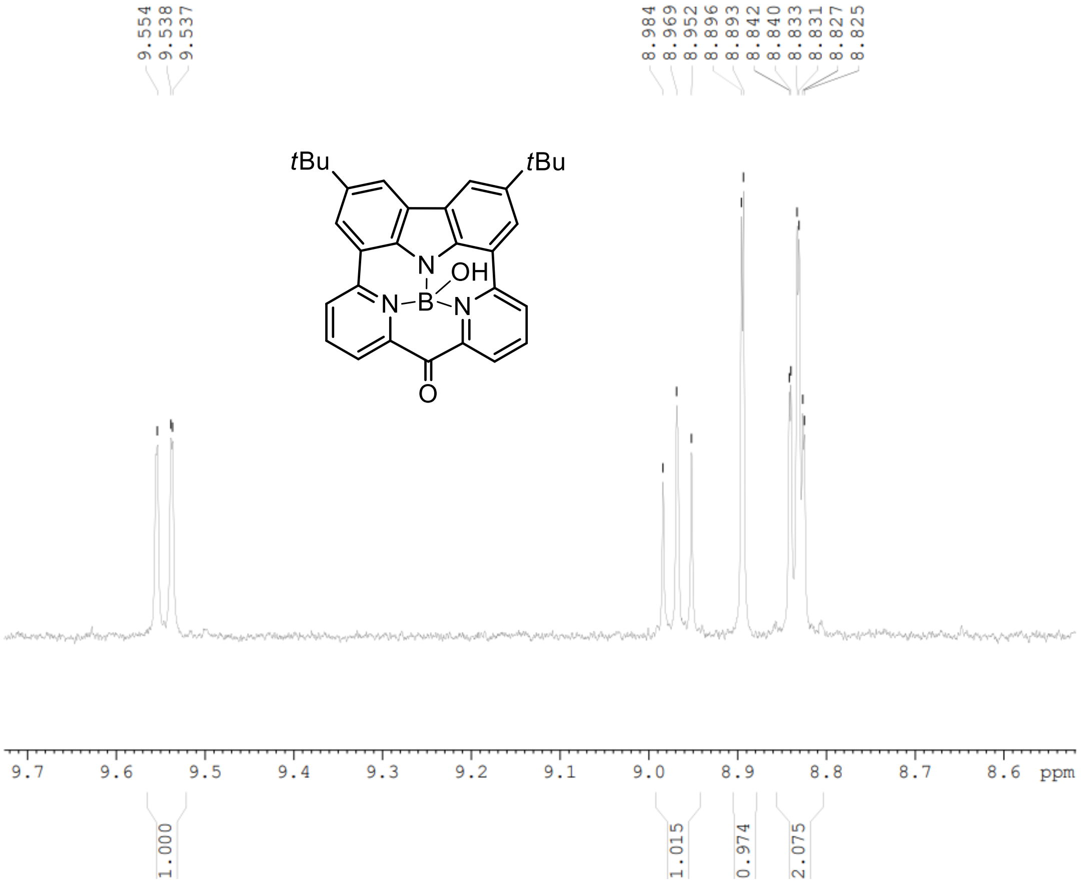

Figure S16. ^{1}H NMR spectrum (zoom, aromatic region) of **4a** ($(CD_3)CO$, 300K, 500 MHz).

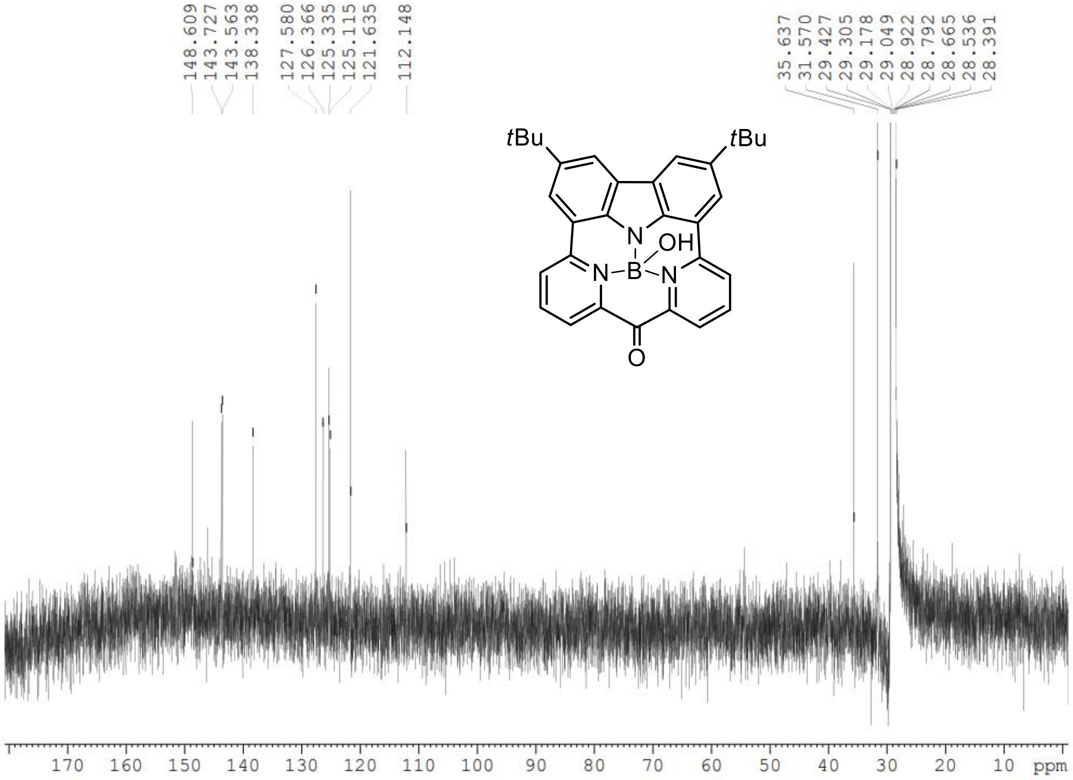

Figure S17. ^{13}C NMR spectrum of **4a** ($(CD_3)CO$, 300K, 150 MHz).

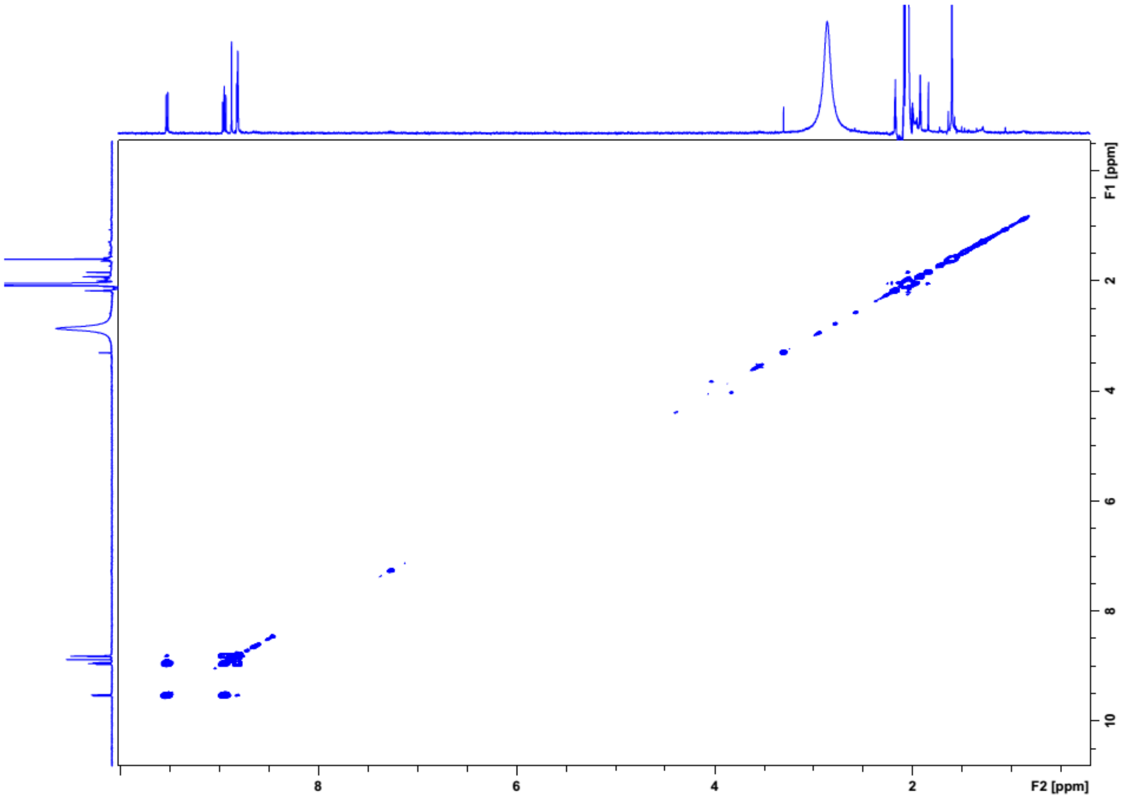

Figure S18. COSY spectrum of **4a** ($(CD_3)CO$, 300K, 500 MHz).

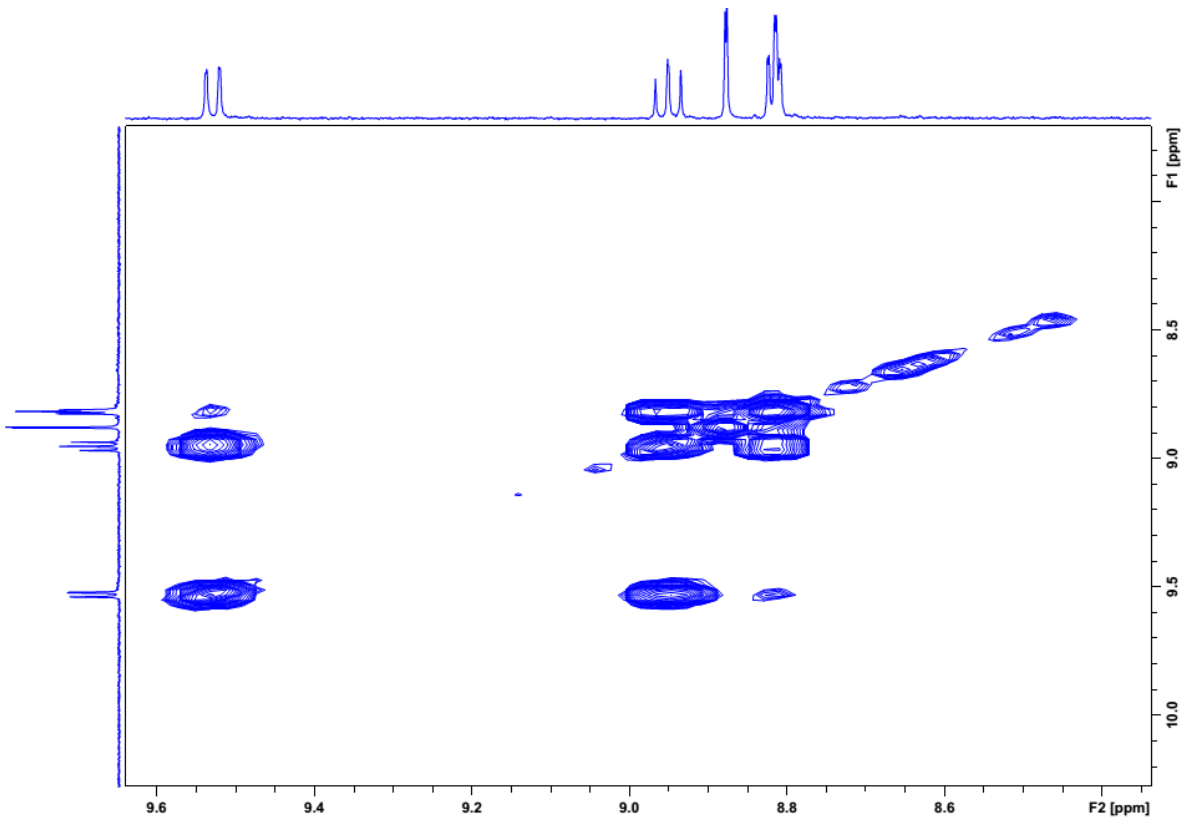

Figure S19. COSY spectrum (zoom, aromatic region) of **4a** ($(CD_3)CO$, 300K, 500 MHz).

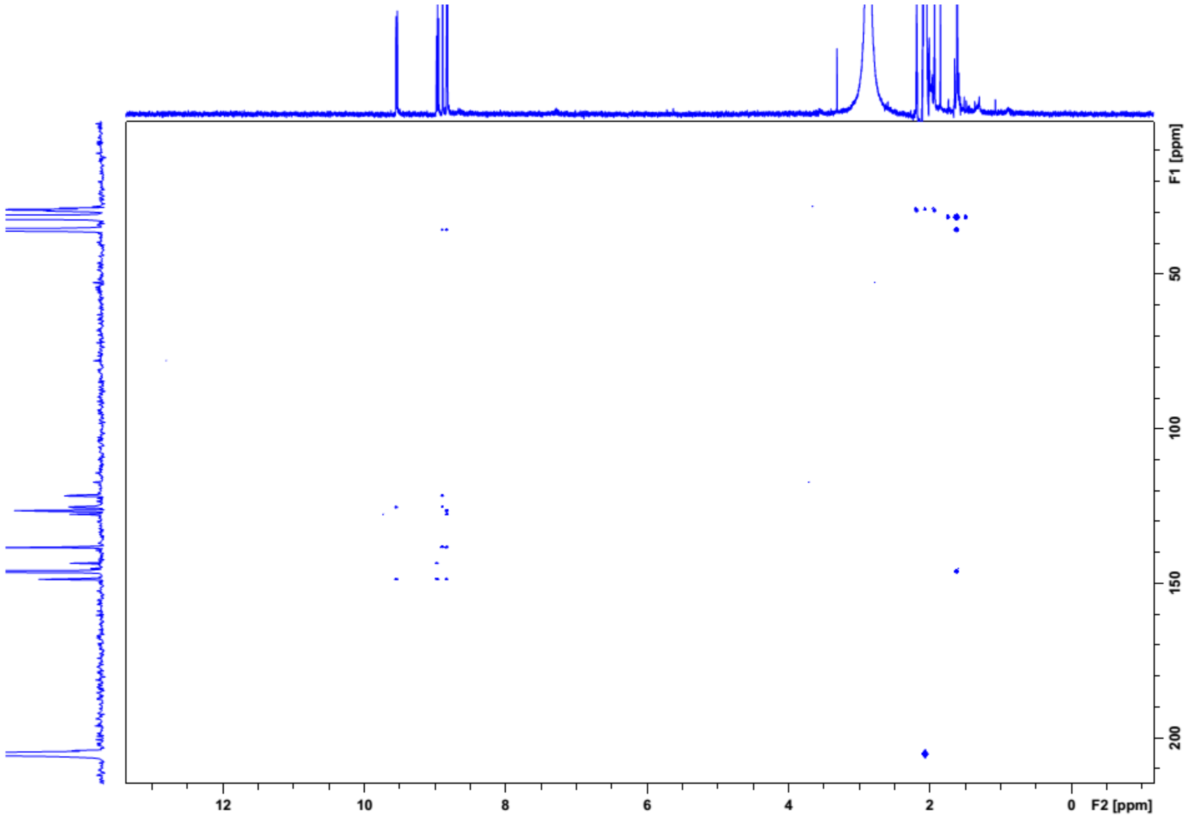

Figure S20. HMBC spectrum of **4a** ($(CD_3)CO$, 300K, 500 MHz).

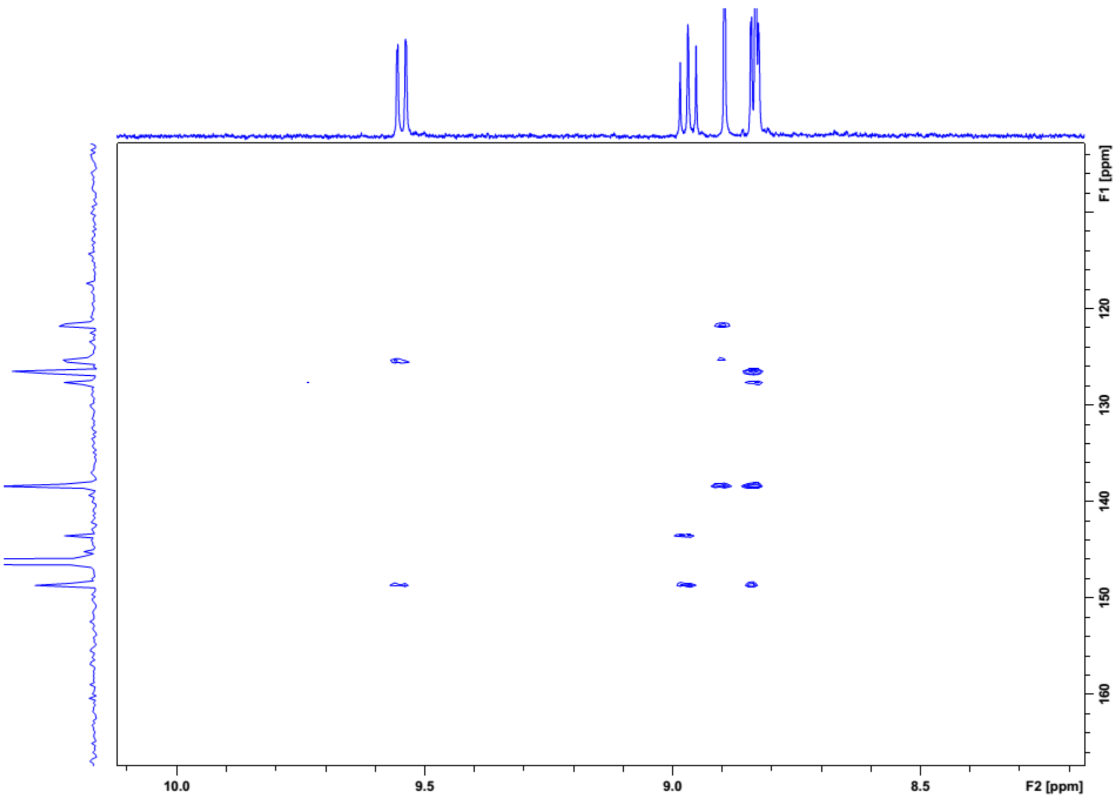

Figure S21. HMBC spectrum (zoom, aromatic region) of **4a** ($(CD_3)CO$, 300K, 500 MHz).

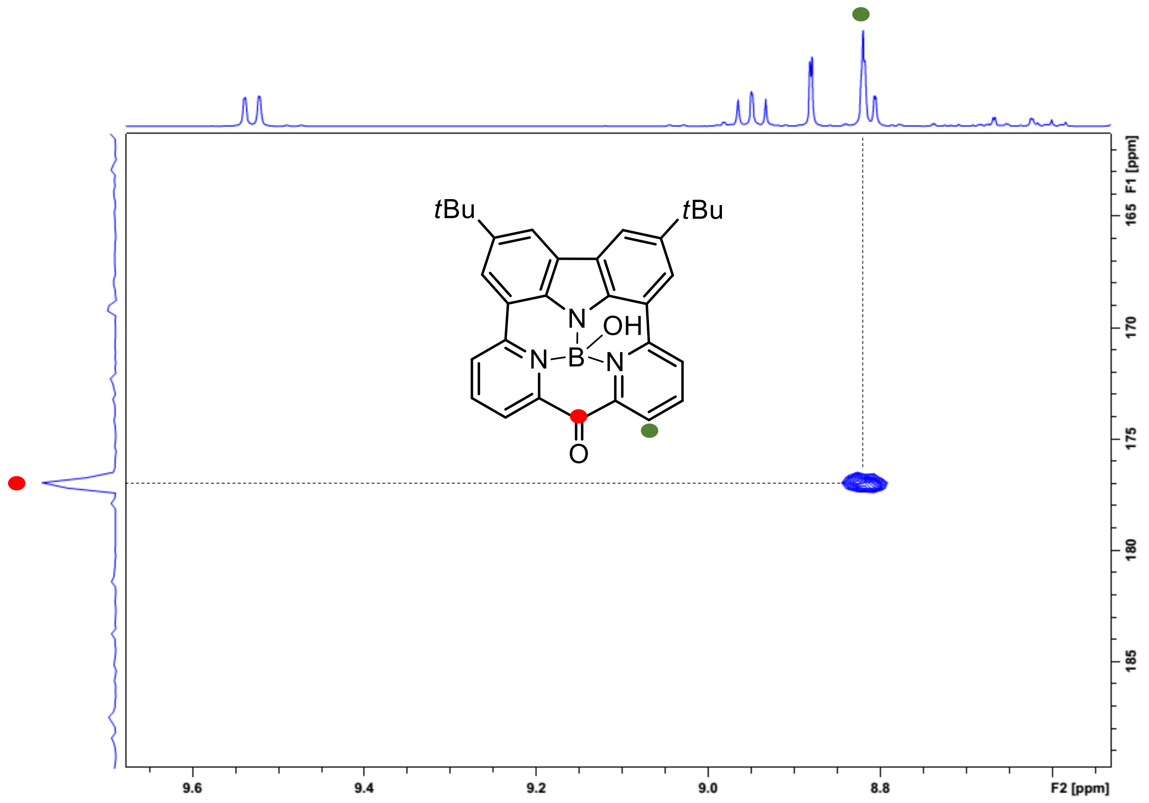

Figure S22. HMBC spectrum (zoom, aromatic region) of **4a** ($(CD_3)CO$, 300K, 500 MHz).

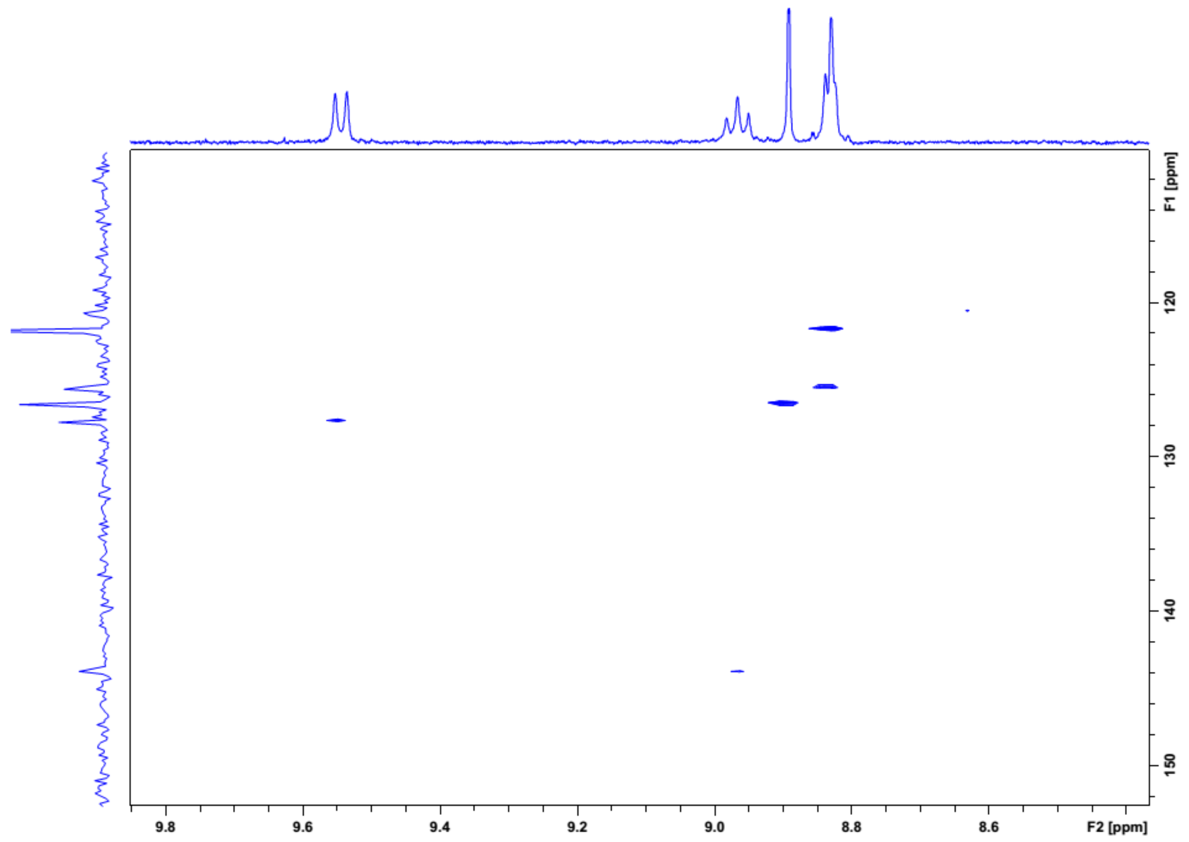

Figure S23. HSQC spectrum (zoom, aromatic region) of **4a** ($(CD_3)CO$, 300K, 500 MHz).

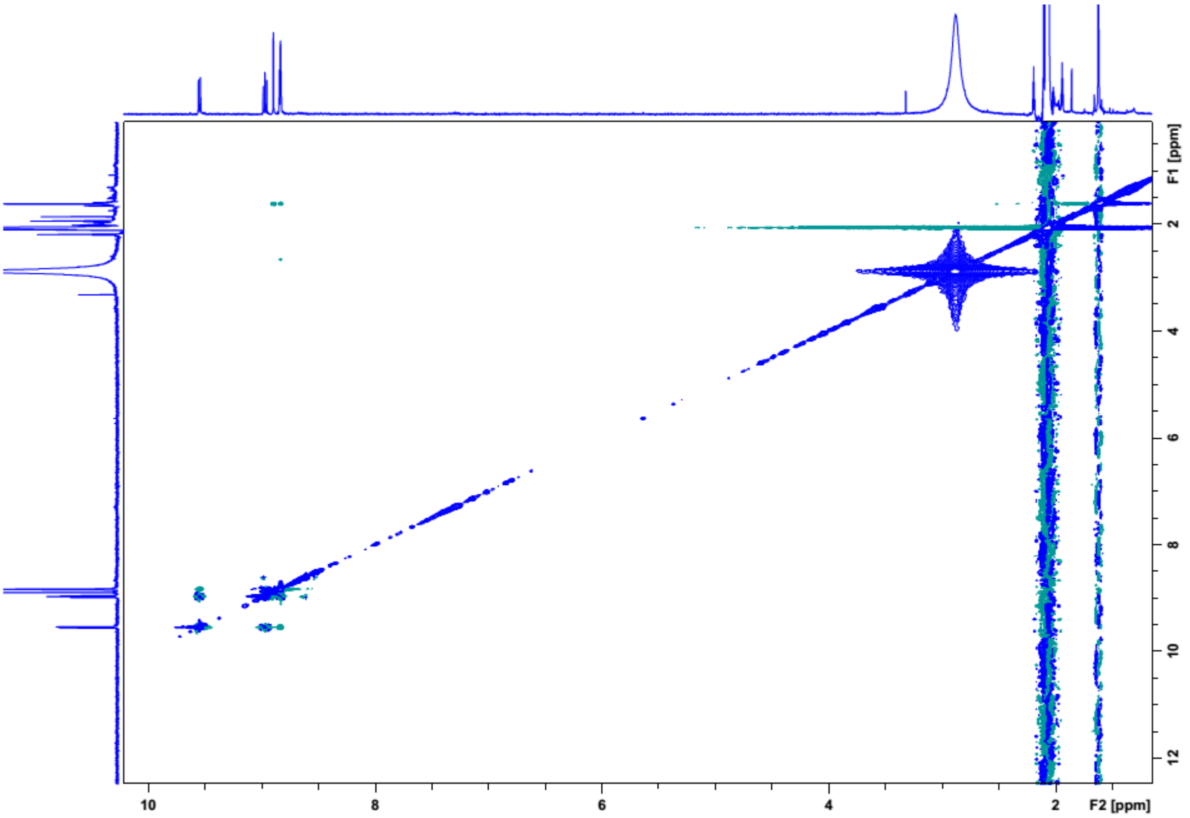

Figure S24. NOESY spectrum of **4a** ($(CD_3)CO$, 300K, 500 MHz).

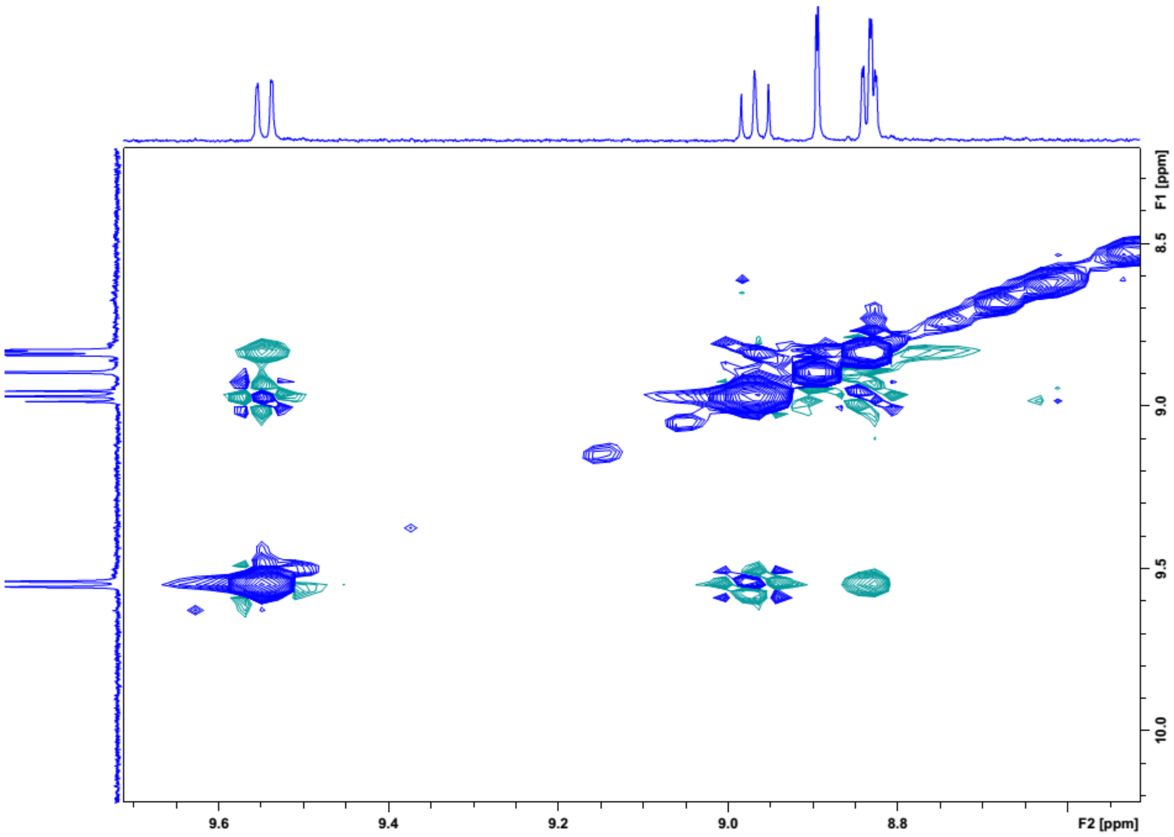

Figure S25. NOESY spectrum (zoom, aromatic region) of **4a** ($(CD_3)CO$, 300K, 500 MHz).

3.4 NMR spectra of 4b

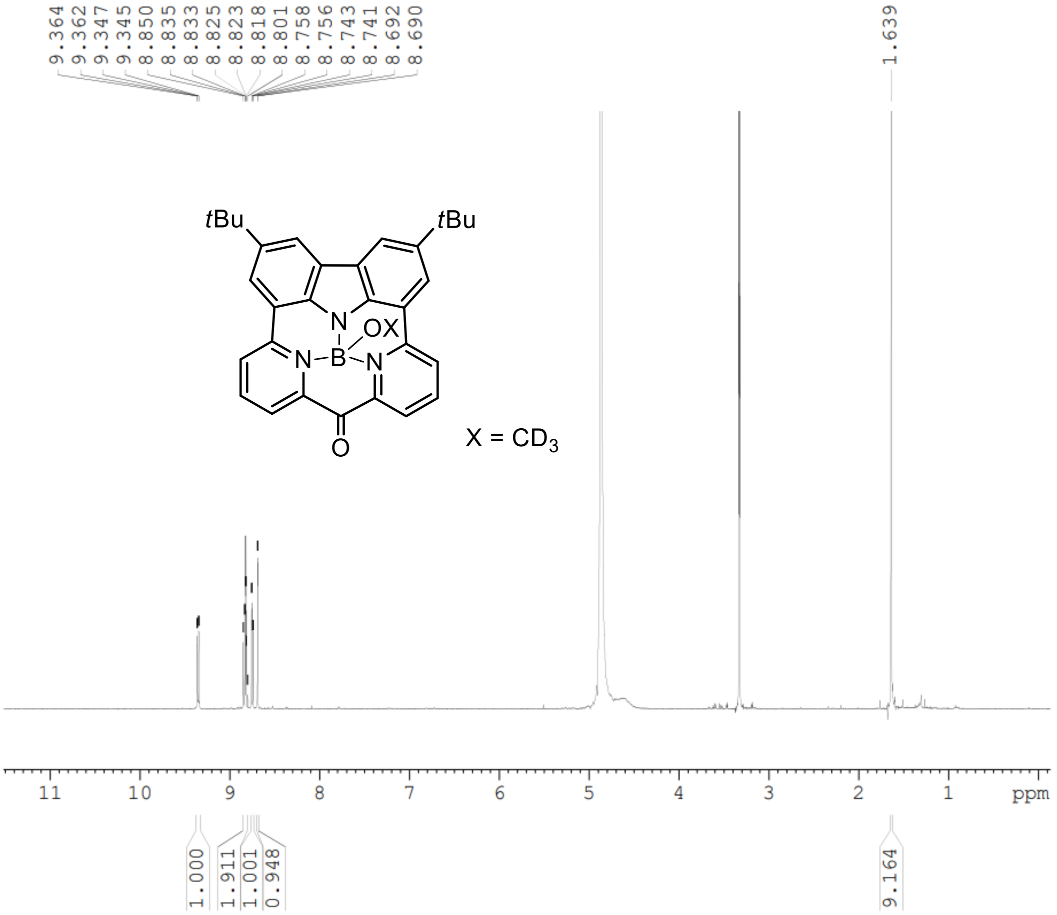

Figure S26. ¹H NMR spectrum of **4b** (CD_3OD, 300K, 500 MHz).

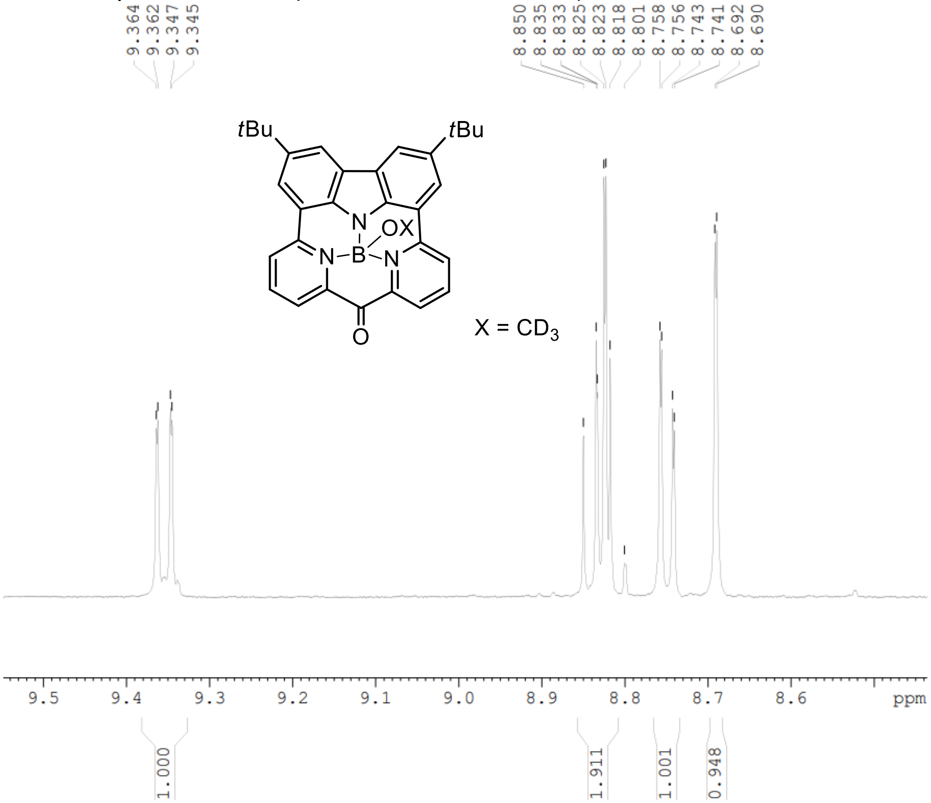

Figure S27. ¹H NMR spectrum (zoom, aromatic region) of 4b (CD_3OD, 300K, 600 MHz).

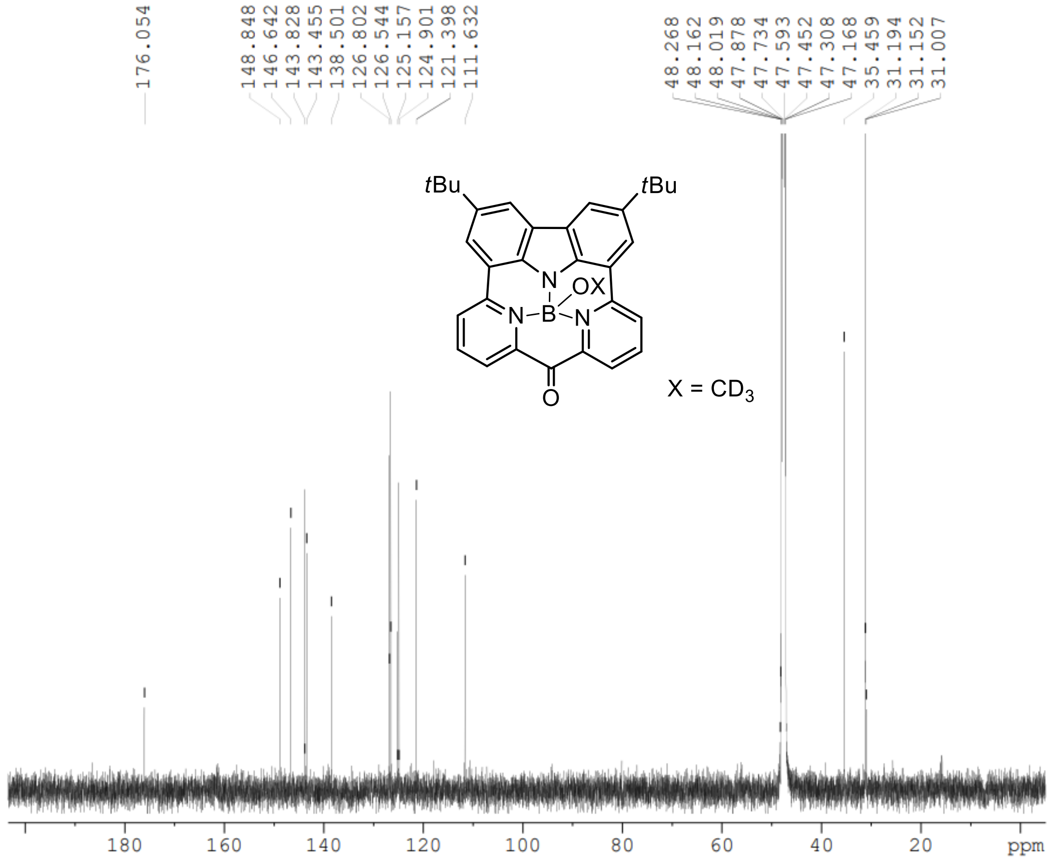

Figure S28. ^{13}C spectrum of **4b** (CD_3OD, 300K, 150 MHz).

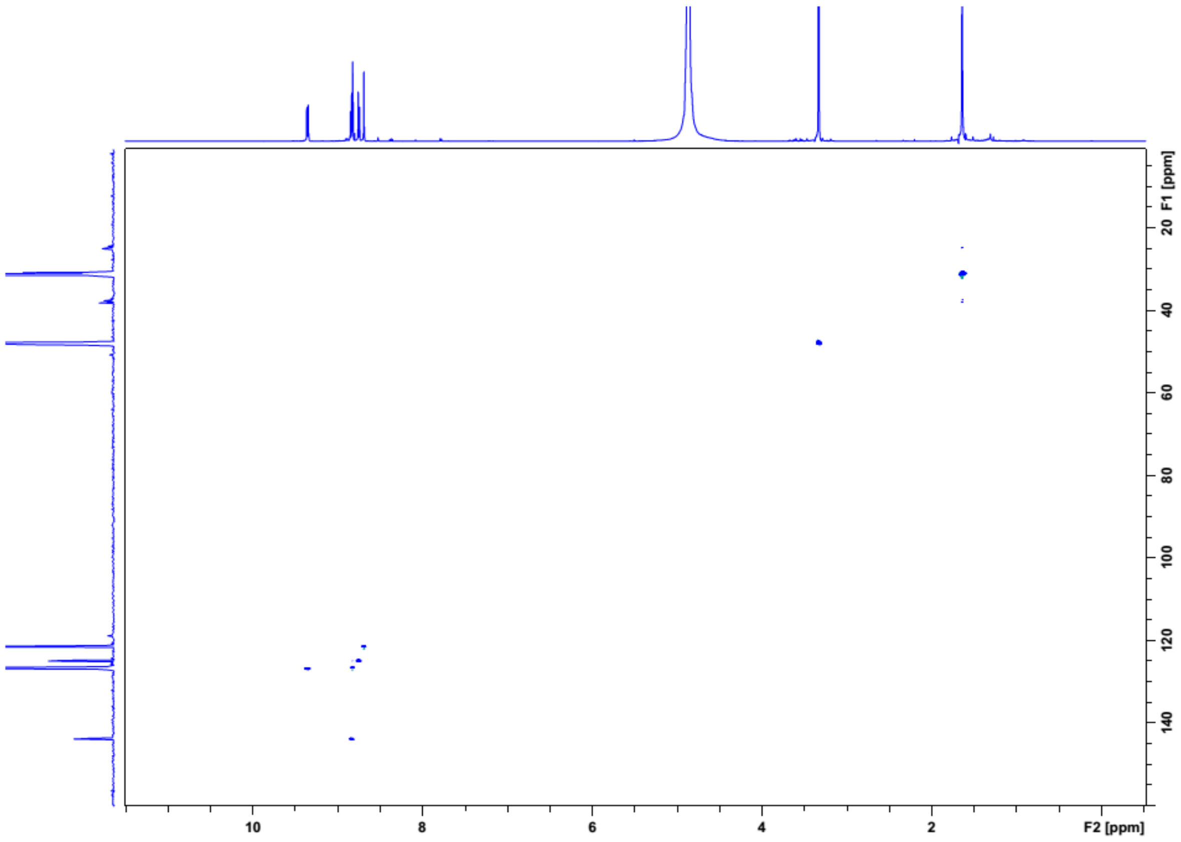

Figure S29. HSQC spectrum of **4b** (CD_3OD, 300K, 600 MHz).

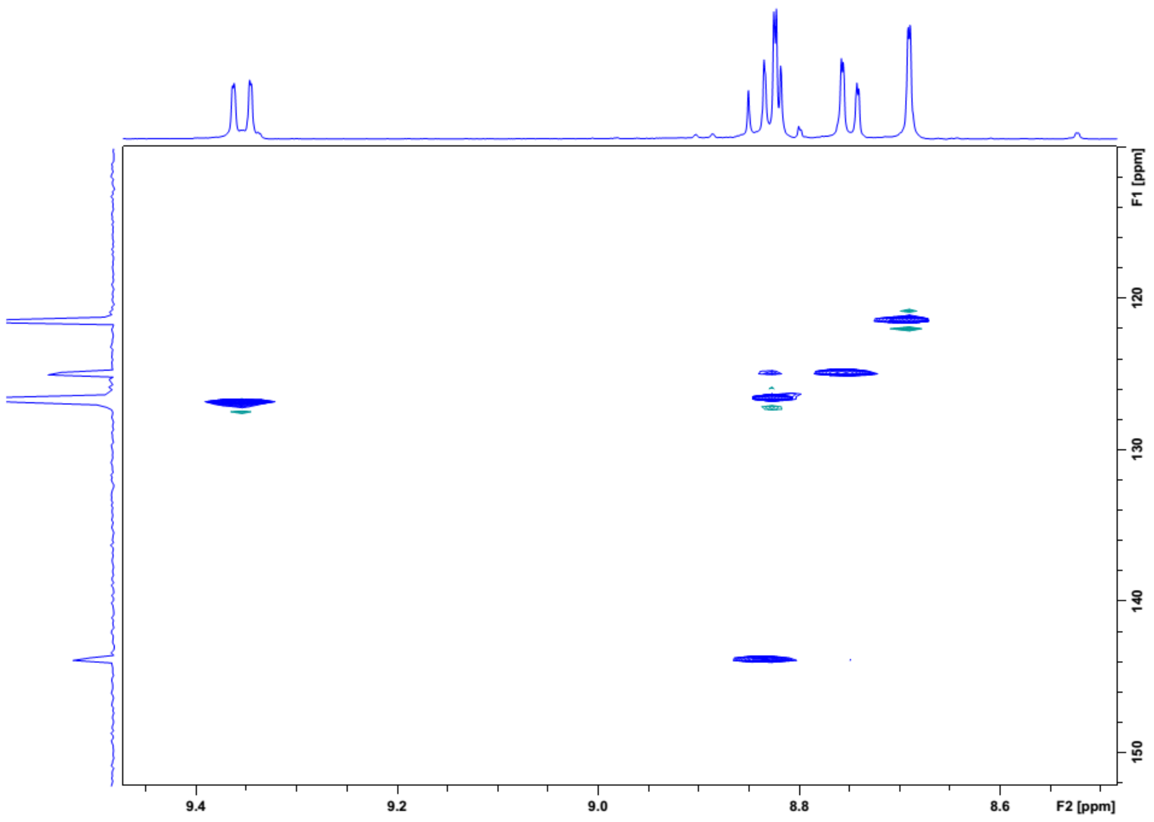

Figure S30. HSQC spectrum (zoom, aromatic region) of **4b** (CD_3OD, 300K, 600 MHz).

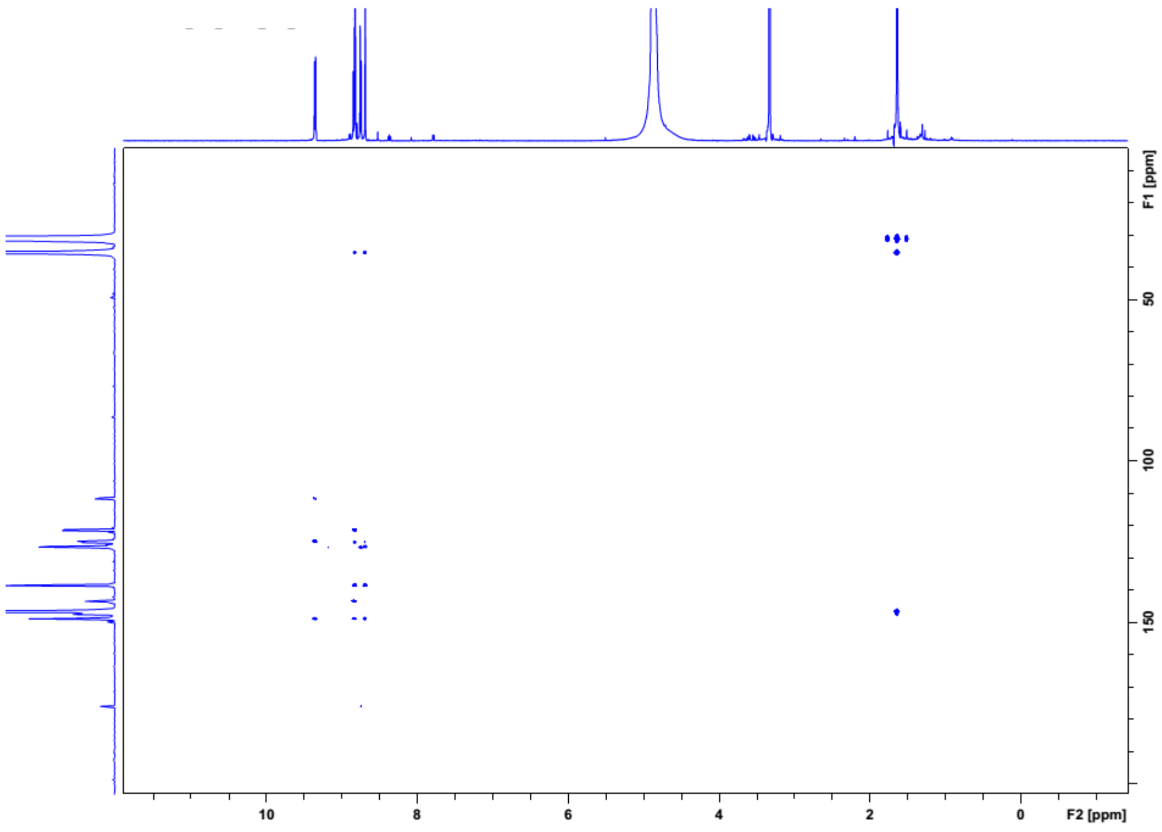

Figure S31. HMBC spectrum of **4b** (CD_3OD, 300K, 600 MHz).

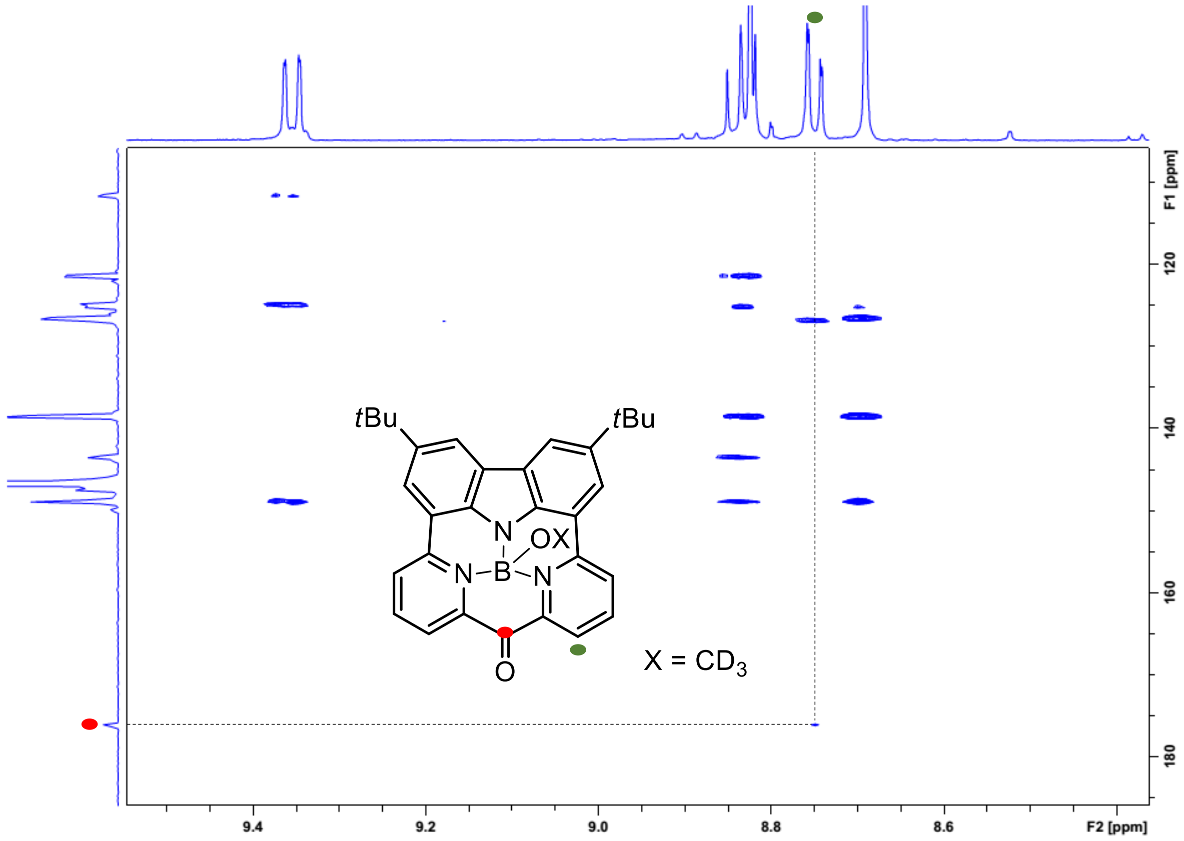

Figure S32. HMBC spectrum (zoom, aromatic region) of **4b** (CD_3OD, 300K, 600 MHz).

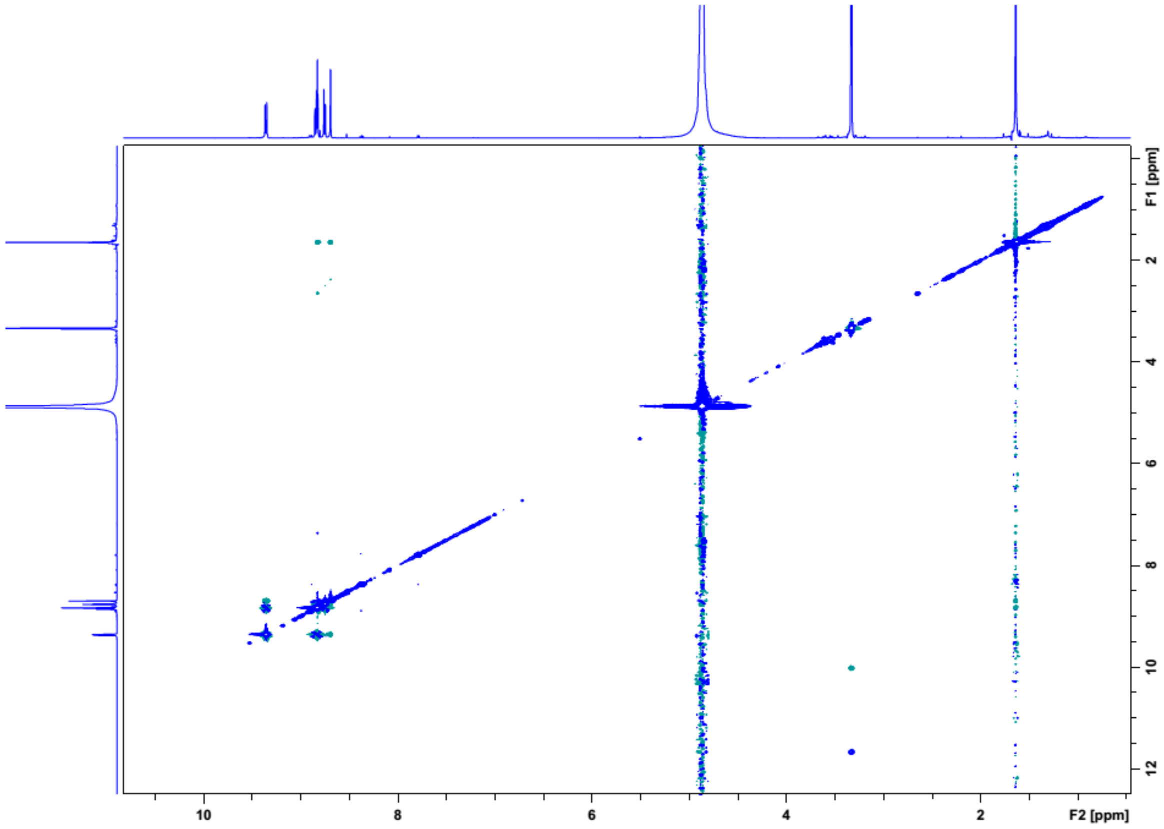

Figure S33. NOESY spectrum of **4b** (CD_3OD, 300K, 600 MHz).

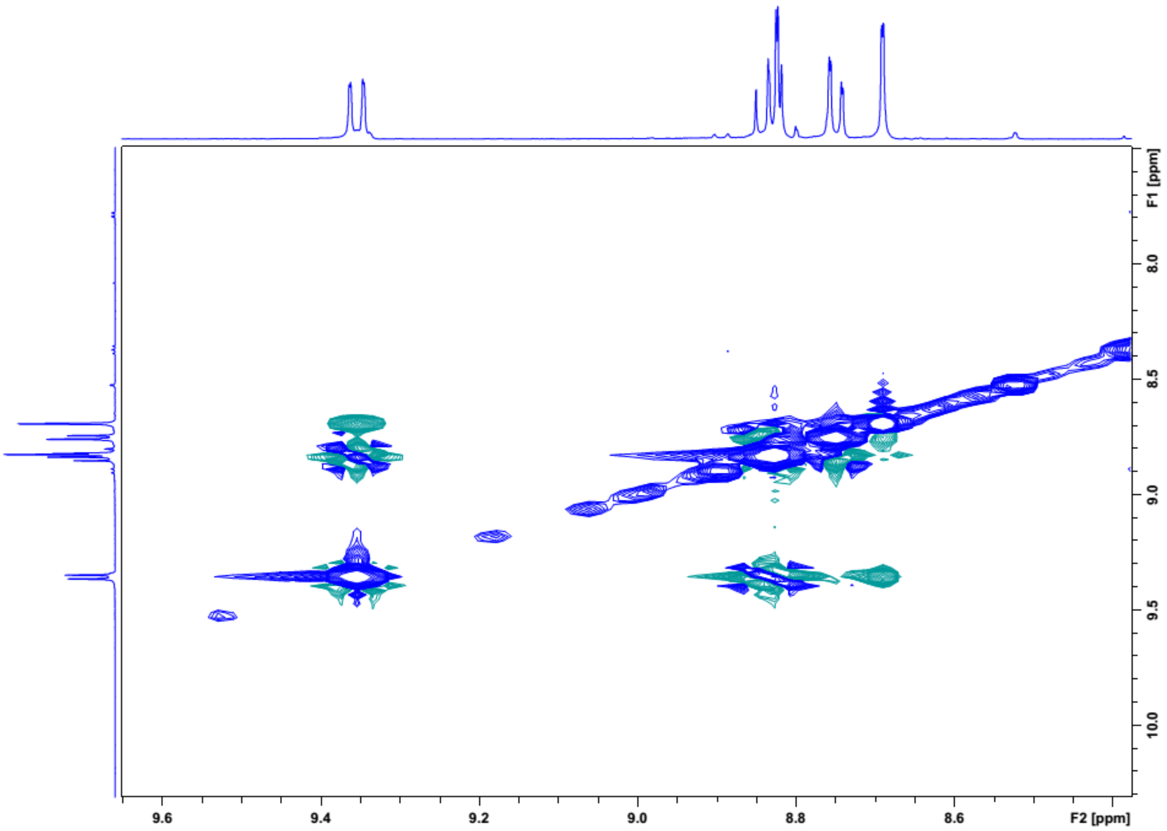

Figure S34. NOESY spectrum (zoom, aromatic region) of **4b** (CD_3OD, 300K, 600 MHz).

3.5 NMR spectra of 4-Ac

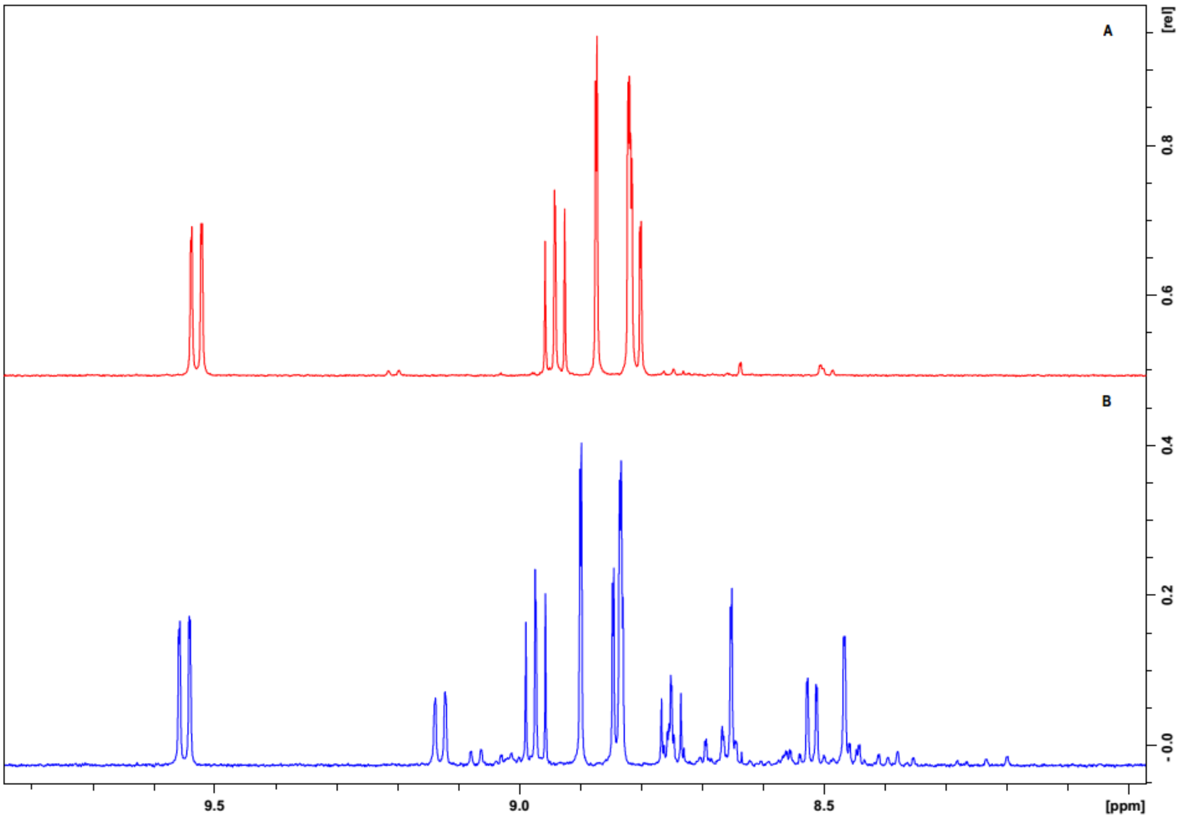

Figure S35. Comparison of ^{1}H NMR spectra of **4a** (panel A) and **4-Ac** (panel B) (($(CD_3)CO$, 300K, 500 MHz).

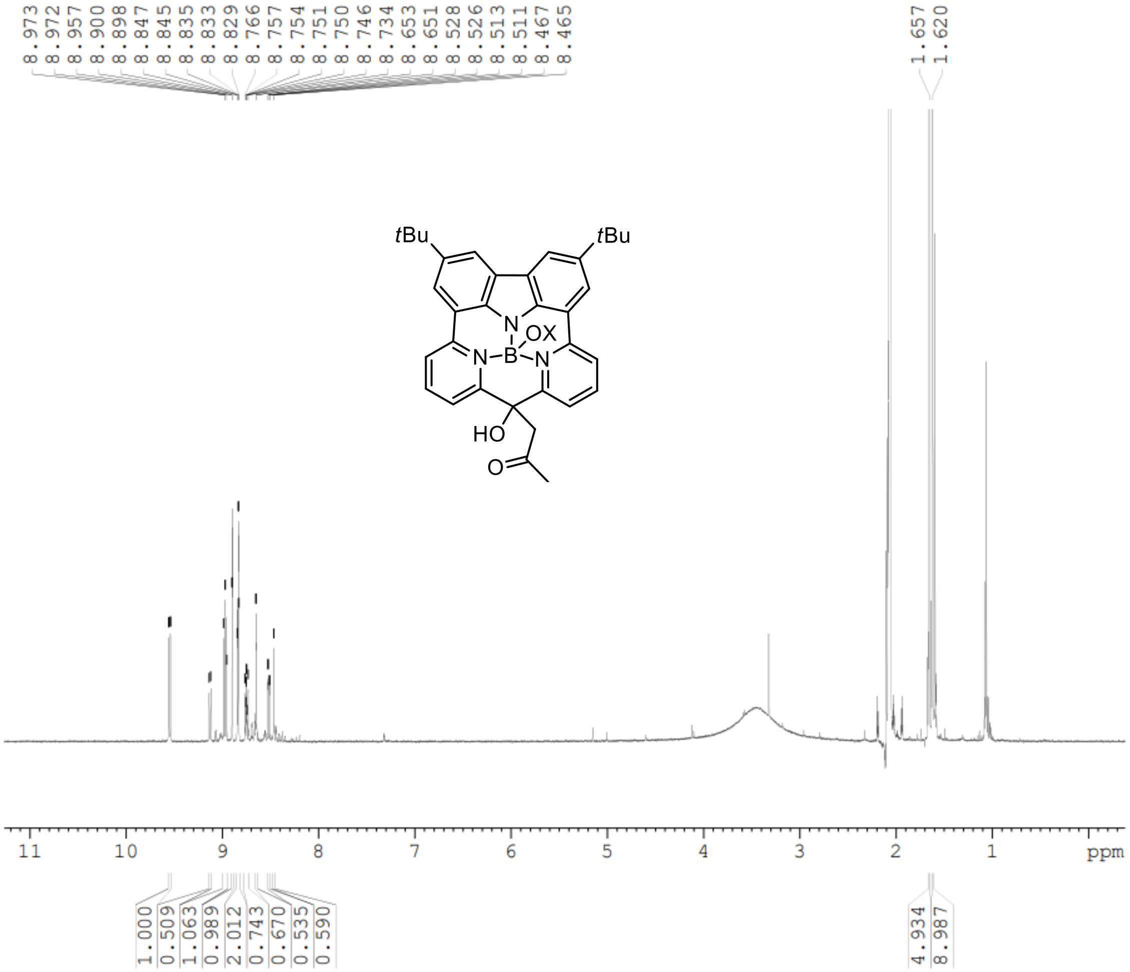

Figure S36. ^{1}H NMR spectrum of **4-Ac** ($(CD_3)CO$, 300K, 500 MHz).

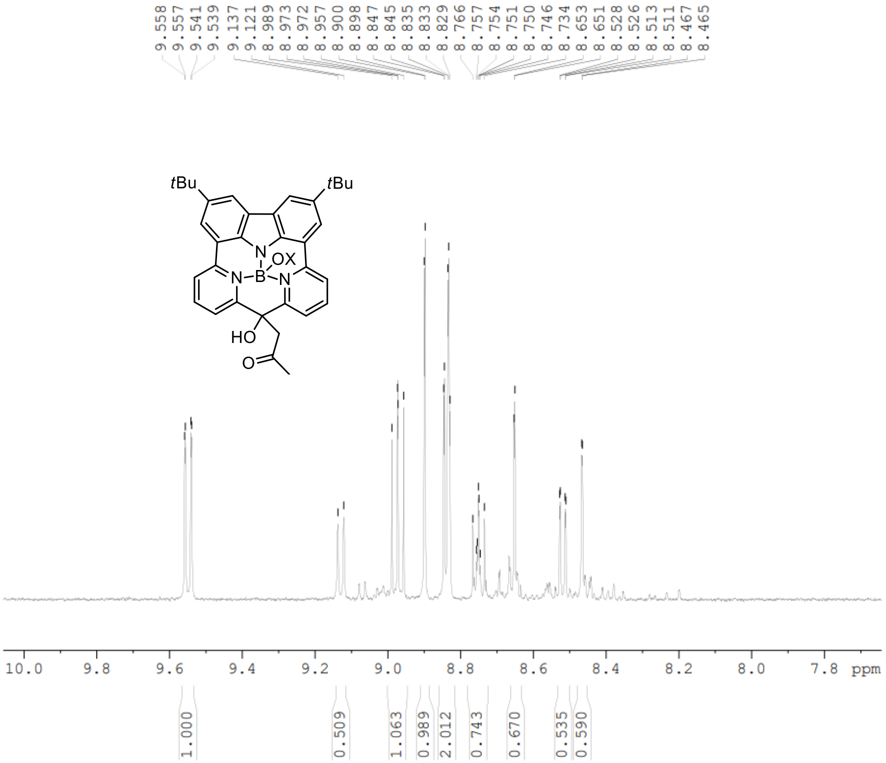

Figure S37. ^{1}H NMR spectrum (zoom, aromatic region) of **4-Ac** ($(CD_3)CO$, 300K, 500 MHz).

3.6 NMR spectra of 5 and 5-Ac

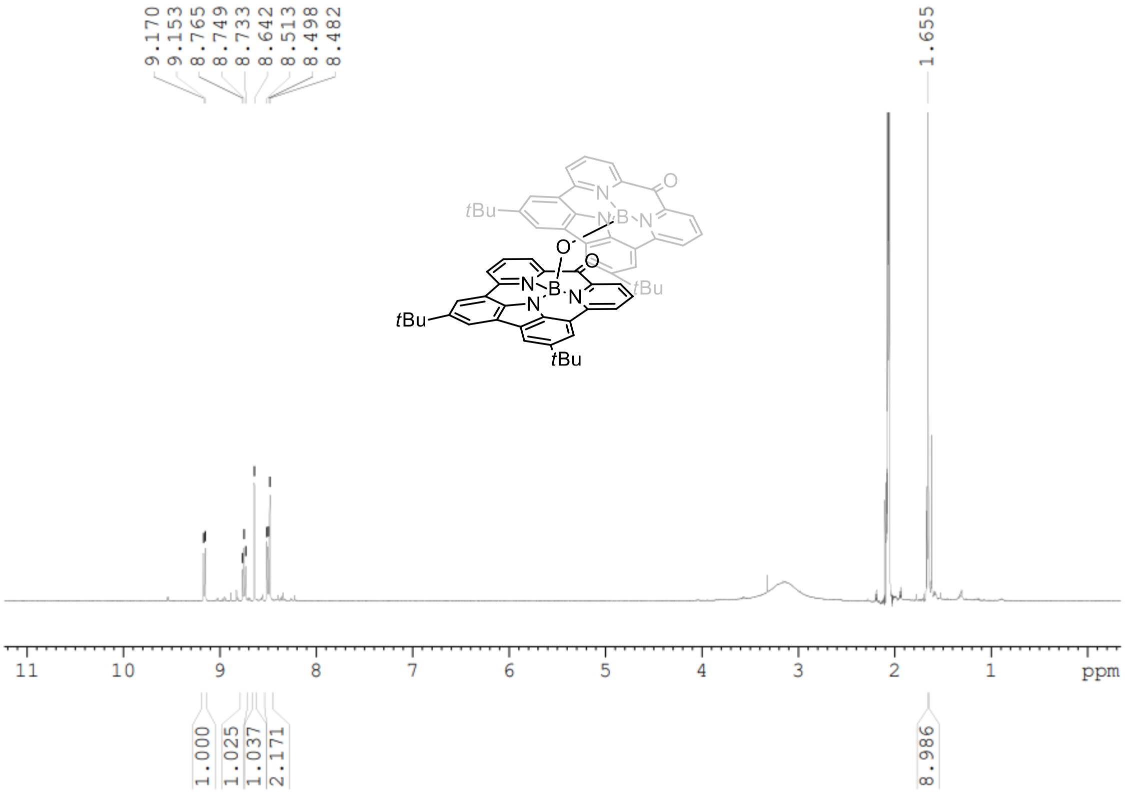

Figure S38. ^{1}H spectrum of **5** ($(CD_3)_2CO$, 300K, 600 MHz).

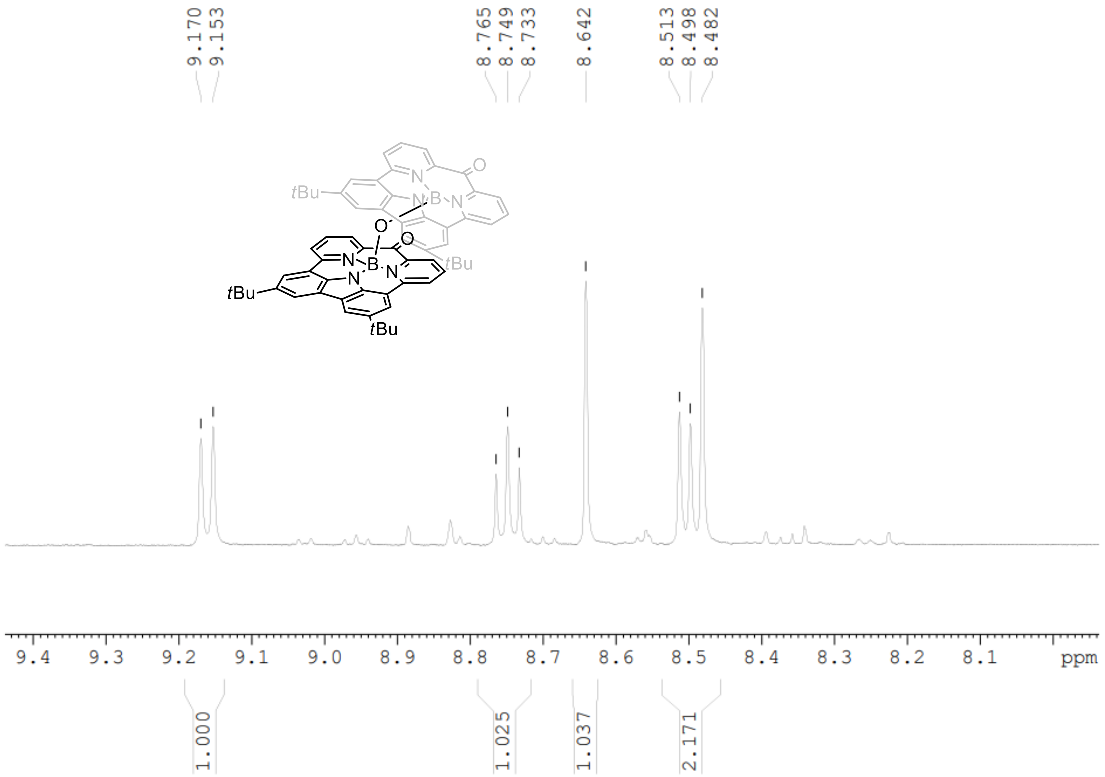

Figure S39. ^{1}H spectrum of **5** (zoom, aromatic region) ($(CD_3)_2CO$, 300K, 600 MHz).

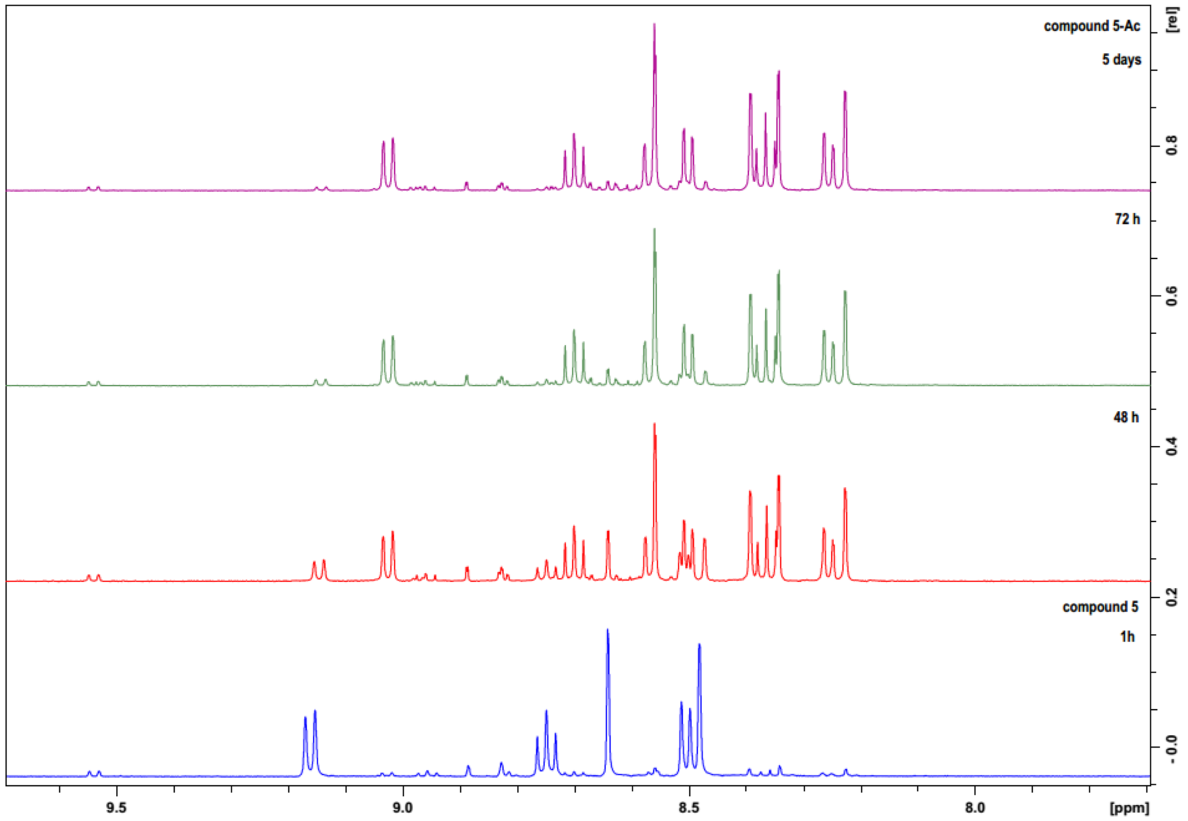

Figure S40. Stability of compound **5** (zoom, aromatic region) ($(CD_3)_2CO$, 300K, 600 MHz).

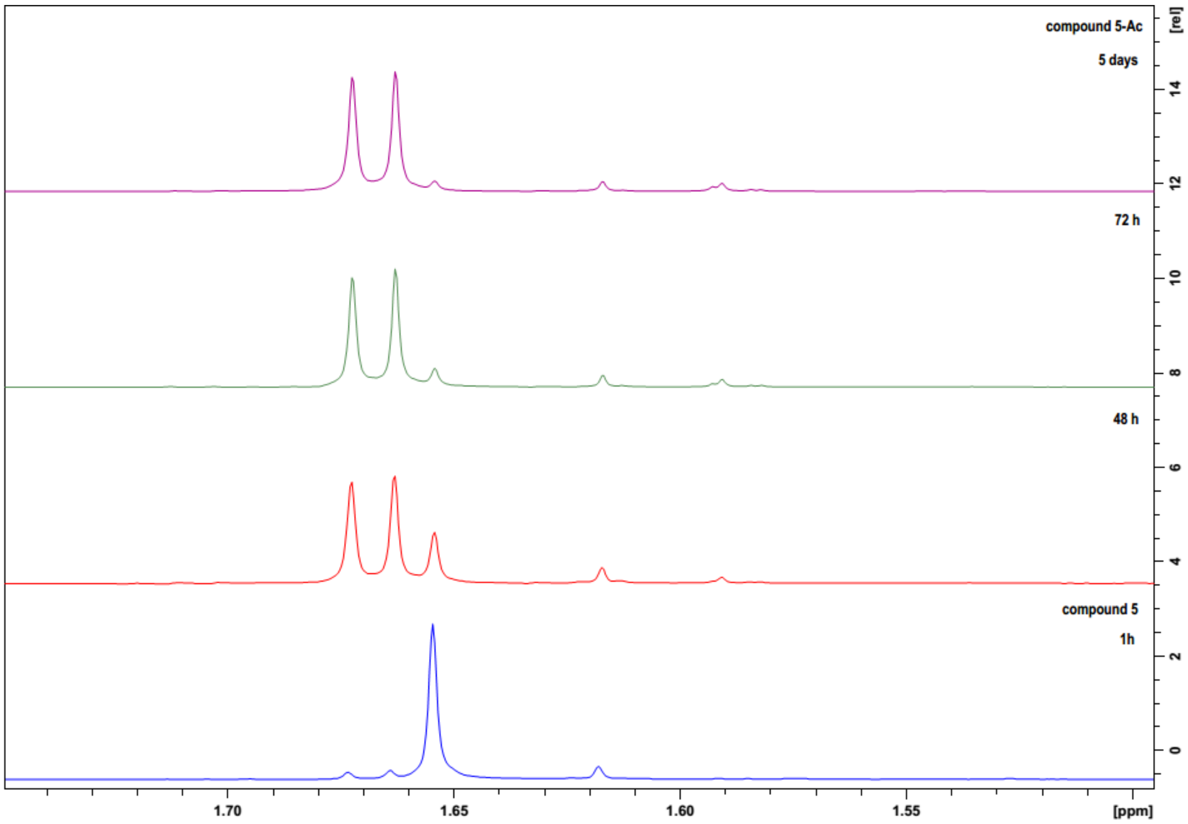

Figure S41. Stability of compound **5** (zoom, aliphatic region) ($(CD_3)_2CO$, 300K, 600 MHz).

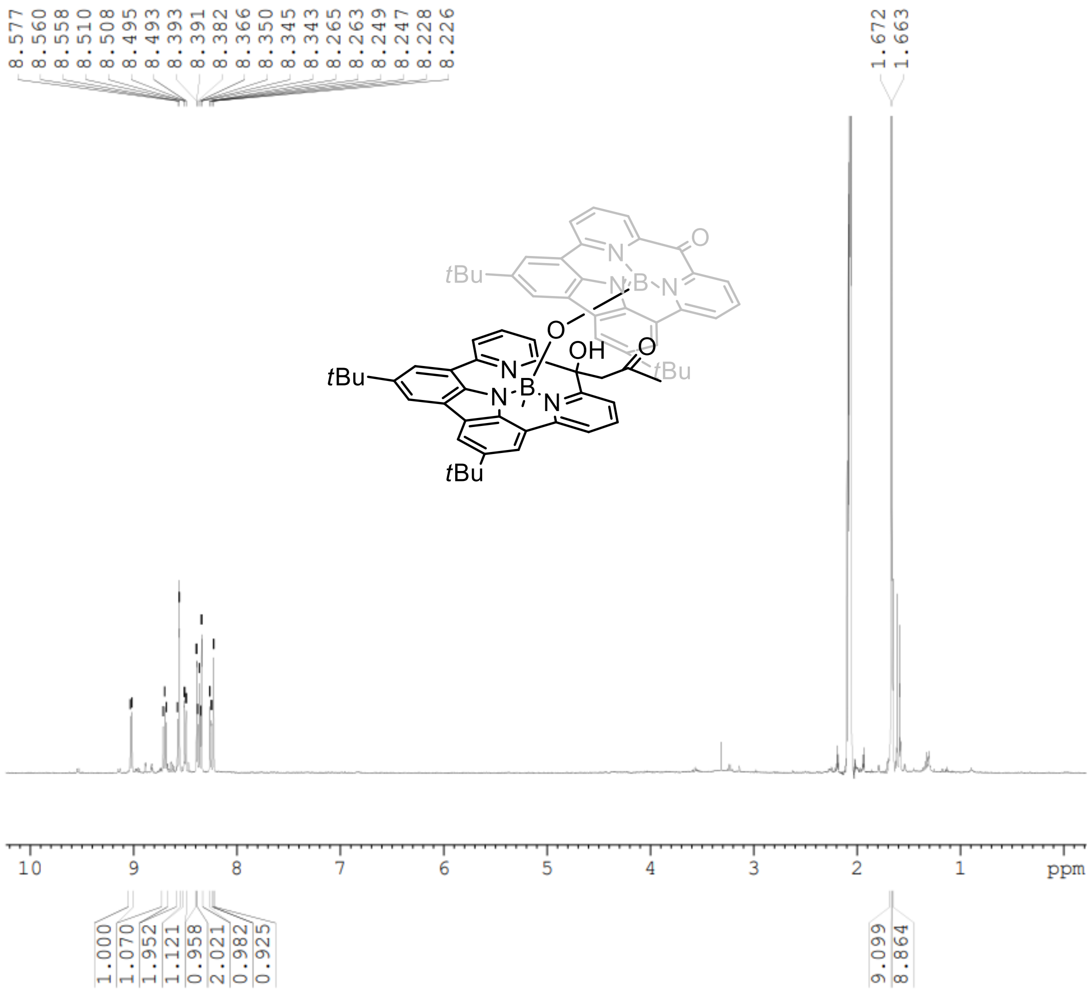

Figure S42. ^{1}H spectrum of **5-Ac** ($(CD_3)_2CO$, 300K, 600 MHz).

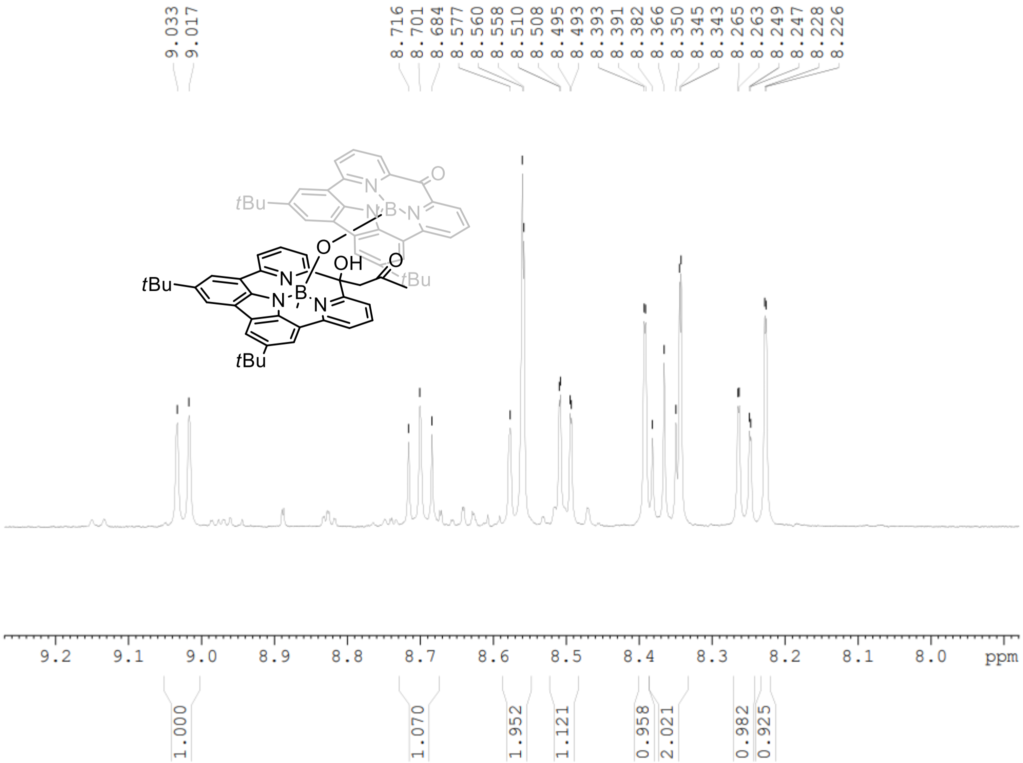

Figure S43. ^{1}H spectrum (zoom, aromatic region) of 5-Ac ($(CD_3)_2CO$, 300K, 600 MHz).

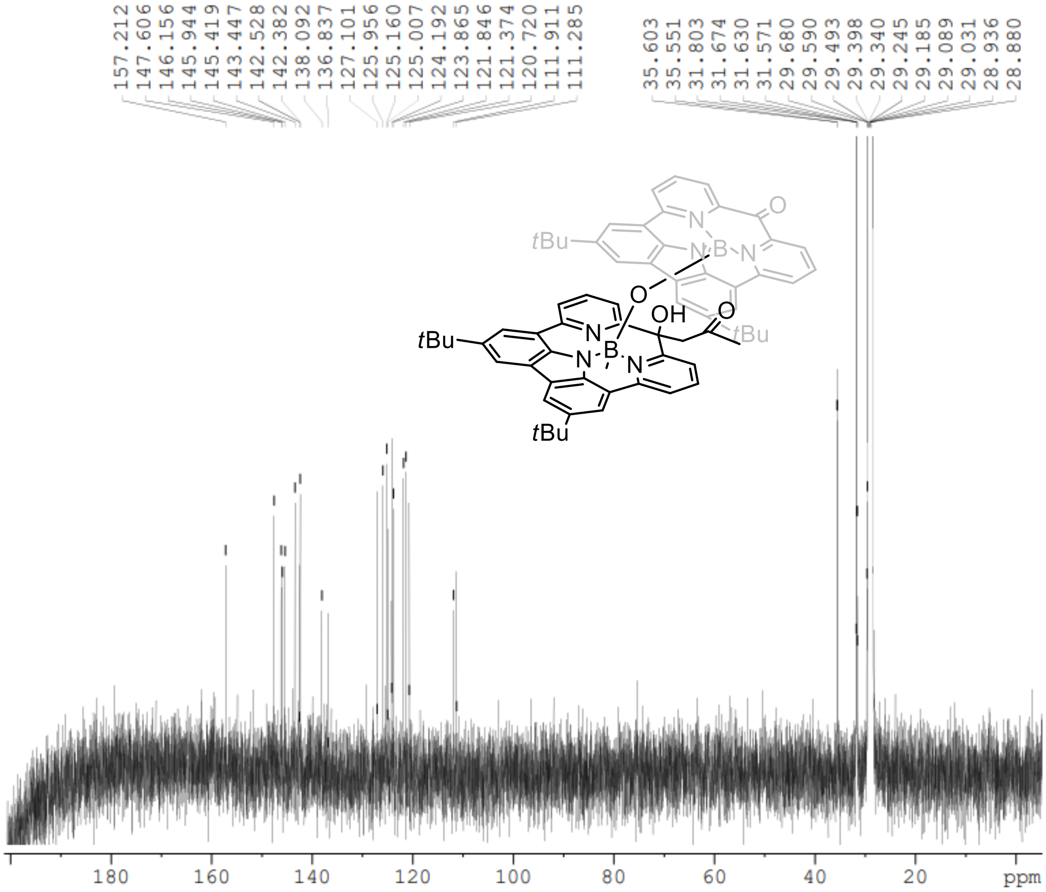

Figure S44. ^{13}C spectrum of 5-Ac ($(CD_3)_2CO$, 300K, 150 MHz).

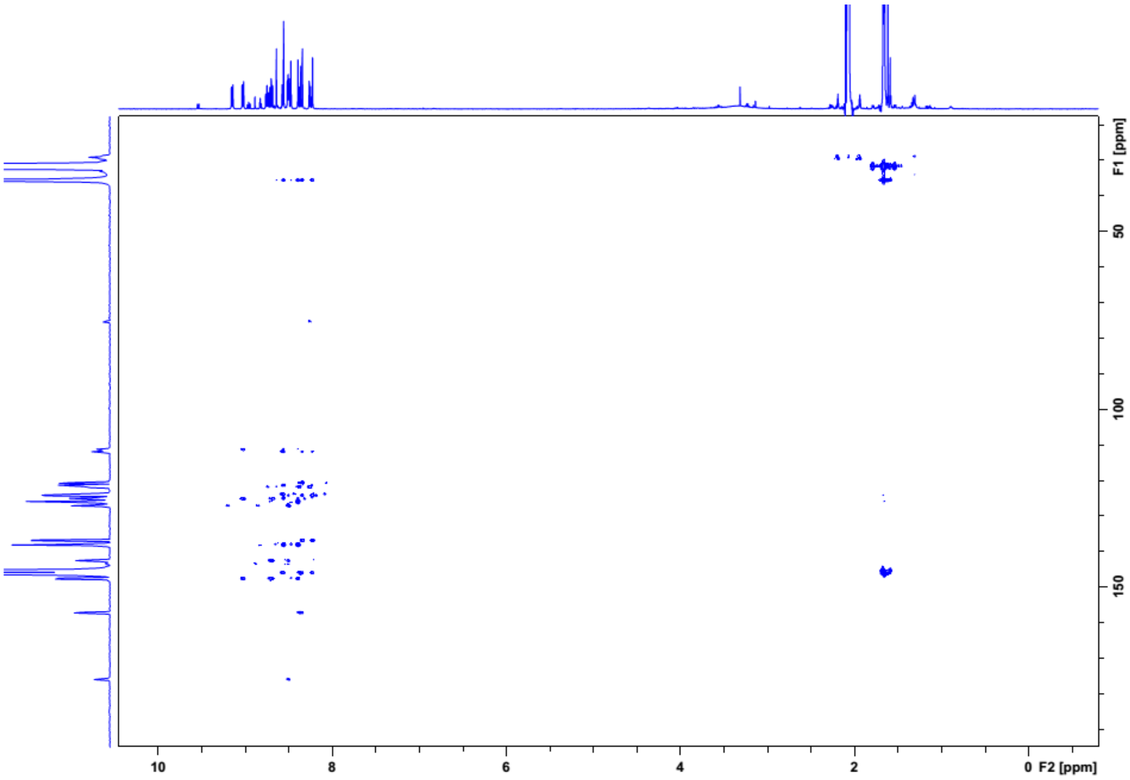

Figure S45. HMBC spectrum of **5-Ac** ($(CD_3)_2CO$, 300K, 600 MHz).

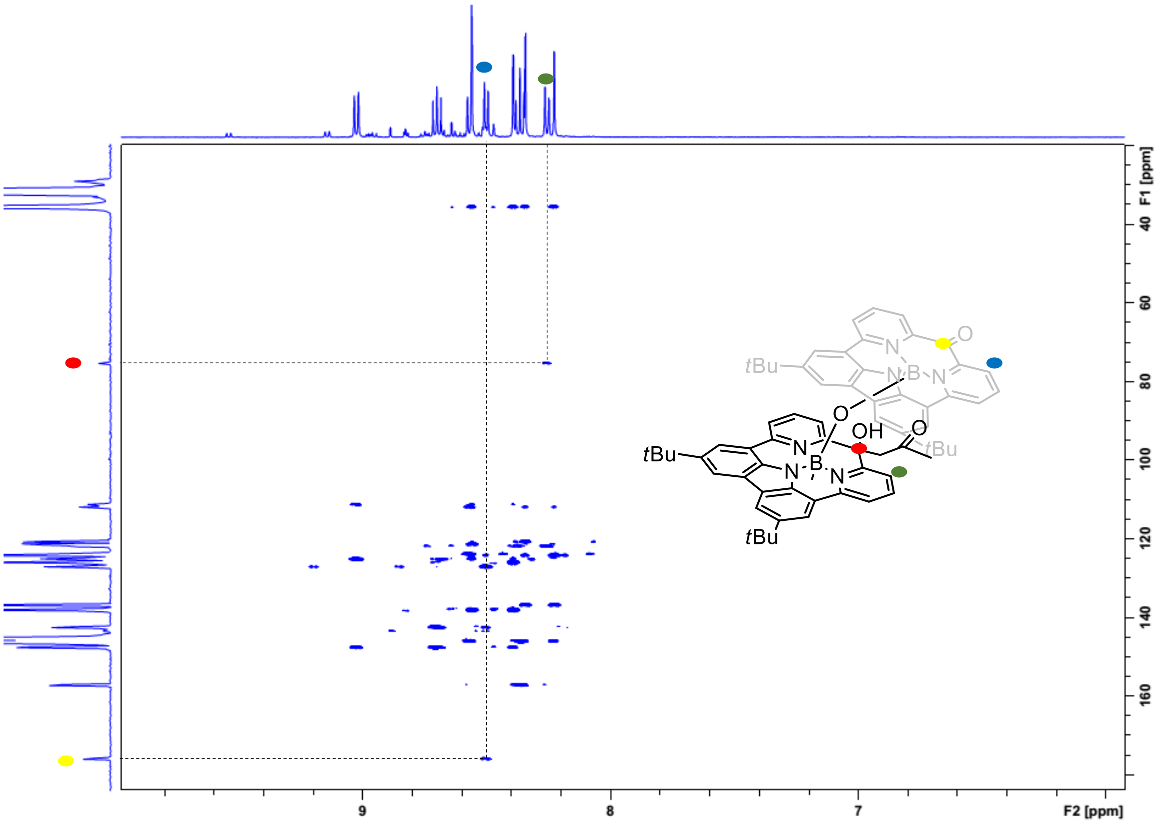

Figure S46. HMBC spectrum (zoom, aromatic region) of **5-Ac** ($(CD_3)_2CO$, 300K, 600 MHz).

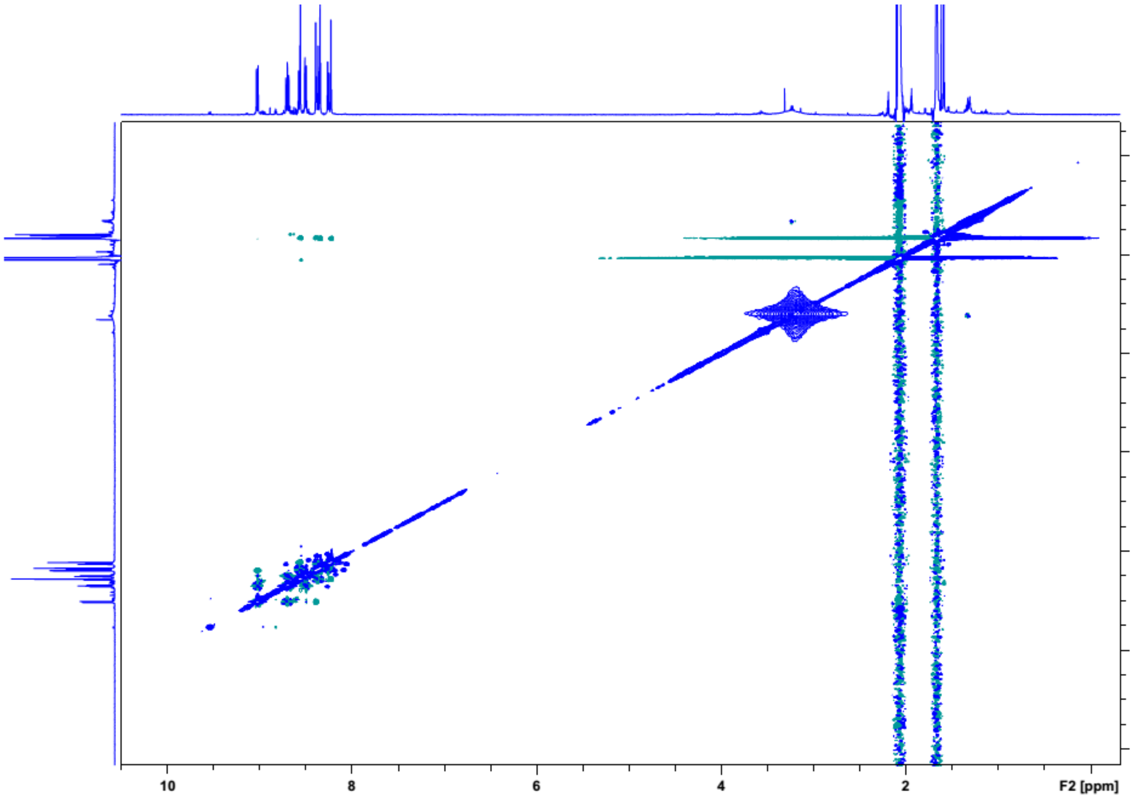

Figure S47. NOESY spectrum of **5-Ac** ($(CD_3)_2CO$, 300K, 600 MHz).

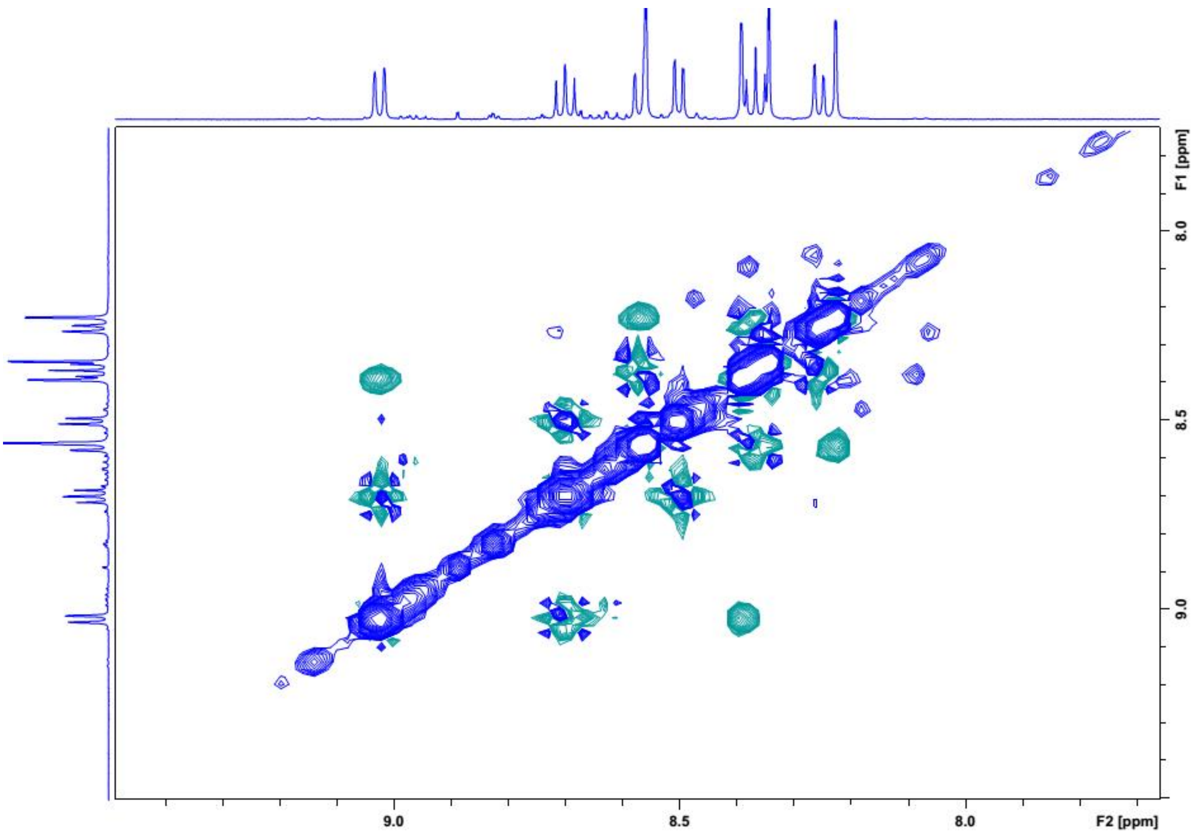

Figure S48. NOESY spectrum (zoom, aromatic region) of **5-Ac** (CD_3OD, 300K, 600 MHz).

3.7 NMR spectra of 6

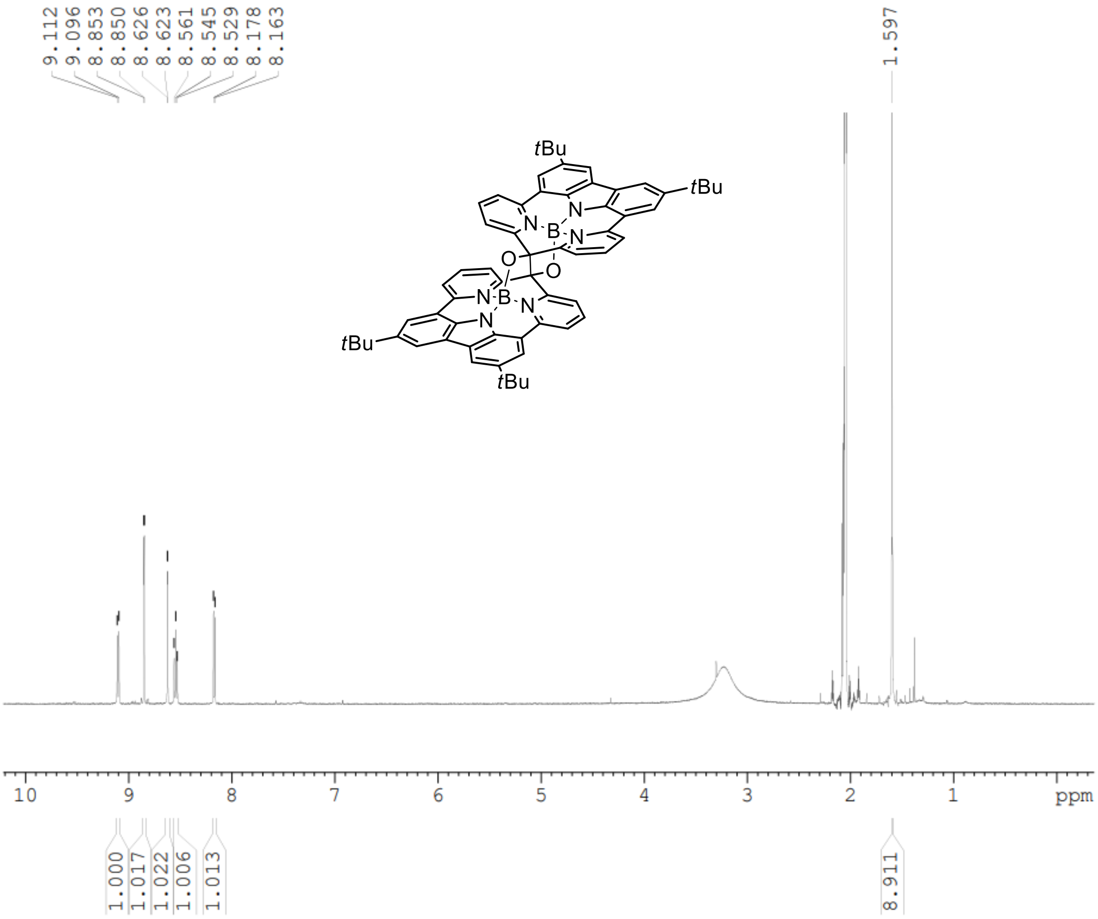

Figure S49. ^{1}H NMR spectrum of **6** ($(CD_3)CO$, 300K, 500 MHz)

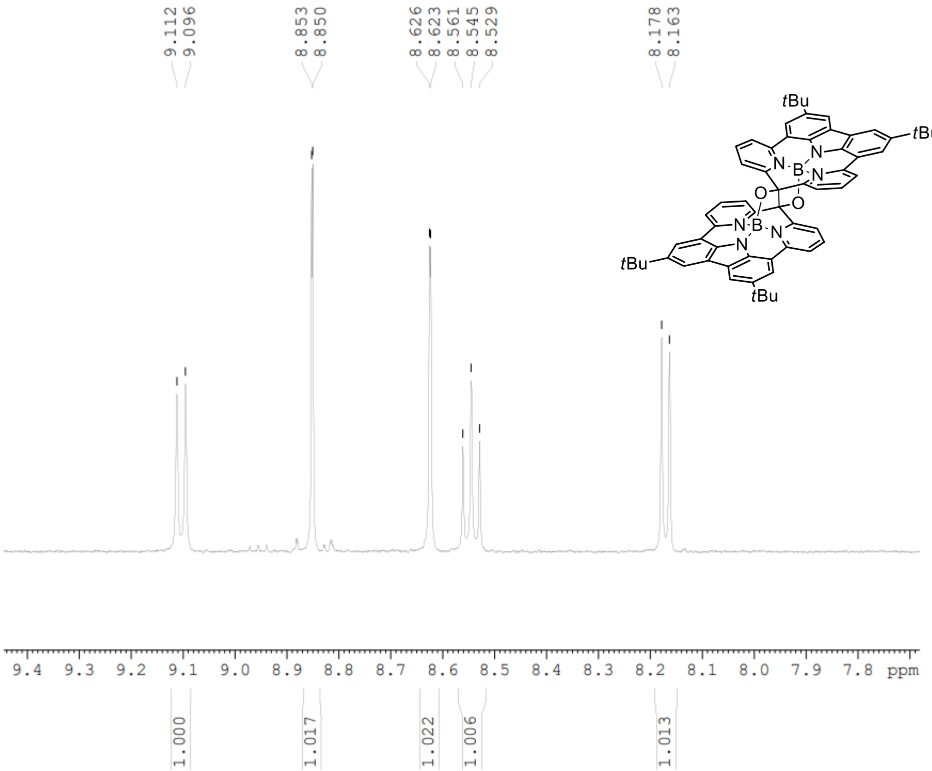

Figure S50. ^{1}H NMR spectrum (zoom, aromatic region) of **6** ($(CD_3)CO$, 300K, 500 MHz).

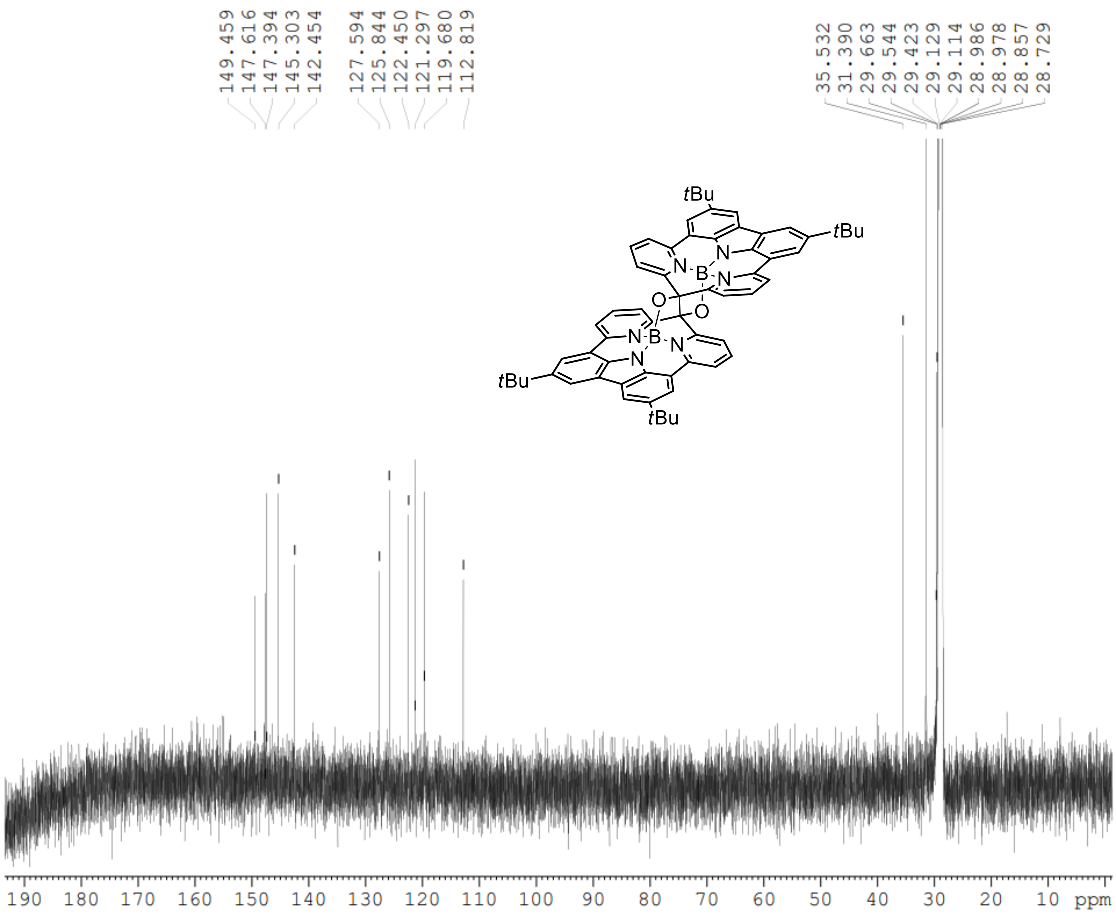

Figure S51. ^{13}C NMR spectrum of **6** ($(CD_3)CO$, 300K, 150 MHz)

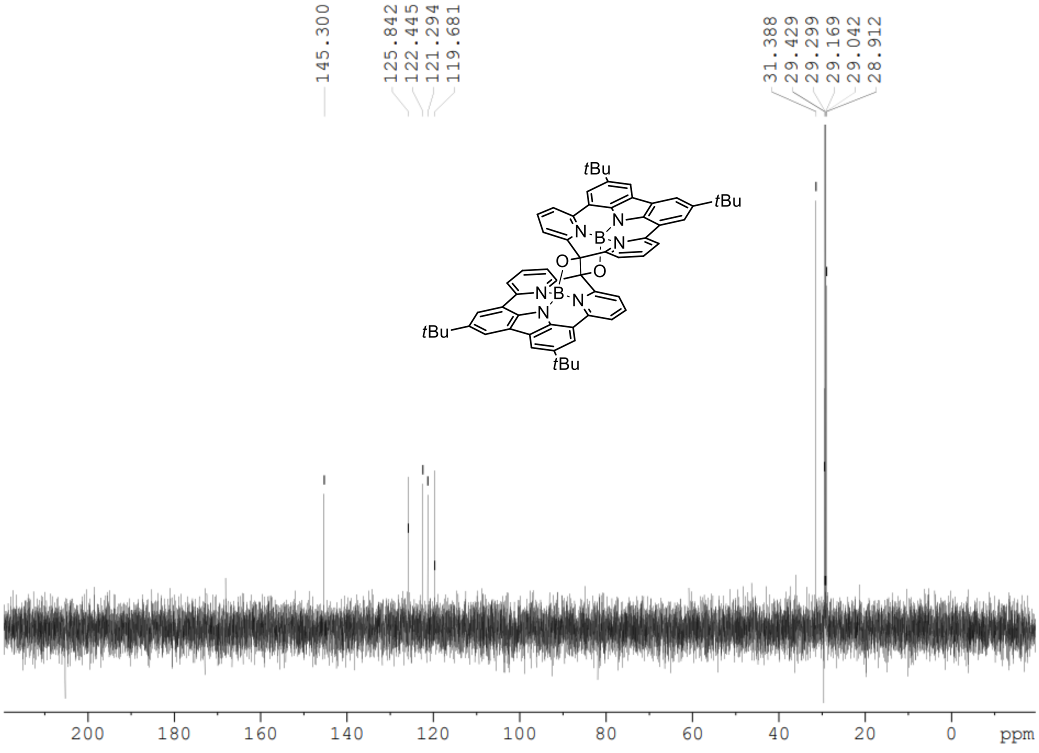

Figure S52. DEPT135 NMR spectrum of **6** ($(CD_3)CO$, 300K, 150 MHz)

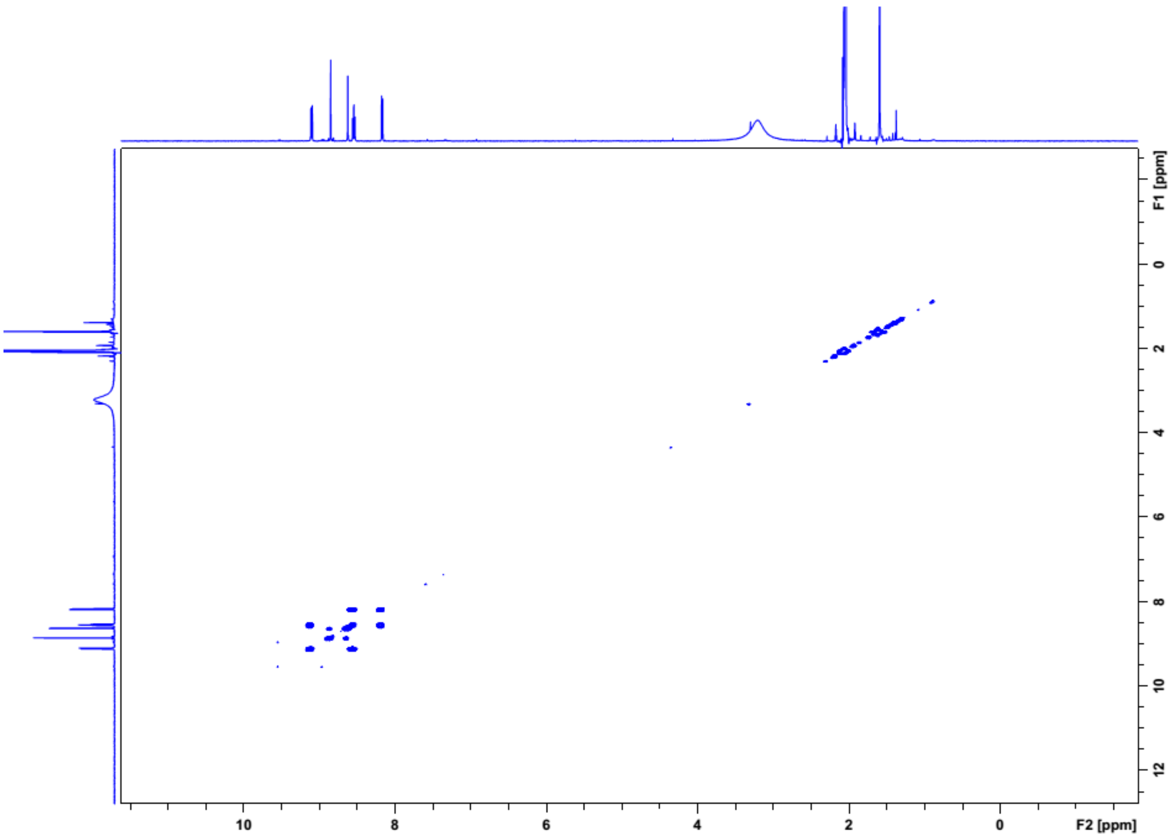

Figure S53. COSY NMR spectrum of **6** ($(CD_3)CO$, 300K, 500 MHz).

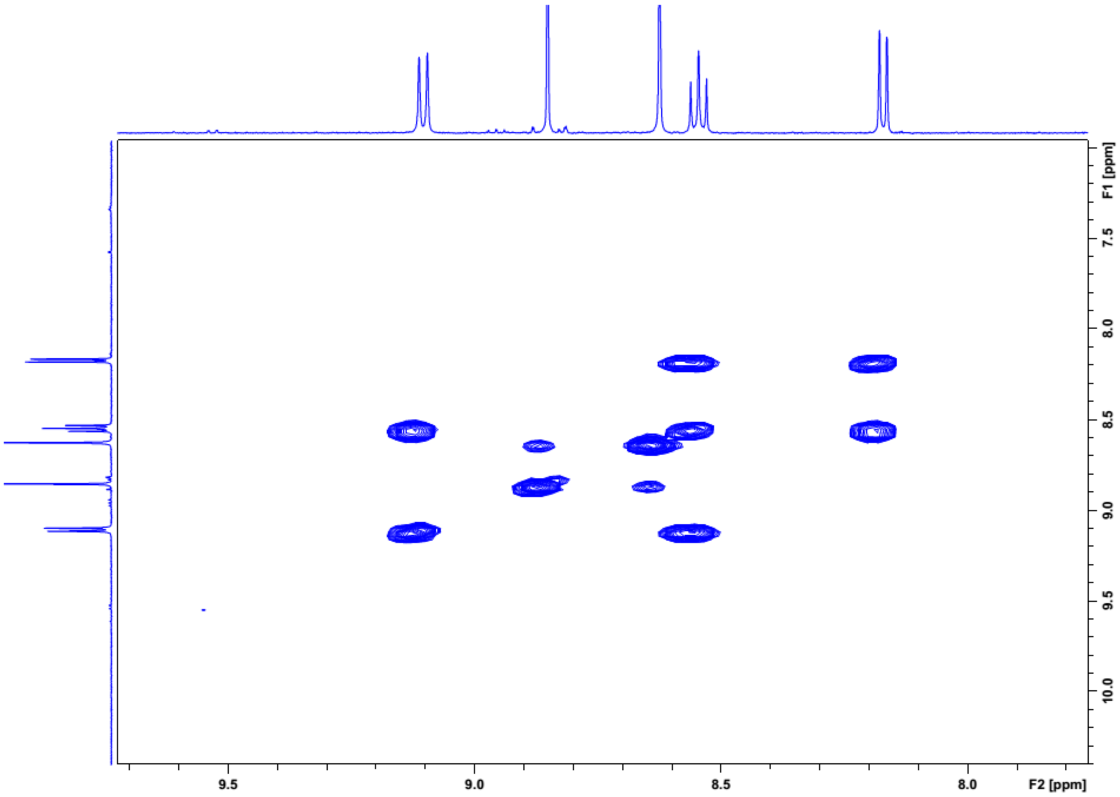

Figure S54. COSY NMR spectrum (zoom, aromatic region) of **6** (($(CD_3)CO$, 300K, 500 MHz).

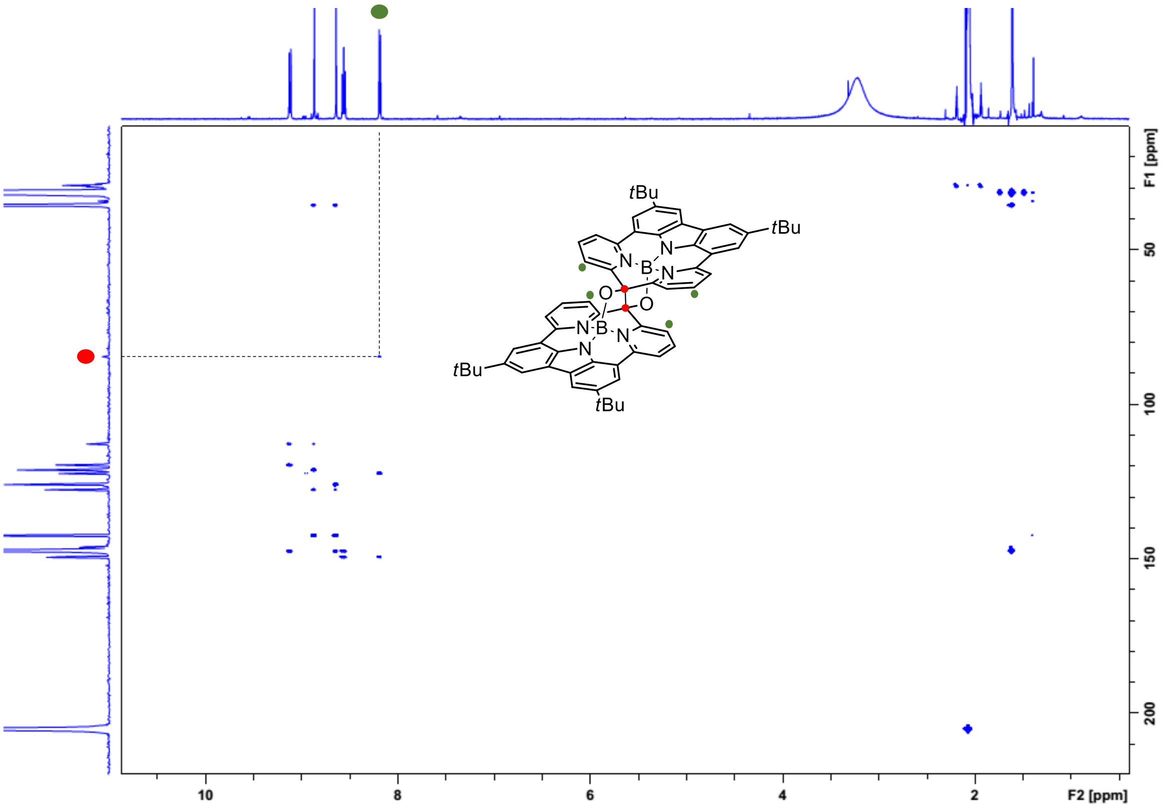

Figure S55. HMBC NMR spectrum of **6** (($(CD_3)CO$, 300K, 500 MHz).

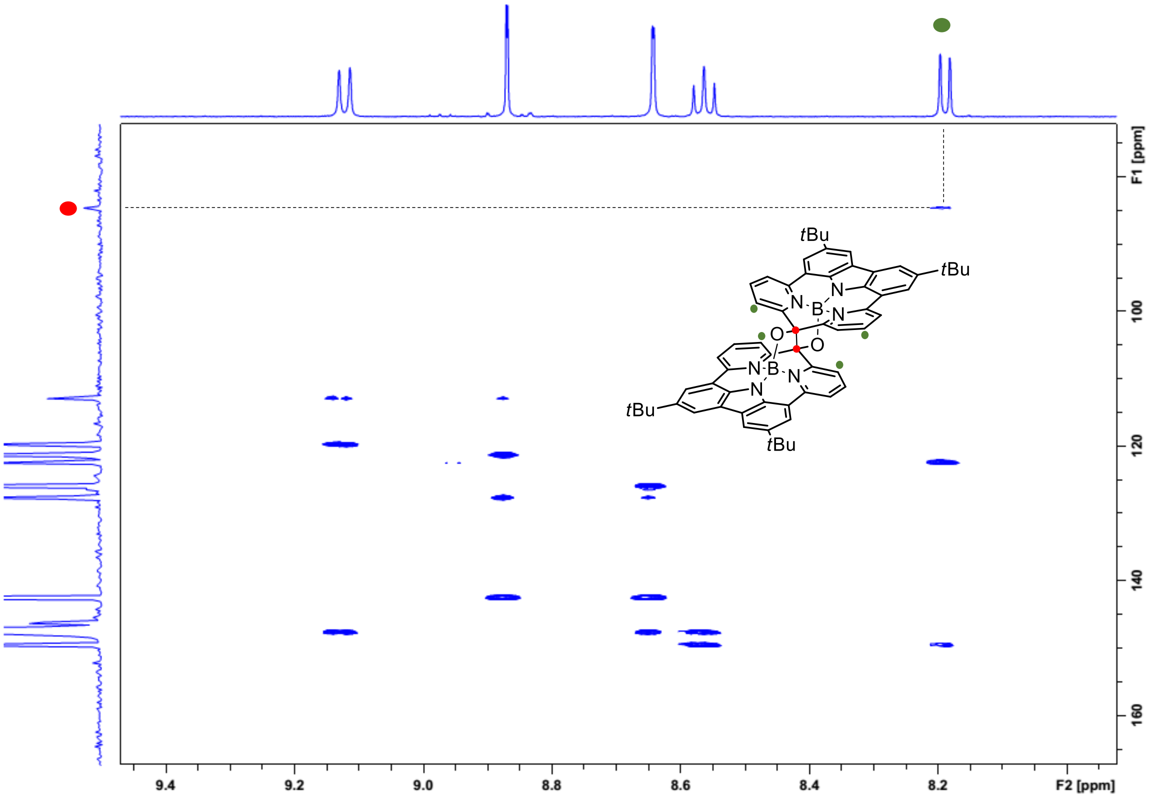

Figure S56. HMBC NMR spectrum (zoom, aromatic region) of **6** (($(CD_3)CO$, 300K, 500 MHz).

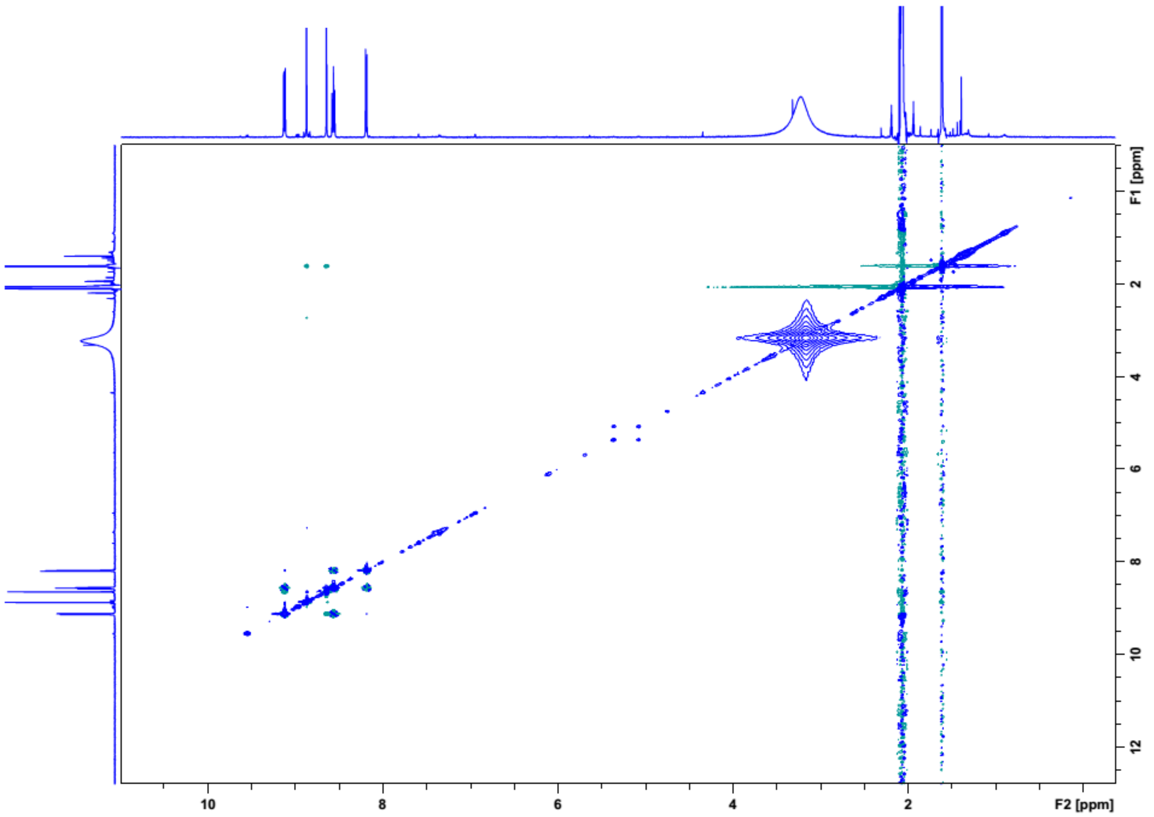

Figure S57. NOESY NMR spectrum of **6** ((CD$_3$)CO, 300K, 500 MHz).

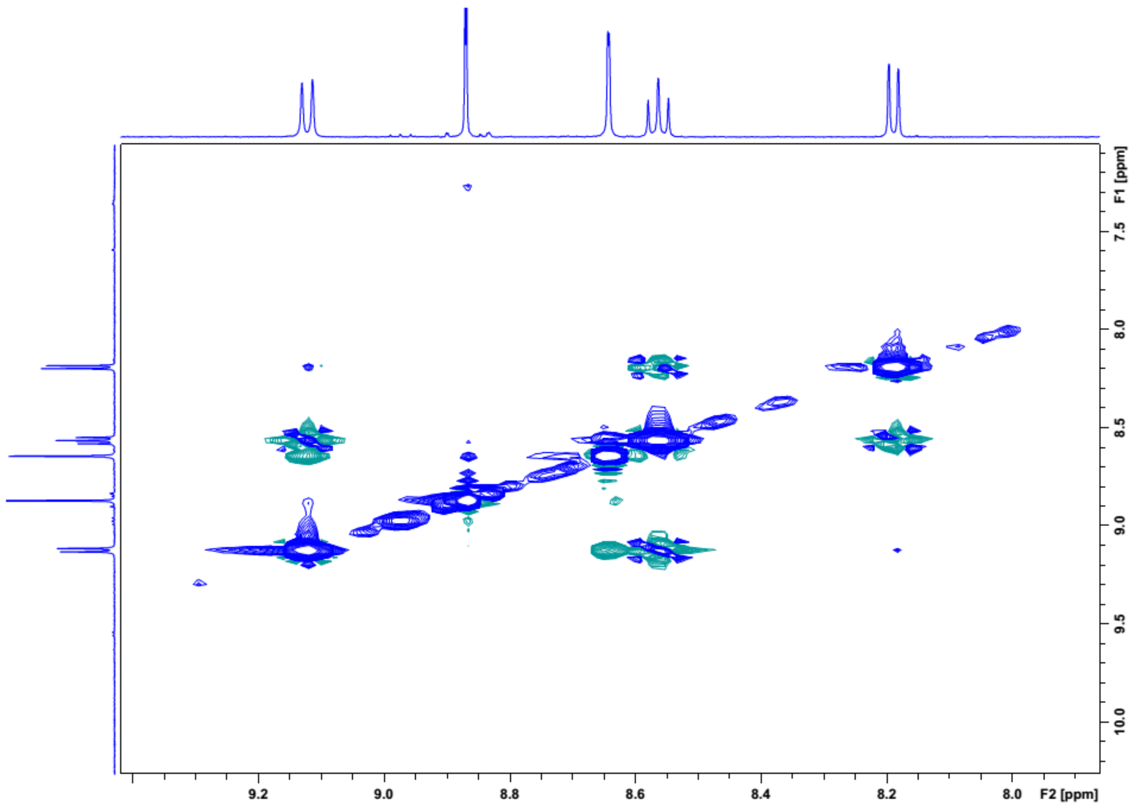

Figure S58. NOESY NMR spectrum (zoom, aromatic region) of **6** ((CD$_3$)CO, 300K, 500 MHz).

3.8 NMR spectra of 8

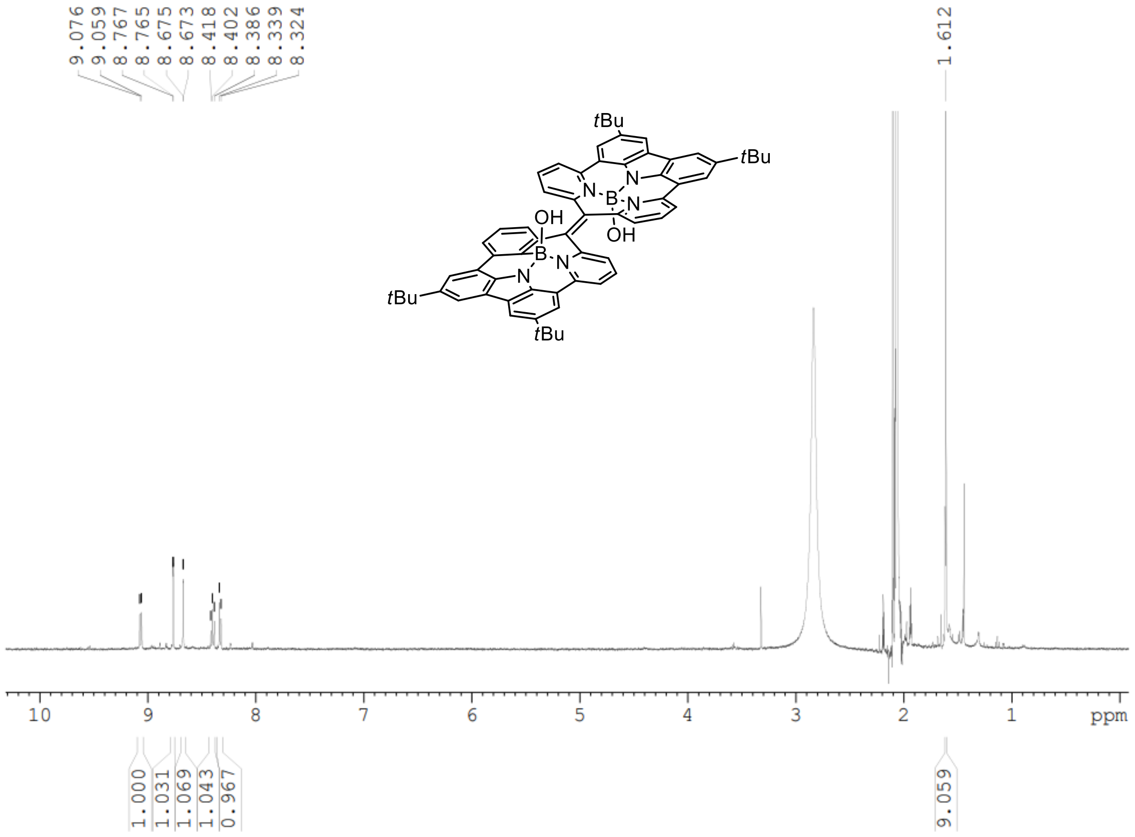

Figure S59. ^{1}H NMR spectrum of **8** ($(CD_3)CO$, 300K, 500 MHz).

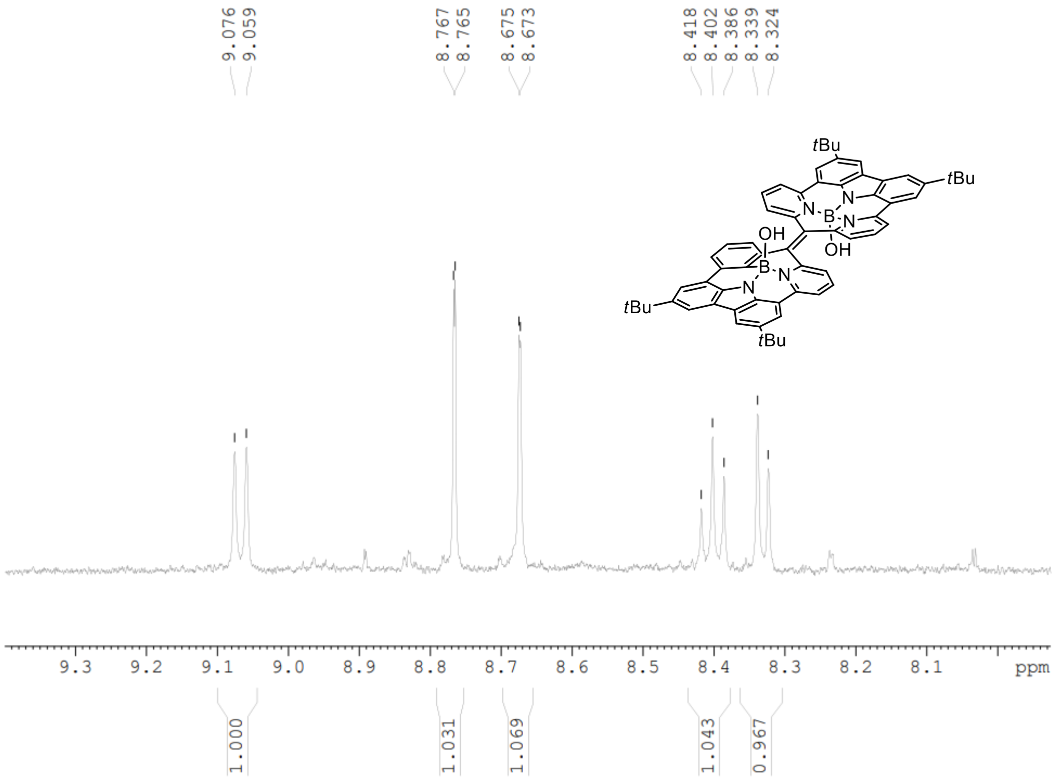

Figure S60. ^{1}H NMR spectrum (zoom, aromatic region) of **8** ($(CD_3)CO$, 300K, 500 MHz).

3.9 NMR spectra of incubation of 6 in MeOD

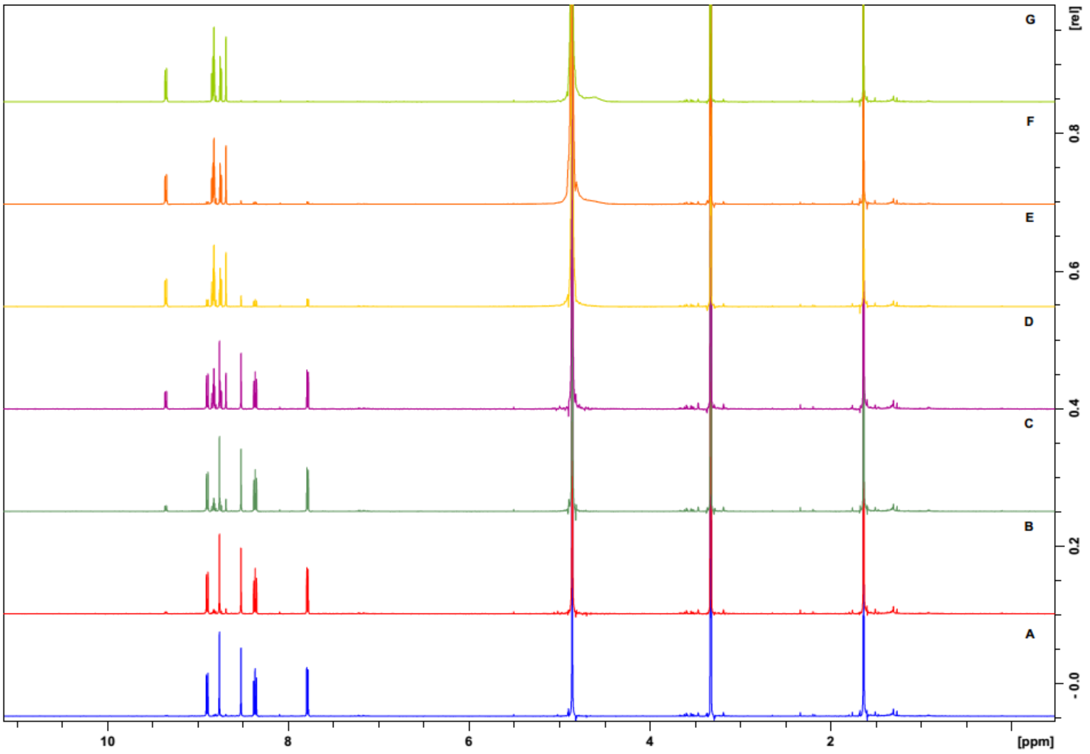

Figure S61. ^{1}H NMR spectra of **6** incubated in CD_3OD (A - start; B - 12 h; C - 24 h; D - 10 days; E - 19 days; F – 25 days; G – 36 days) (A - D – inert atmosphere, after 14 days the sample was exposed on air).

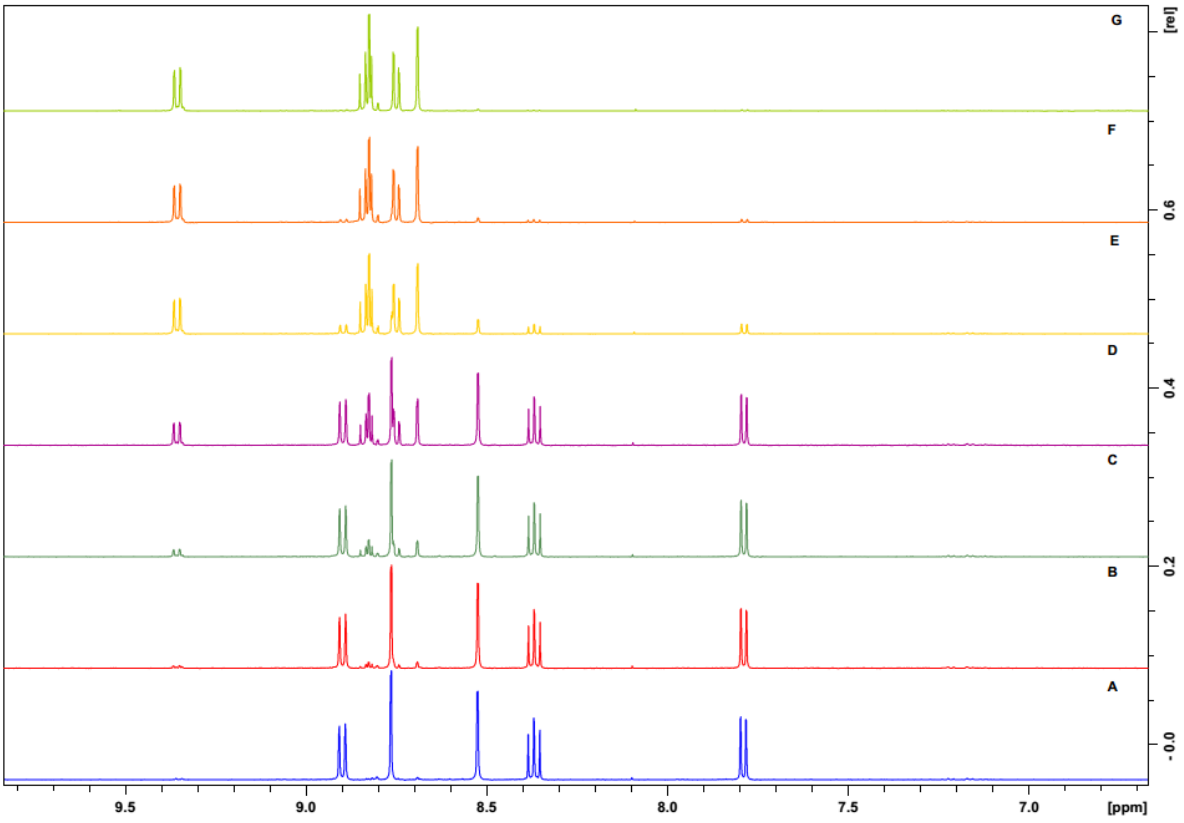

Figure S62. ^{1}H NMR spectra (zoom, aromatic region) of **6** incubated in CD_3OD (A - start; B - 12 h; C - 24 h; D - 10 days; E - 19 days; F – 25 days; G – 36 days) (A - D – inert atmosphere, after 14 days the sample was exposed on air).

4. LC-MS and MS spectra

4.1 ESI-MS spectrum of 2

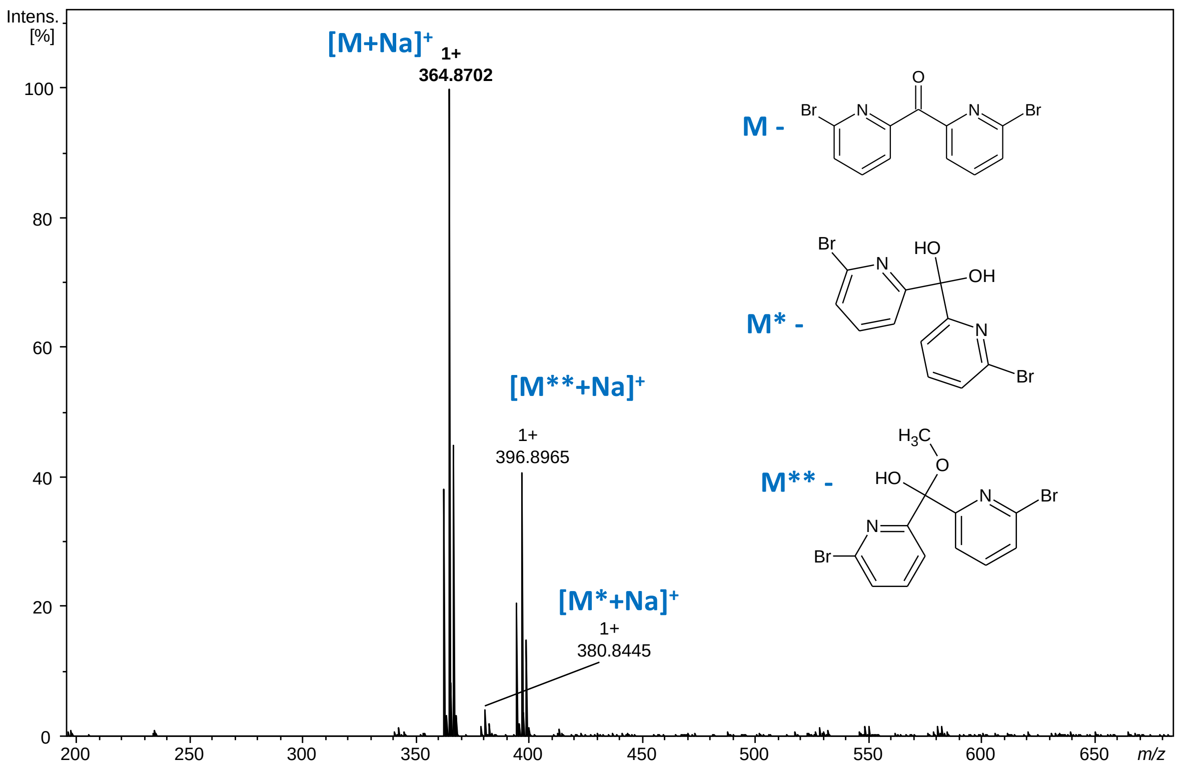

Figure S63. ESI-MS spectrum of **2** performed in 10 mM of NaCl in methanol (M*, M** - ions formed in the ion source).

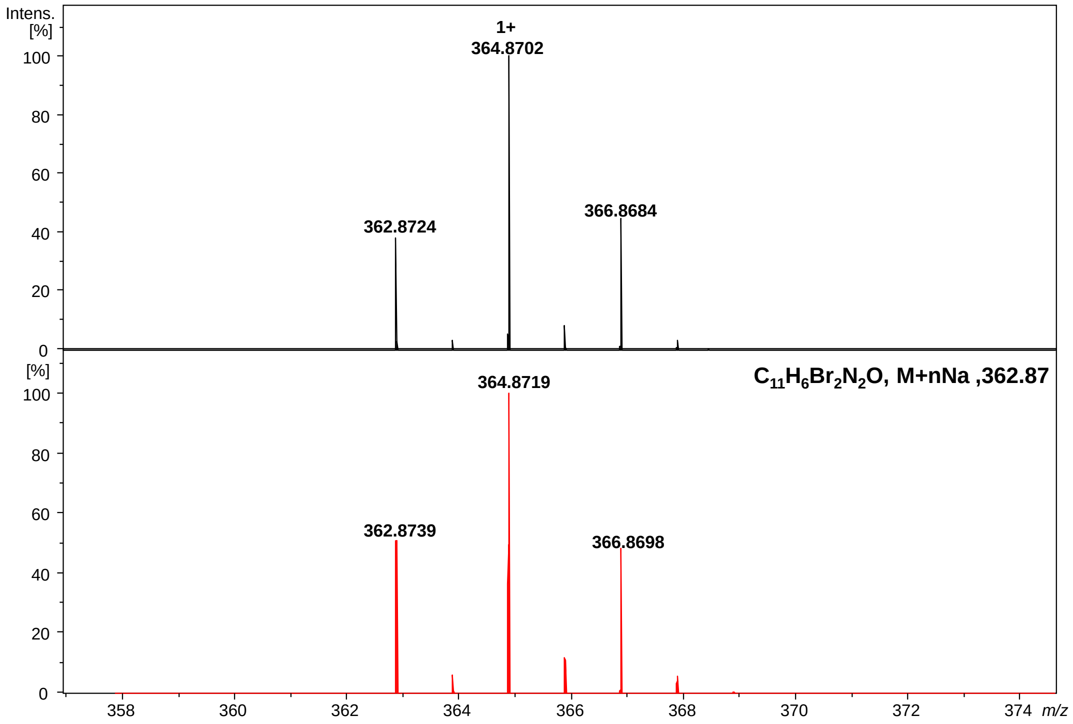

Figure S64. ESI-MS spectrum of **2** (zoom) with simulated spectrum (bottom panel - simulated isotopic patterns for molecular formula of investigated compounds).

4.2 ESI-MS spectrum of 3

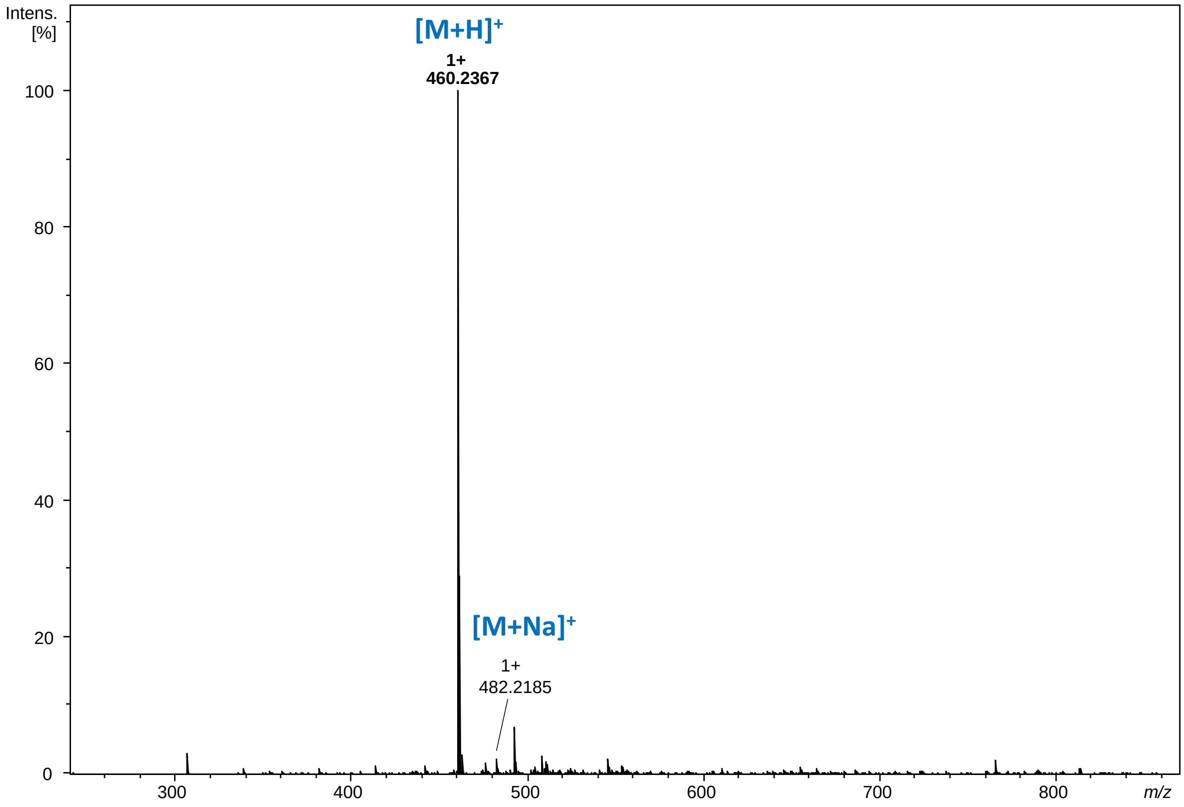

Figure S65. ESI-MS spectrum of **3**.

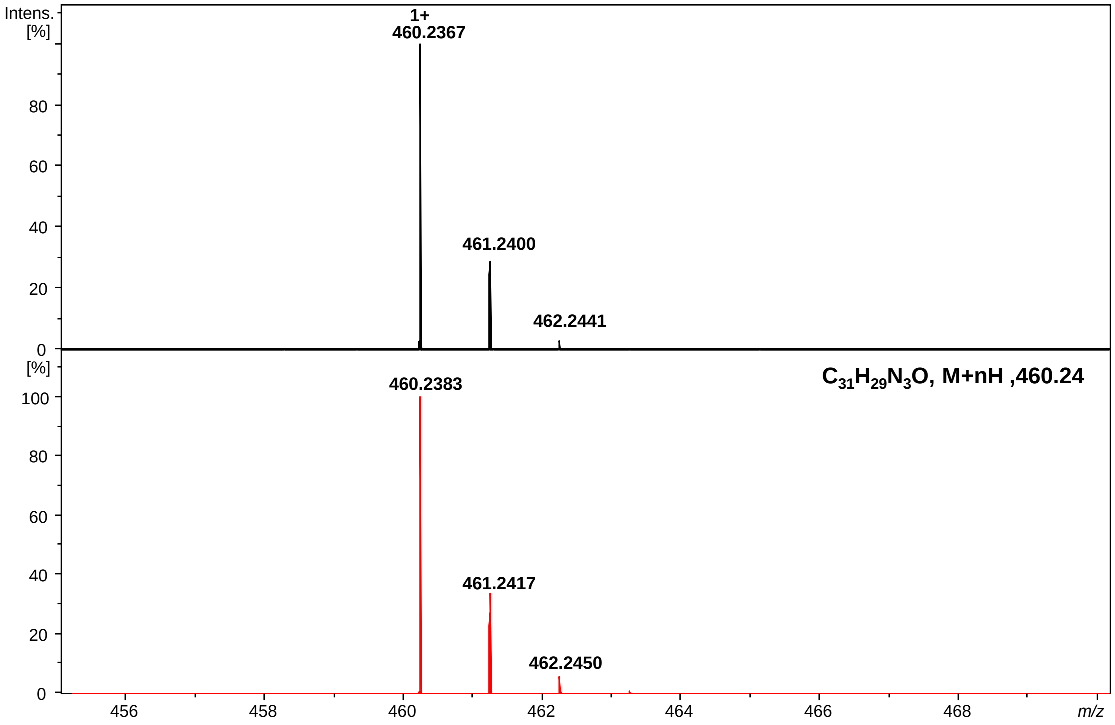

Figure S66. ESI-MS spectrum of **3** (zoom) with simulated spectrum (bottom panel - simulated isotopic patterns for molecular formula of investigated compounds).

4.3 ESI-MS spectrum of 4a

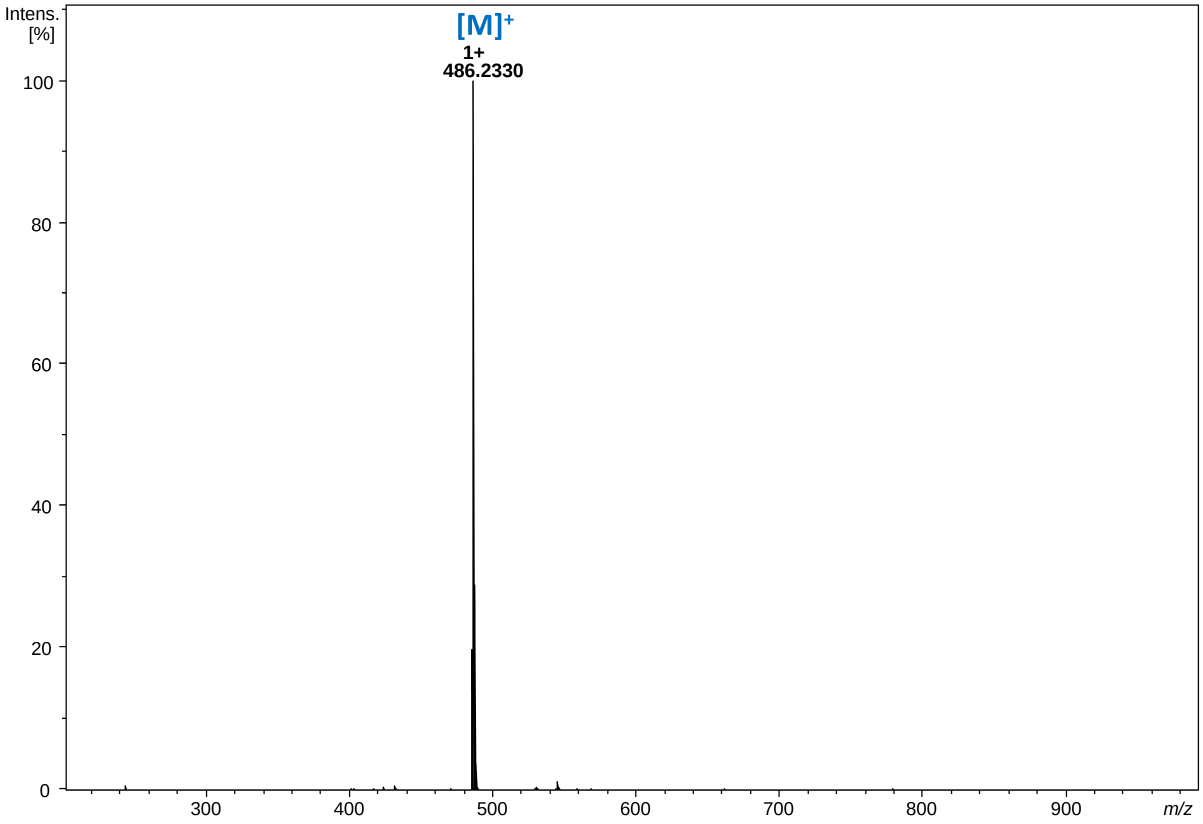

Figure S67. ESI-MS spectrum of **4a**.

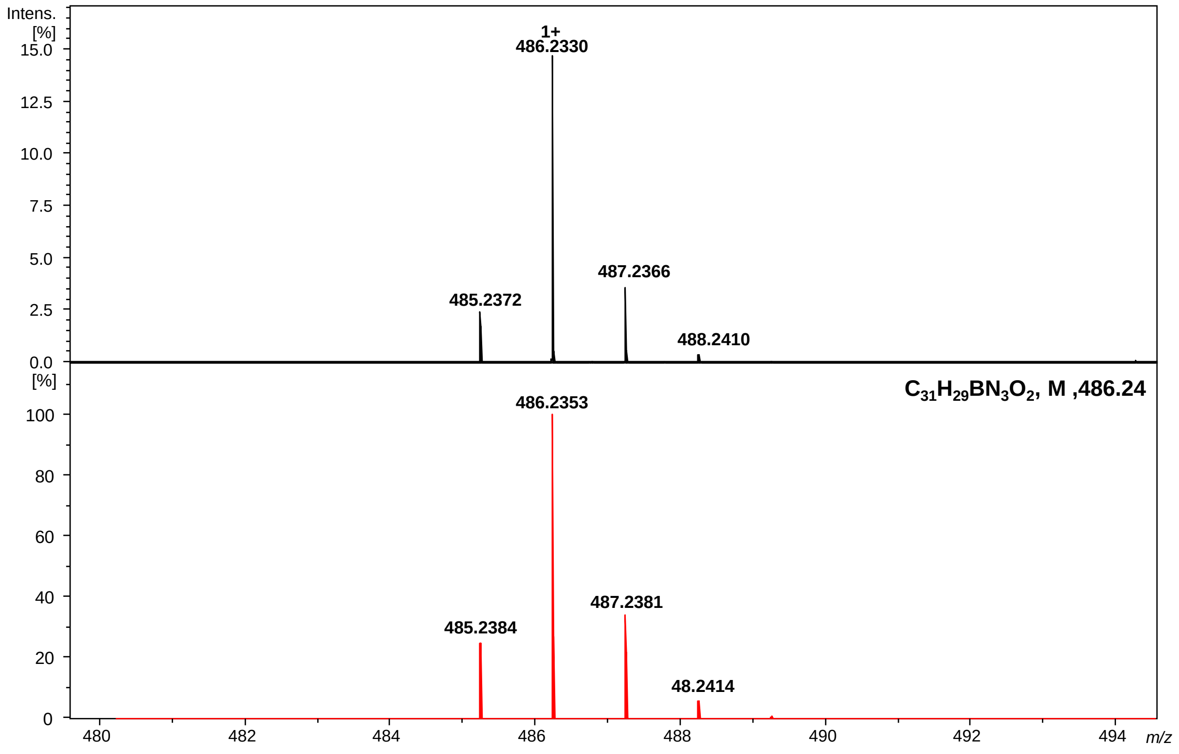

Figure S68. ESI-MS spectrum of **4a** (zoom) with simulated spectrum (bottom panel - simulated isotopic patterns for molecular formula of investigated compounds).

4.4 ESI-MS spectra of 4b

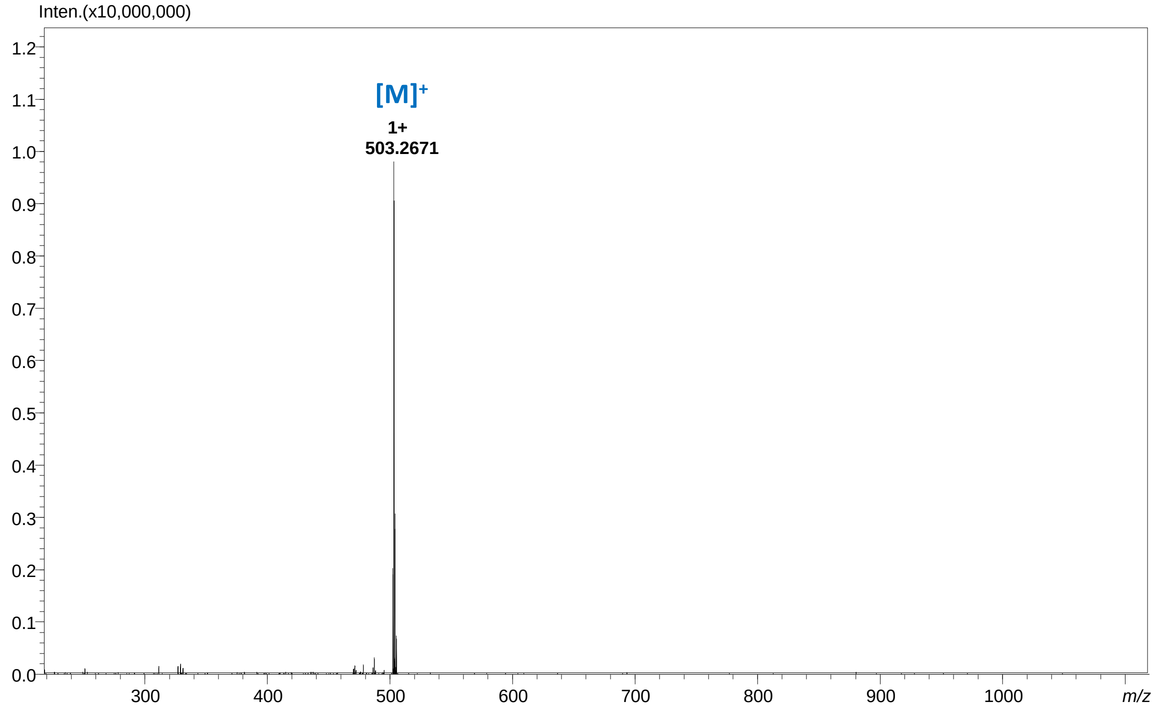

Figure S69. ESI-MS spectrum of **4b**.

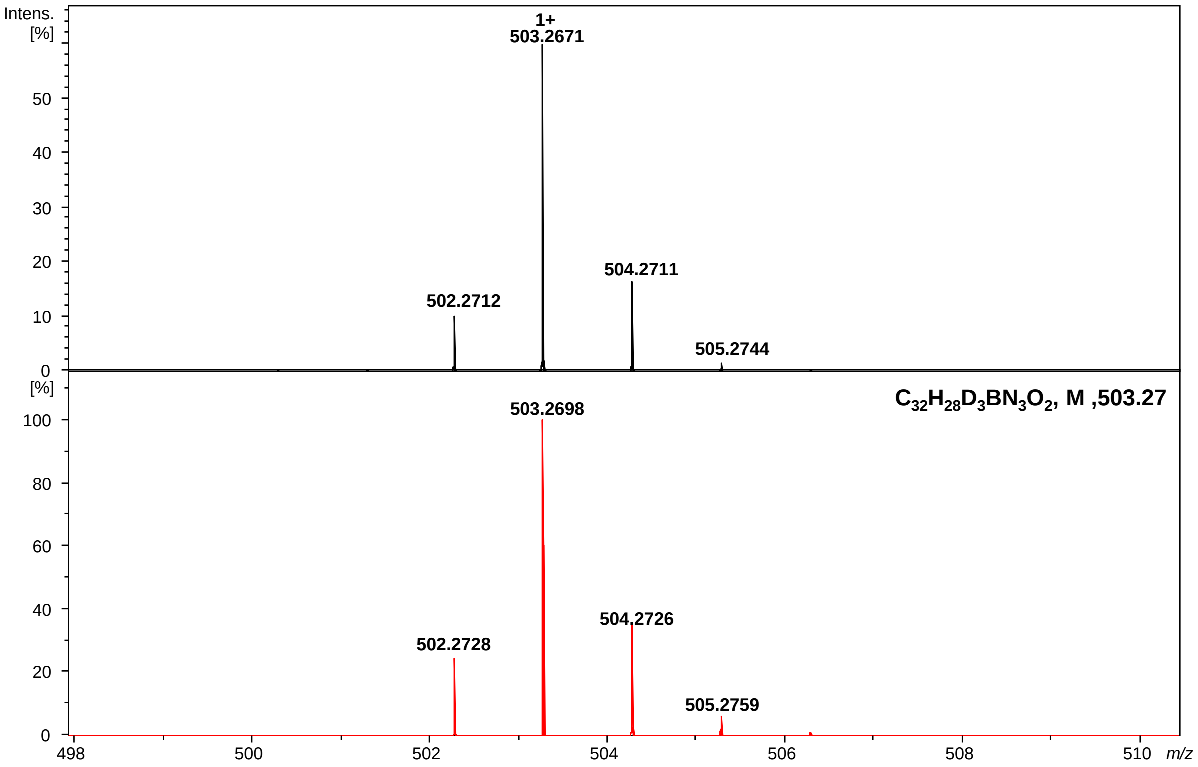

Figure S70. ESI-MS spectrum of **4b** (zoom) with simulated spectrum (bottom panel - simulated isotopic patterns for molecular formula of investigated compounds).

4.5 ESI-MS spectra of 4c

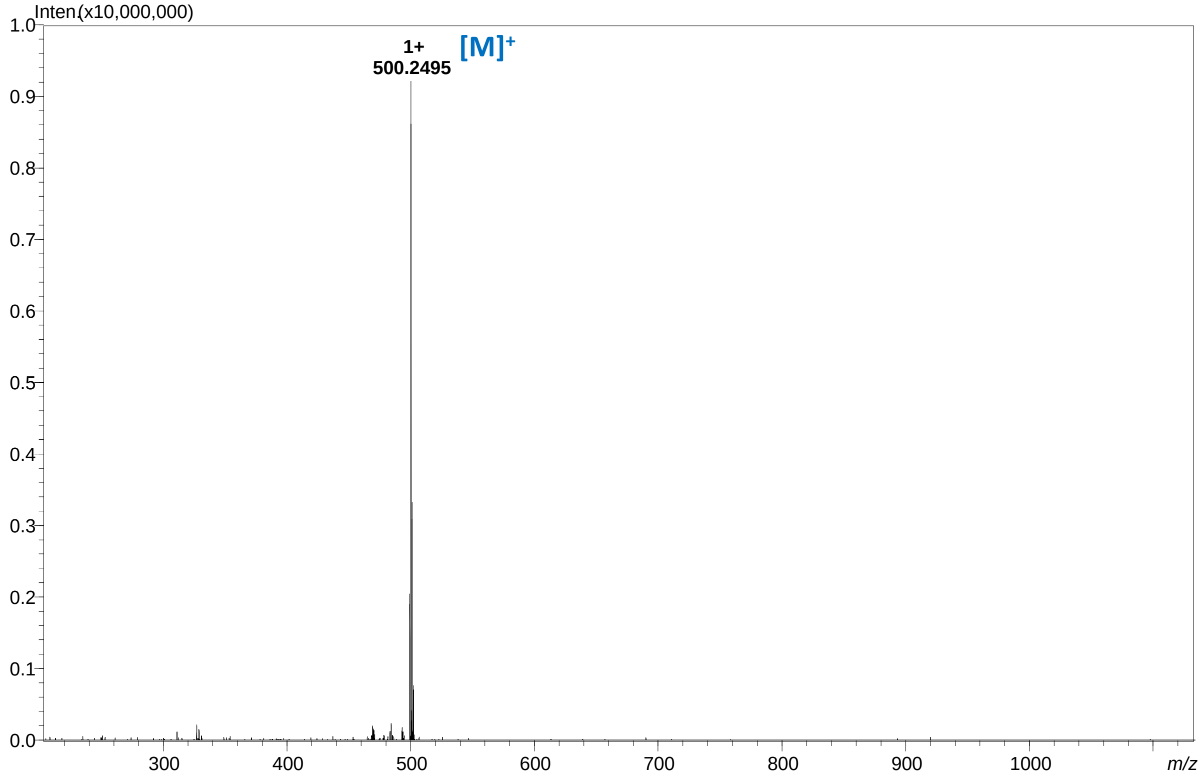

Figure S71. ESI-MS spectrum of **4c**

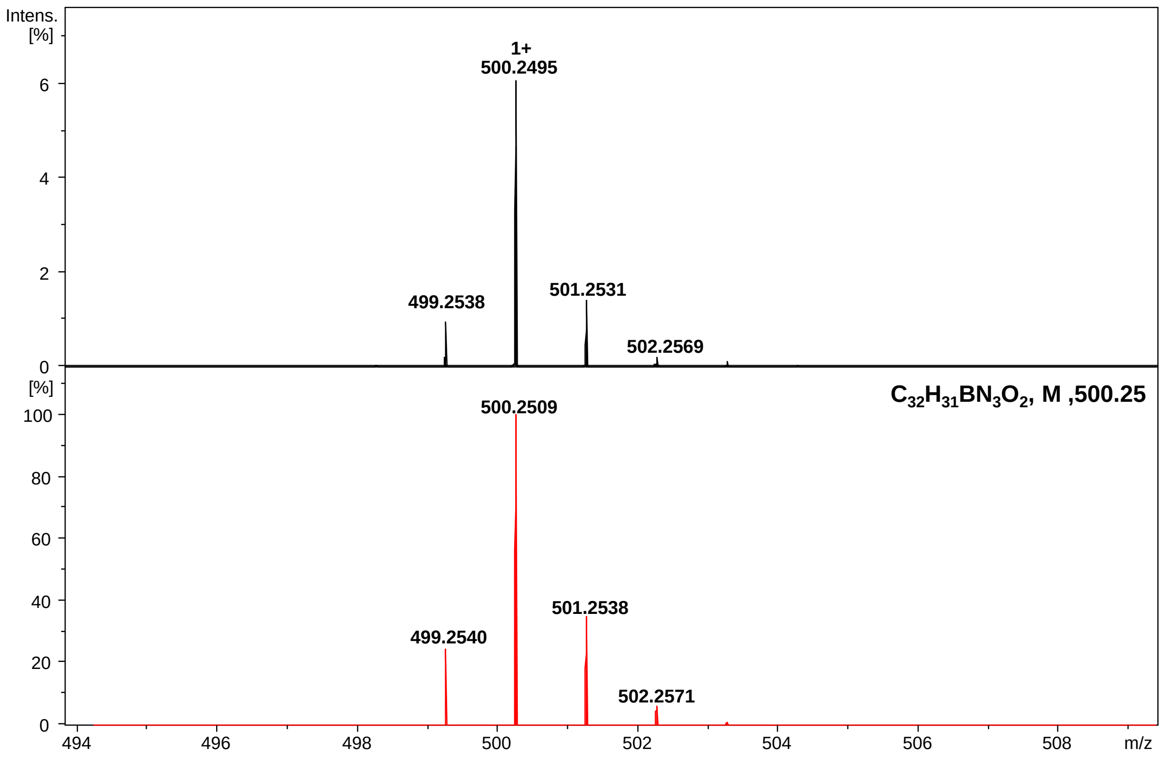

Figure S72. ESI-MS spectrum of **4c** (zoom) with simulated spectrum (bottom panel - simulated isotopic patterns for molecular formula of investigated compounds).

4.6 ESI-MS spectrum of 4-Ac

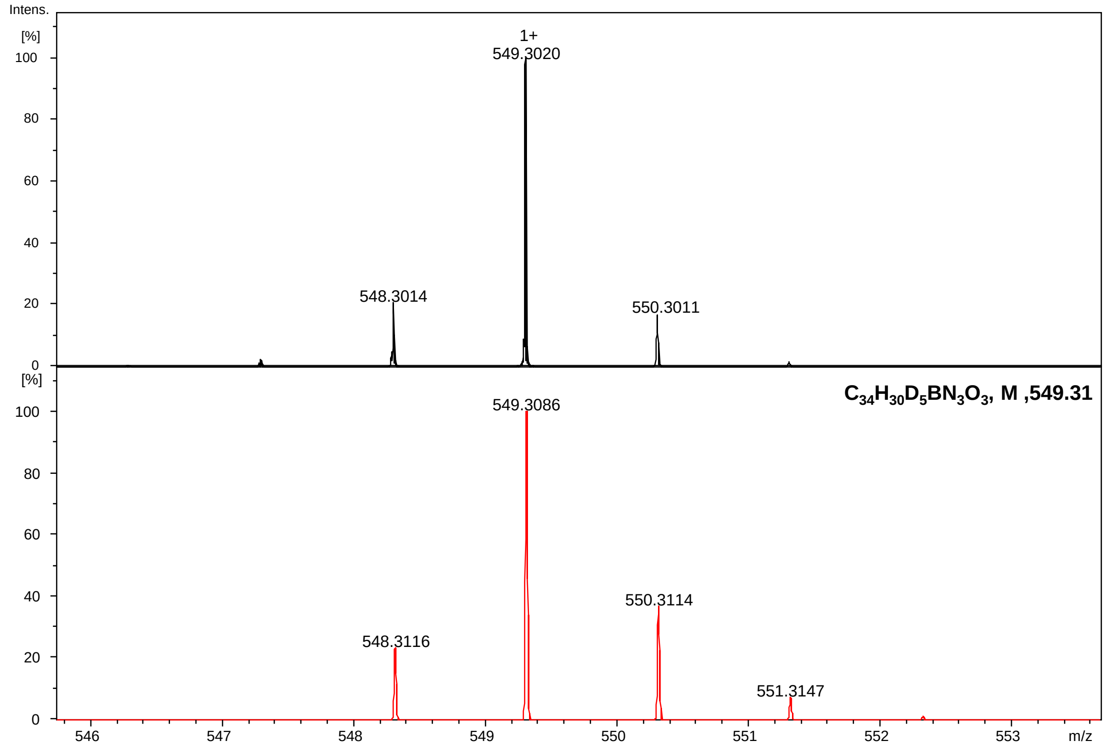

Figure S73. ESI-MS spectrum of **4-Ac** (zoom) with simulated spectrum (bottom panel - simulated isotopic patterns for molecular formula of investigated compounds).

4.7 ESI-MS spectrum of 5

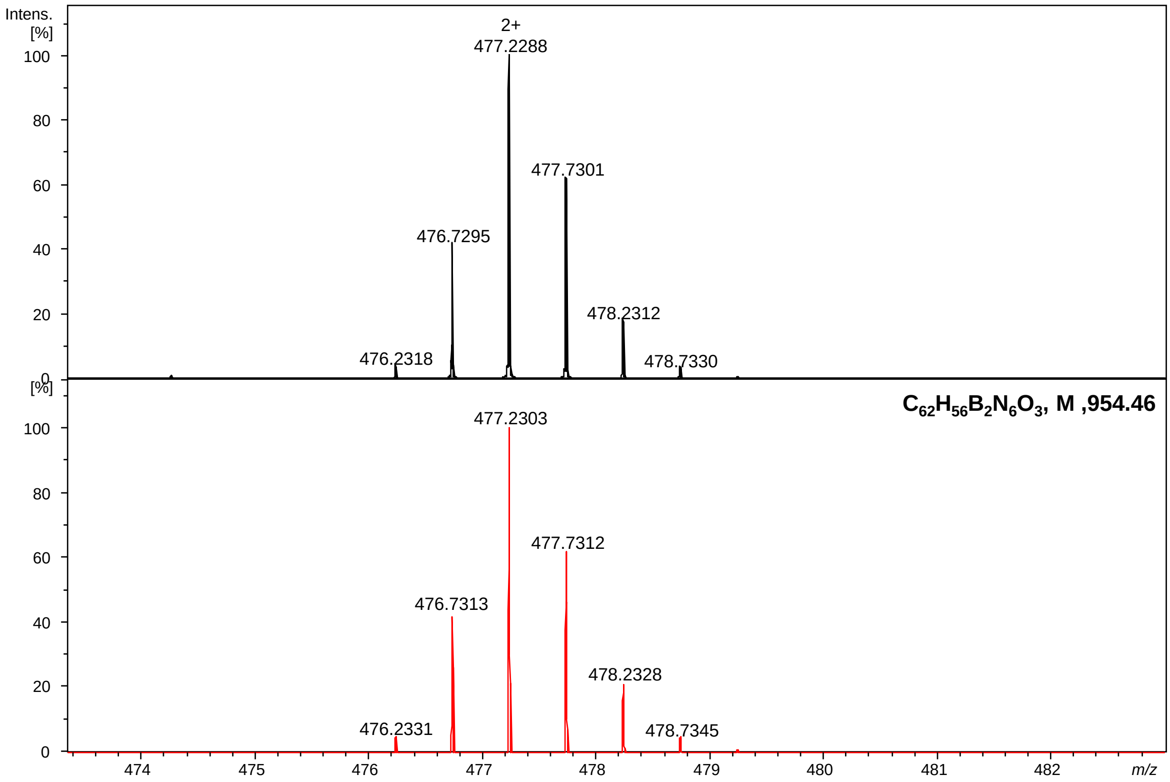

Figure S74. ESI-MS spectrum of **5** (zoom) with simulated spectrum (bottom panel - simulated isotopic patterns for molecular formula of investigated compounds).

4.8 ESI-MS spectrum of 5-Ac

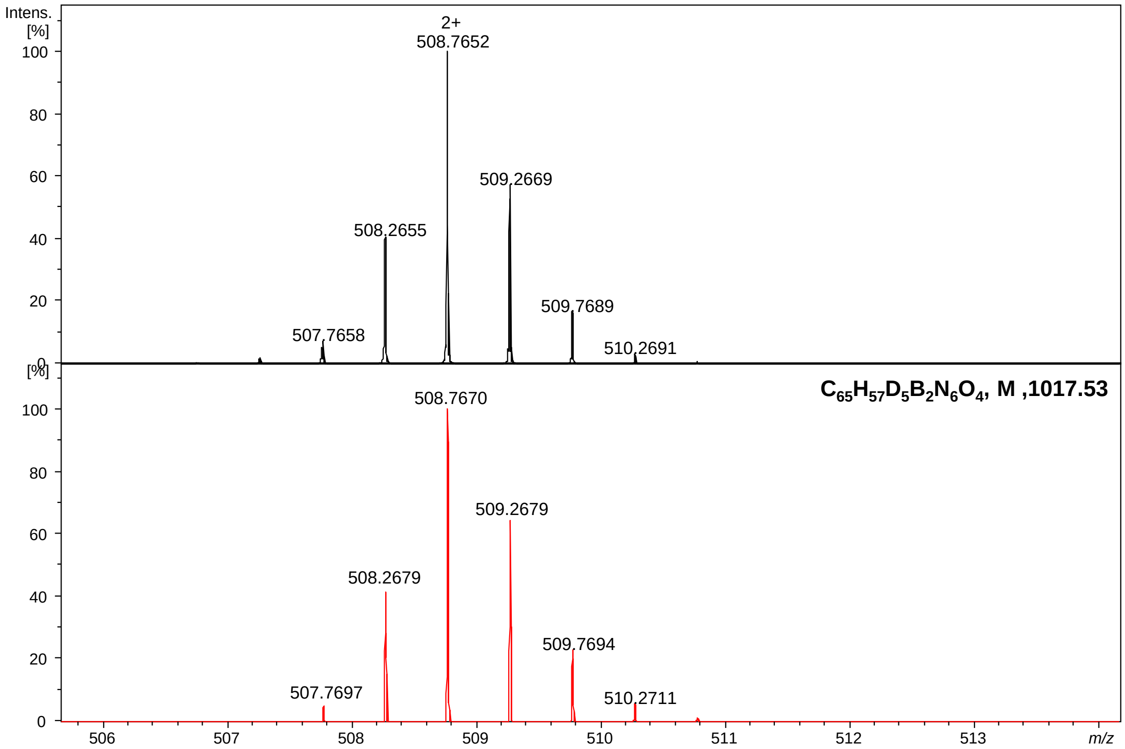

Figure S75. ESI-MS spectrum of **5-Ac** (zoom) with simulated spectrum (bottom panel - simulated isotopic patterns for molecular formula of investigated compounds).

4.9 ESI-MS spectrum of 6

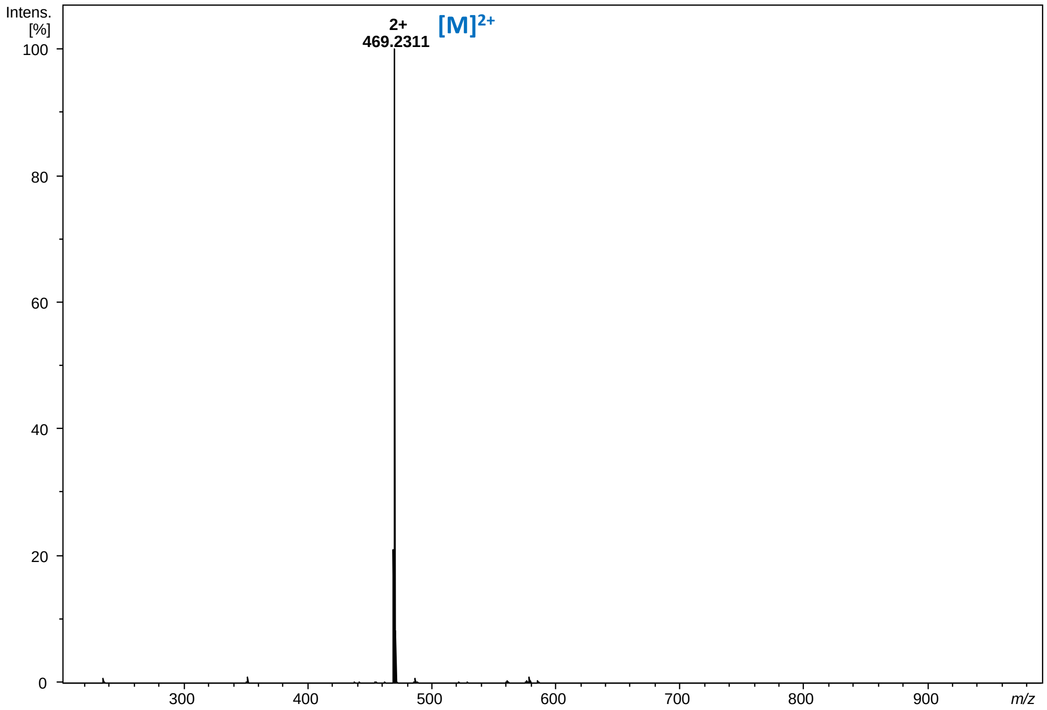

Figure S76. ESI-MS spectrum of **6**.

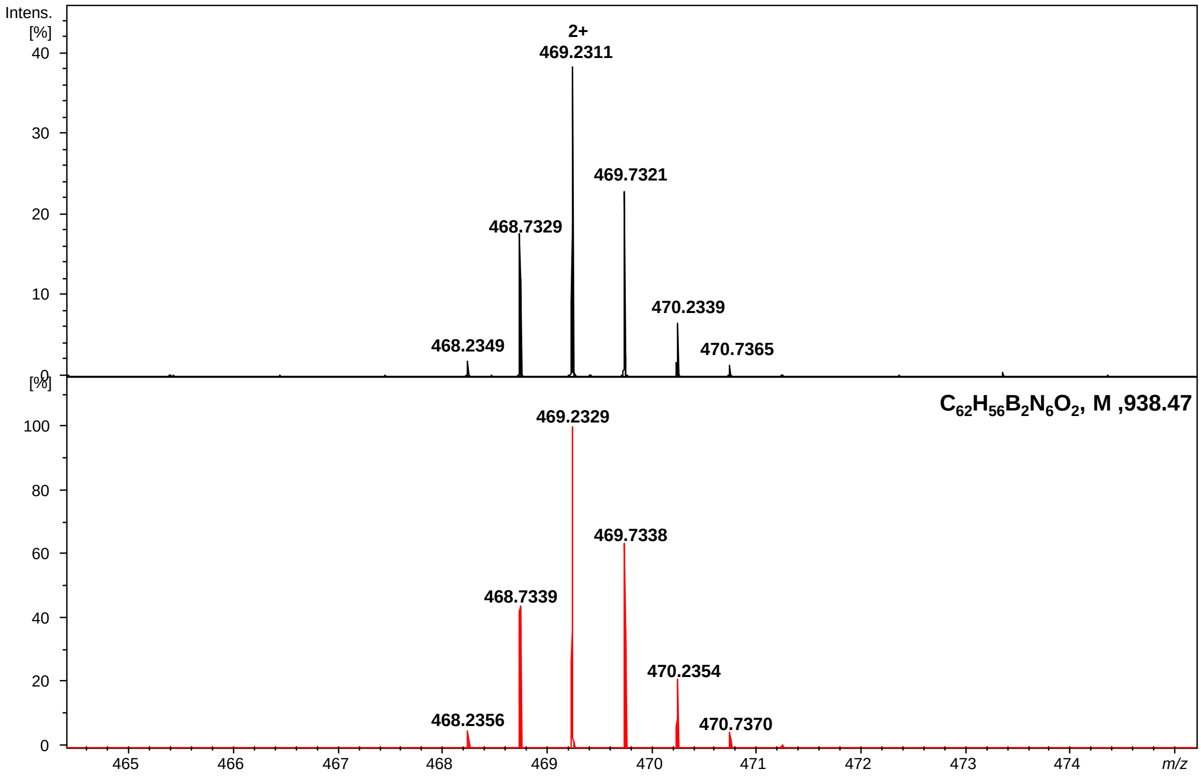

Figure S77 ESI-MS spectrum of **6** (zoom) with simulated spectrum (bottom panel - simulated isotopic patterns for molecular formula of investigated compounds).

4.10 ESI-MS spectrum of 8

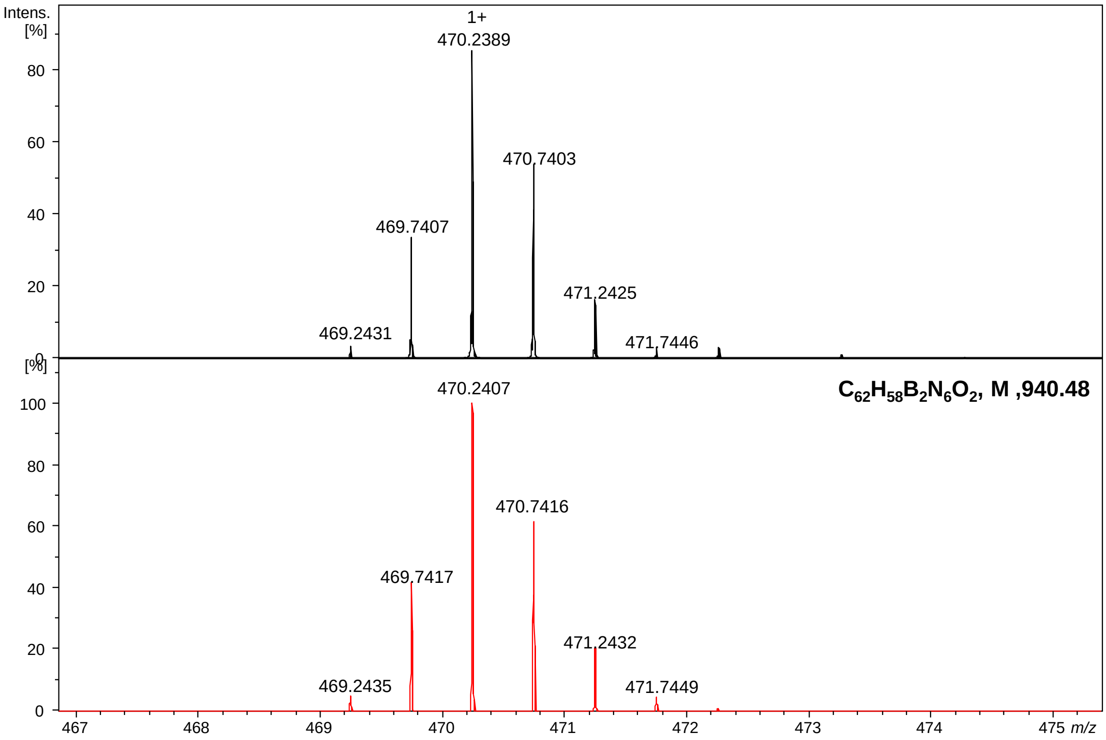

Figure S78 ESI-MS spectrum of **8** (zoom) with simulated spectrum (bottom panel - simulated isotopic patterns for molecular formula of investigated compounds).

4.11 LC-MS of crude mixture (Procedure 1)

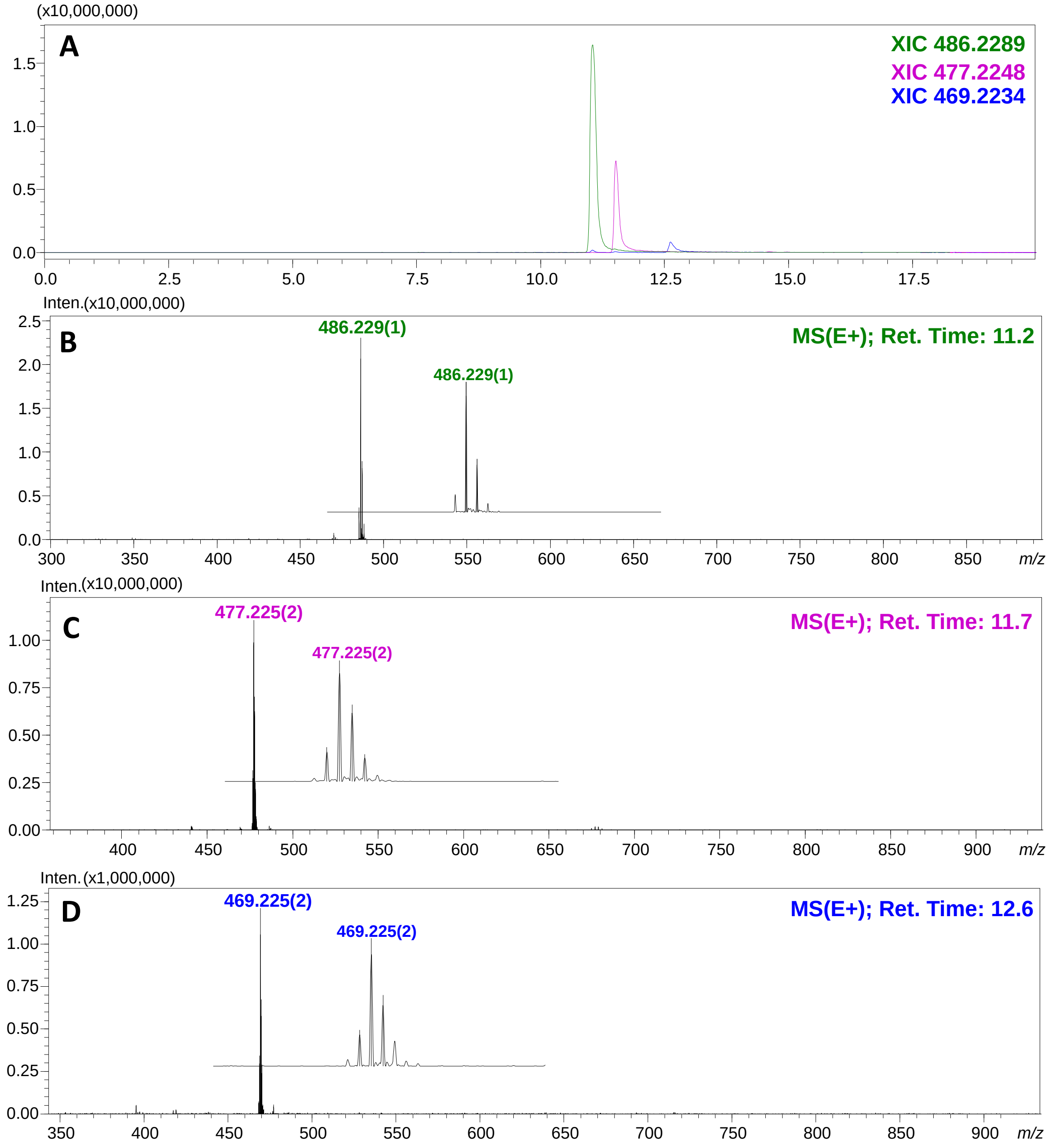

Figure S79 LC-MS analysis of crude mixture (MS value *m/z* = 469.223; *m/z*=477.225; *m/z*=486.229) (A); ESI-MS spectrum (B-D).

4.12 LC-MS of crude mixture (Procedure 2)

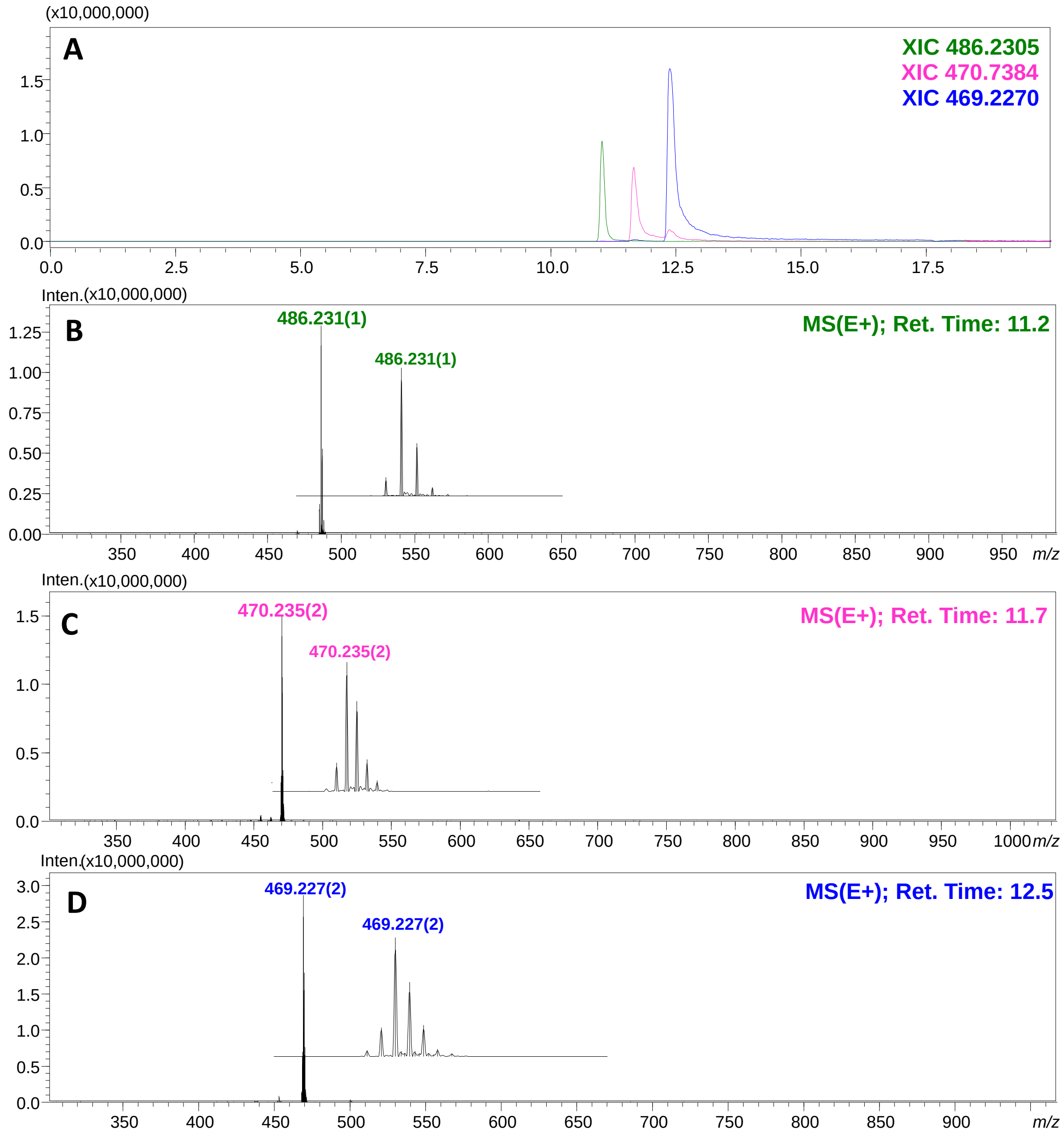

Figure S80 LC-MS analysis of crude mixture (MS value *m/z*=469.227; *m/z*=486.230; *m/z*=470.235) (A); ESI-MS spectrum (B-D).

4.13 LC-MS of 4a

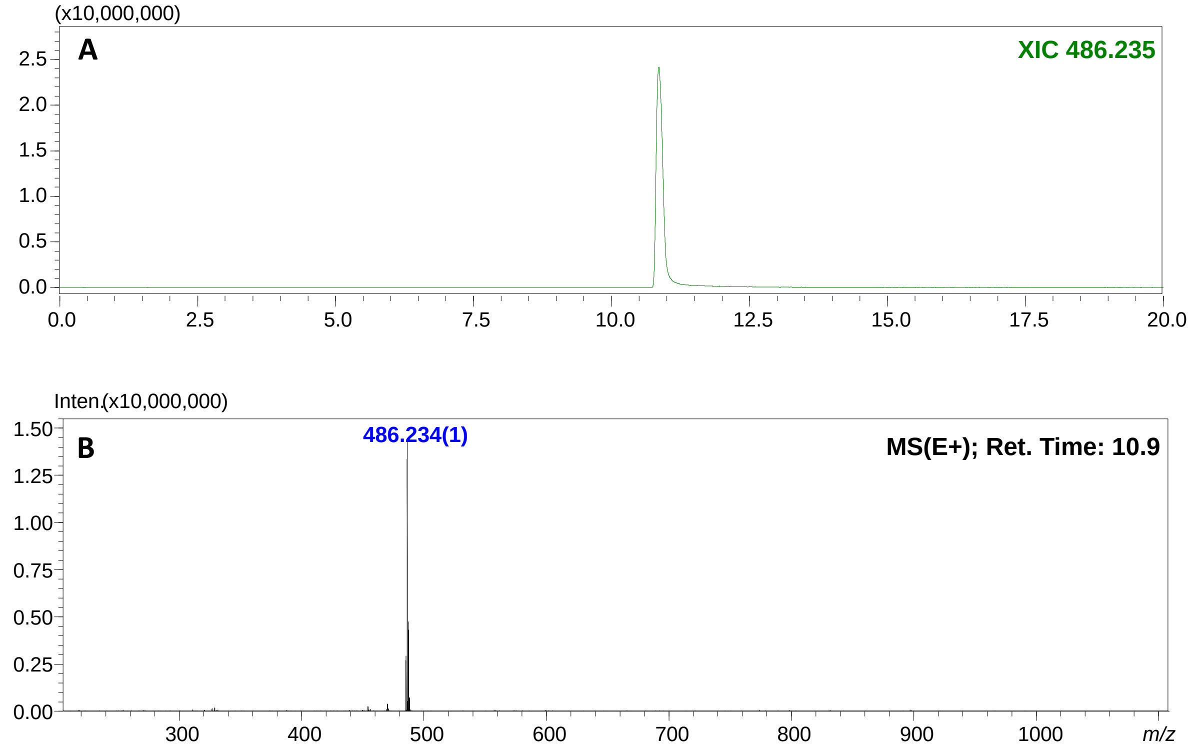

Figure S81 LC-MS analysis of **4a** (MS value m/z = 486.234) (A); ESI-MS spectrum (B).

4.14 LC-MS of 4b

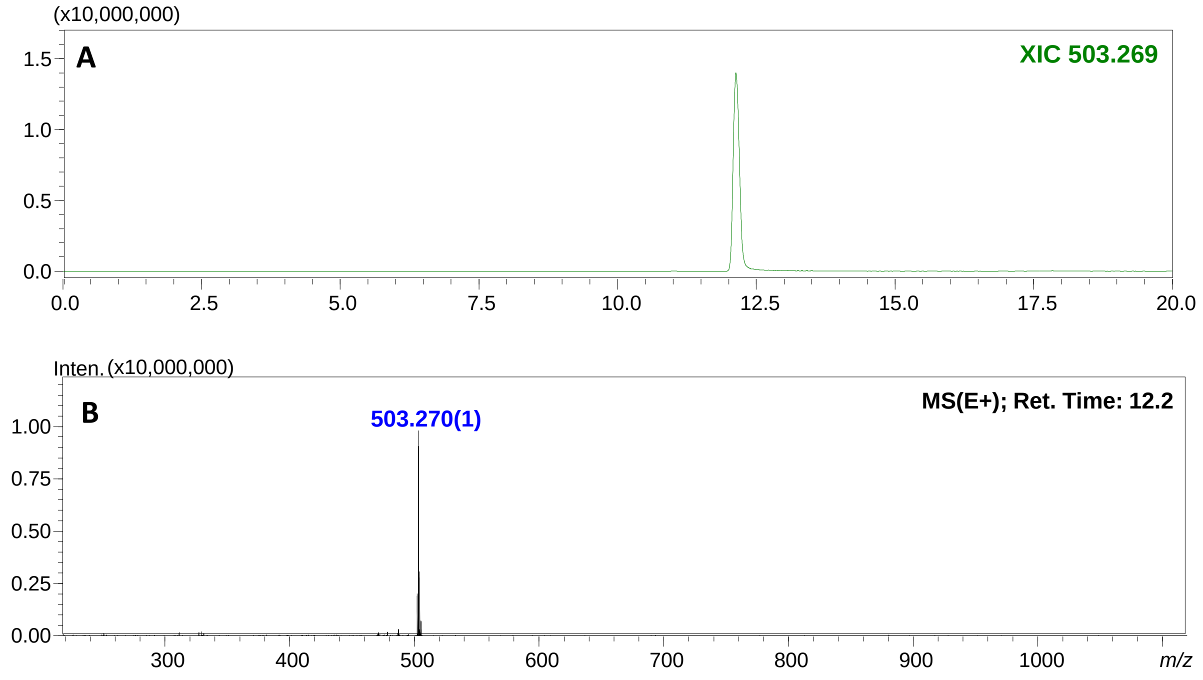

Figure S82 LC-MS analysis of **4b** (MS value m/z = 503.270) (A); ESI-MS spectrum (B).

4.15 LC-MS of 4c

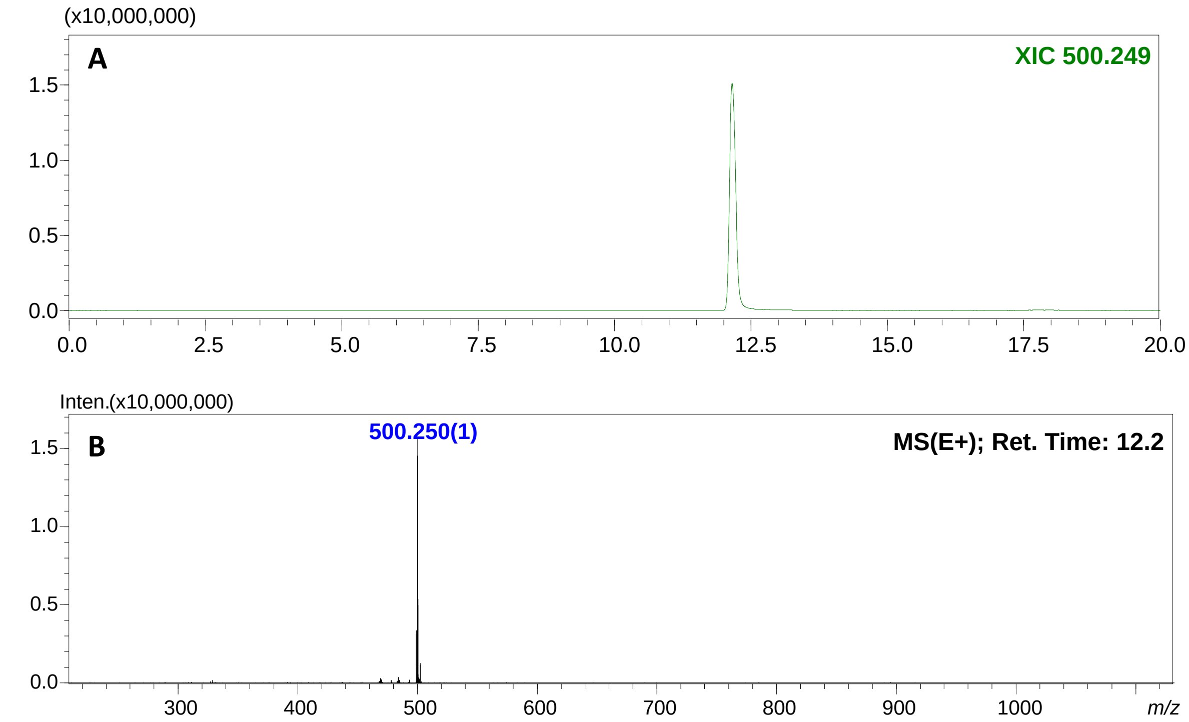

Figure S83 LC-MS analysis of **4c** (MS value m/z = 500.250) (A); ESI-MS spectrum (B).

4.16 LC-MS of 5

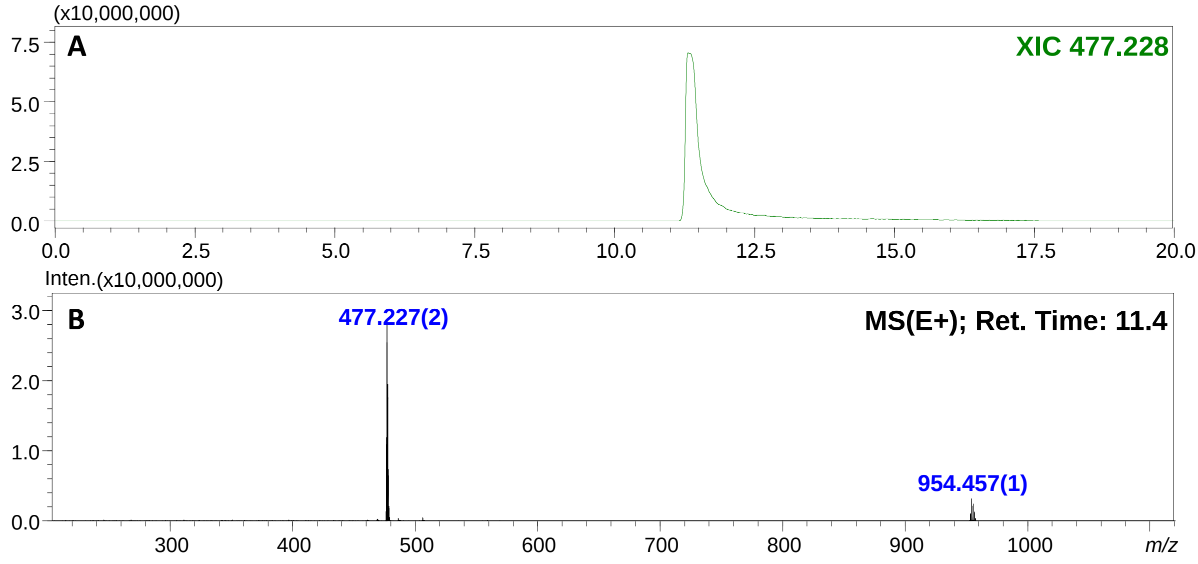

Figure S84 LC-MS analysis of **5** (MS value m/z = 477.227) (A); ESI-MS spectrum (B).

4.17 LC-MS of 6

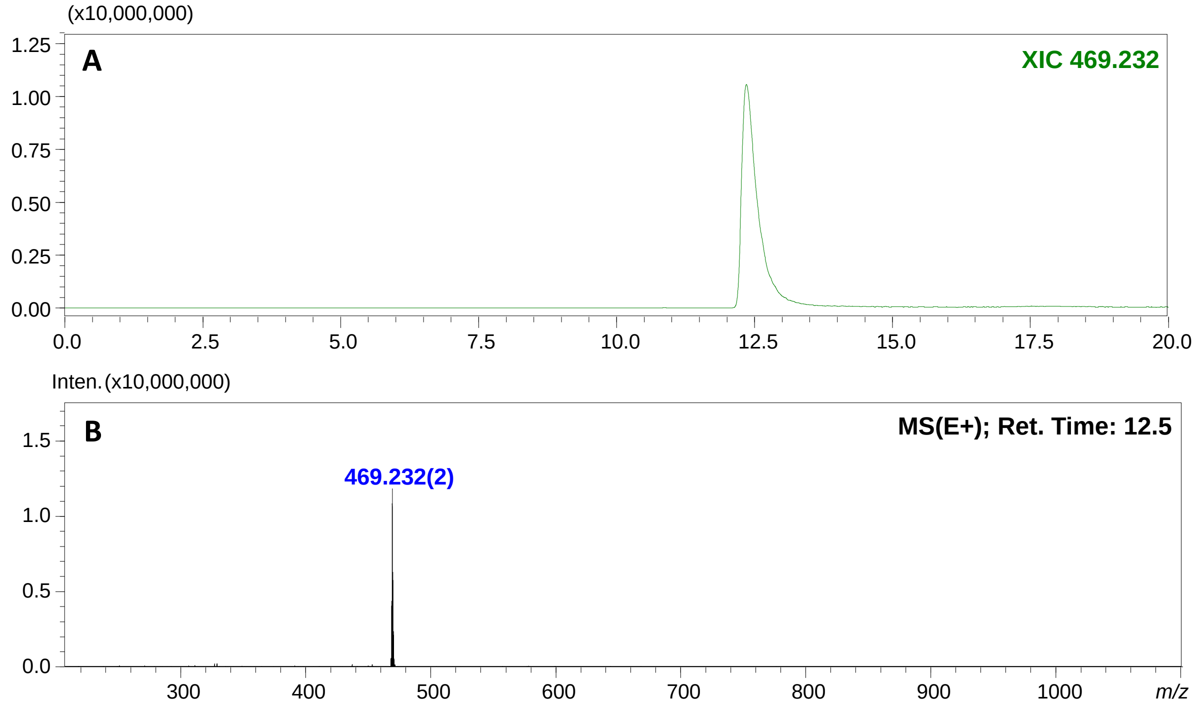

Figure S85 LC-MS analysis of **6** (MS value m/z = 469.232) (A); ESI-MS spectrum (B).

4.18 LC-MS of 8

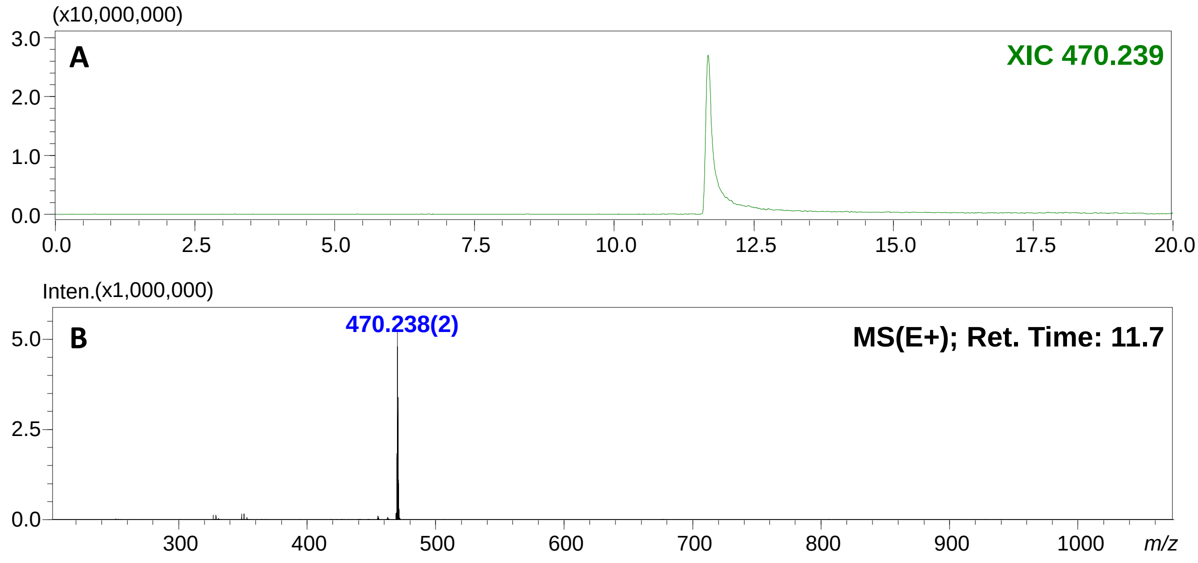

Figure S86 LC-MS analysis of **8** (MS value m/z = 477.238) (A); ESI-MS spectrum (B).

4.19 LC-MS of incubation of 6 in water-acetonitrile (v:v, 1:1) in 60°C for 12 h

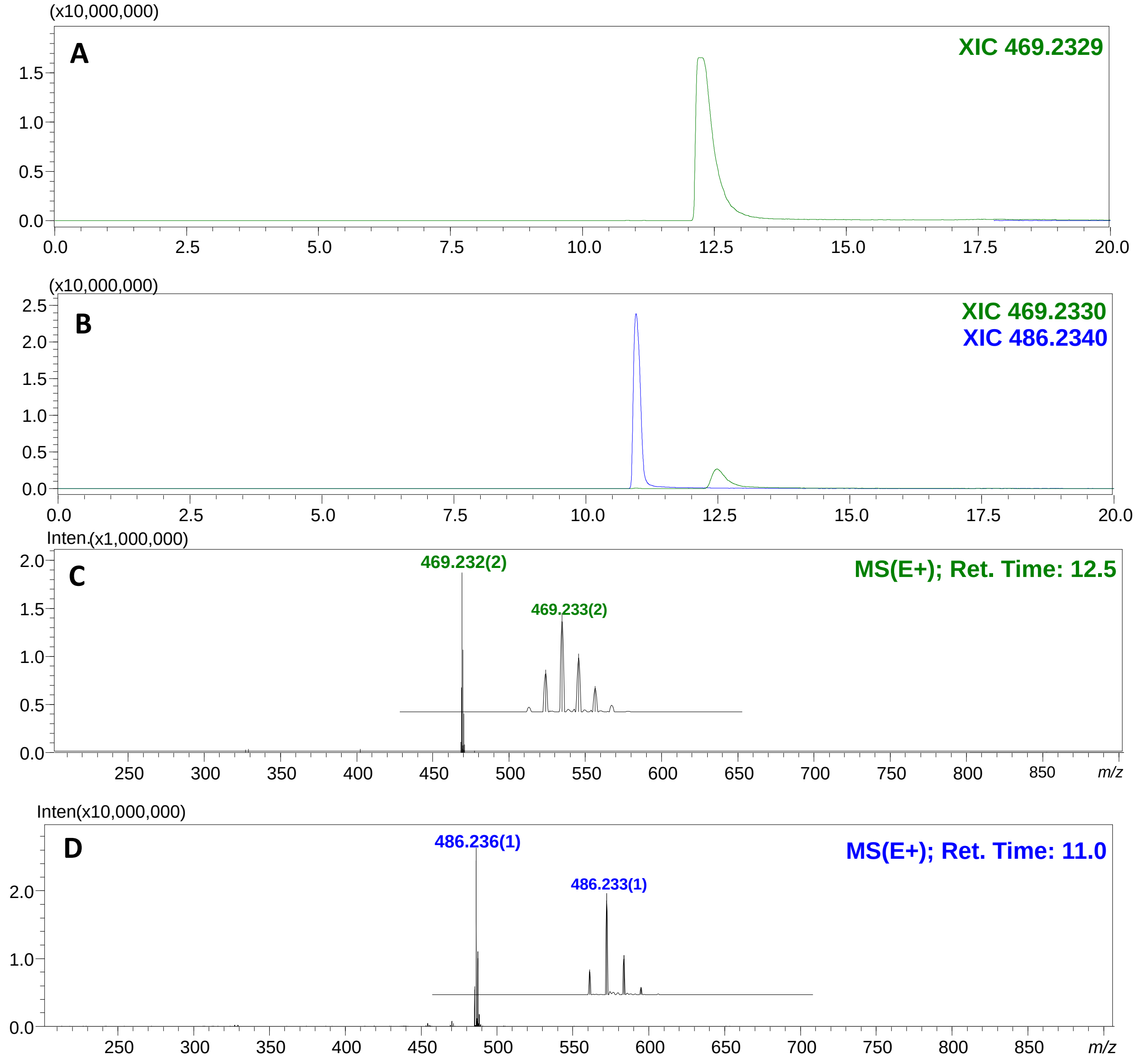

Figure S87 LC-MS analysis before (A) and after (B) incubation of **6** in water-acetonitrile (1:1, v:v) in 60°C for 12 h (MS value *m/z* = 469.233; *m/z* = 486.234); ESI-MS spectra (C,D).

4.20 LC-MS of incubation of 4a in water-acetonitrile (v:v, 1:1) in 60°C for 12h

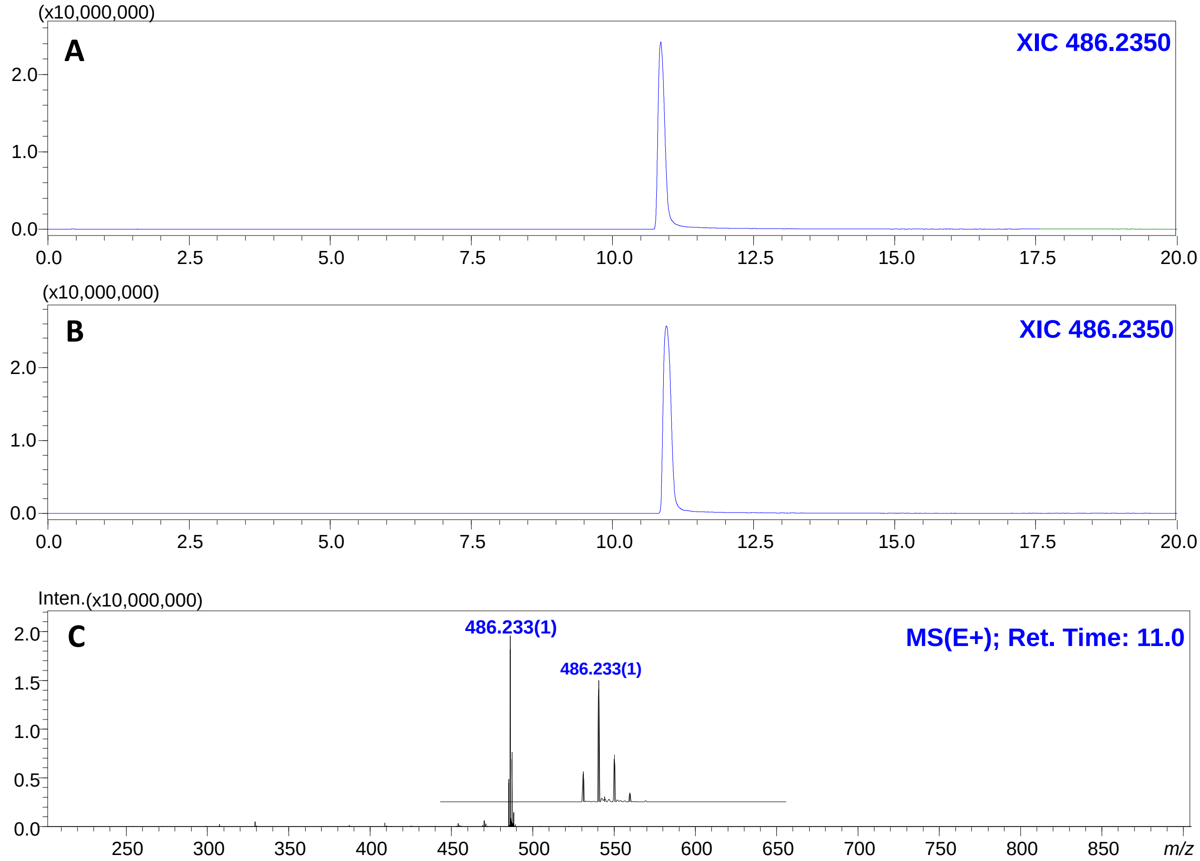

Figure S88 LC-MS analysis before (A) and after (B) incubation of **4a** in water-acetonitrile (1:1, v:v) in 60°C for 12 h (MS value *m/z* = 486.234); ESI-MS spectrum (C).

4.21 LC-MS of incubation of 6 in CD_3OD in 60°C for 12 h

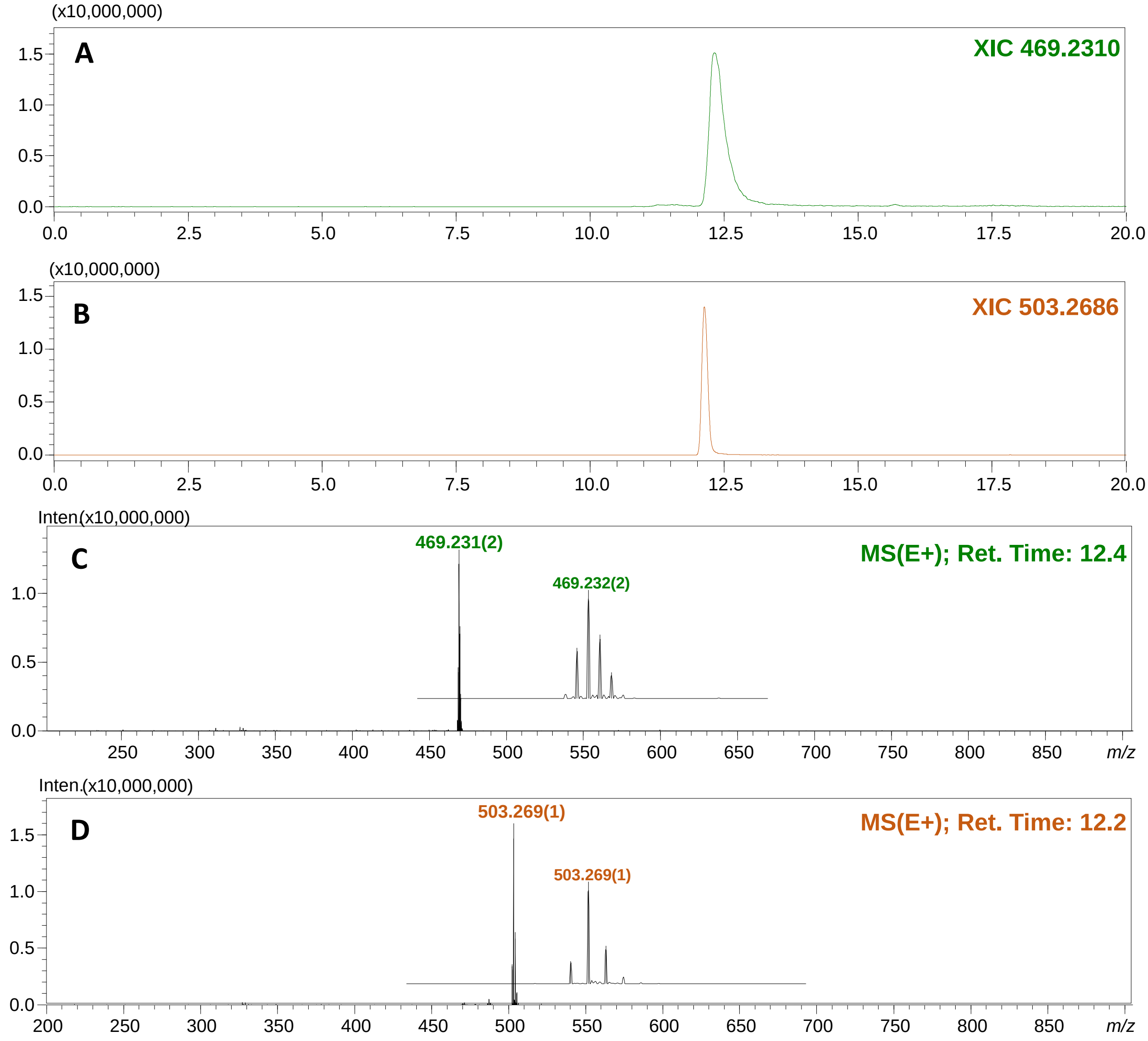

Figure S89 LC-MS analysis before (A) and after (B) incubation of **6** in CD_3OD in 60°C for 12 h (MS value *m/z* = 469.231; *m/z* = 503.269) ; ESI-MS spectra (C,D).

4.22 LC-MS of incubation of 6 in MeOH in 60°C for 2 h

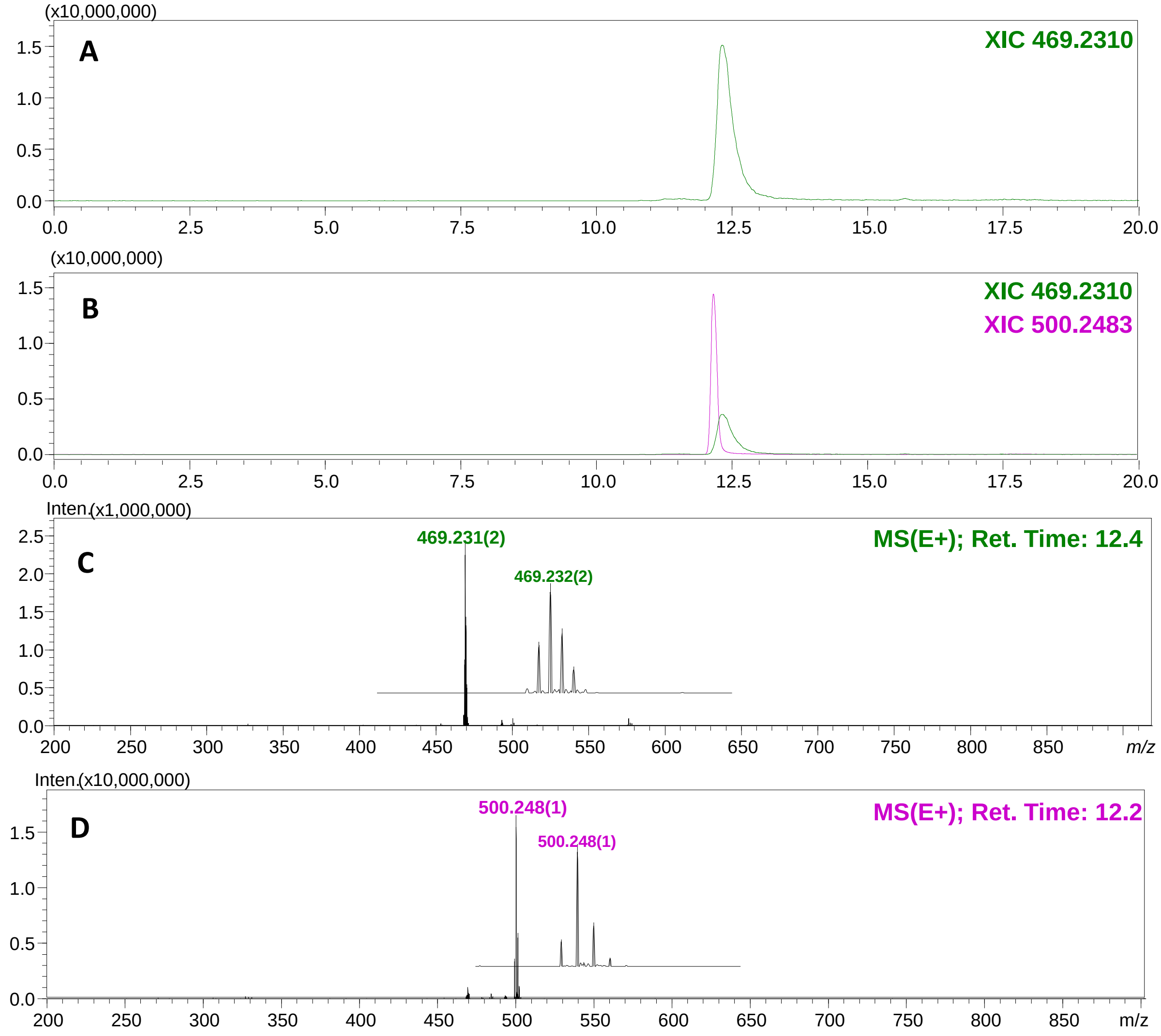

Figure S90 LC-MS analysis before (A) and after (B) incubation of **6** in MeOH in 60°C for 2 h (MS value *m/z* = 469.231; *m/z* = 500.248) ; ESI-MS spectra (C,D).

4.23 LC-MS of incubation of mixture of 4a and 6 in CD_3OD in 60°C for 2 h

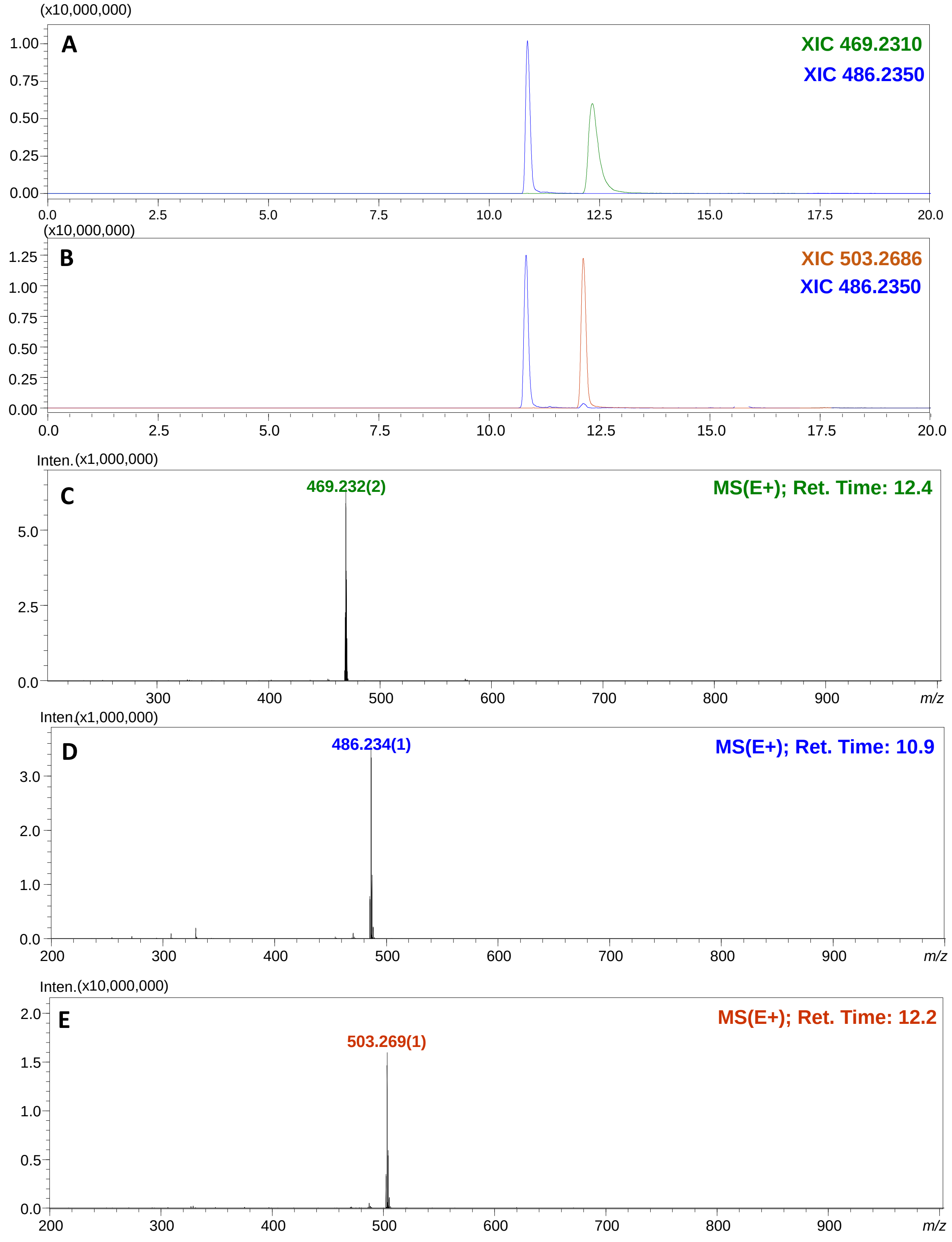

Figure S91 LC-MS analysis before (A) and after (B) incubation of mixture of **6** and **4a** in CD_3OD in 60°C for 2 h (MS value *m/z* = 486.234; *m/z* = 469.231; *m/z* = 503.269) ; ESI-MS spectra (C-E).

4.24 LC-MS of incubation of pure 5 in acetone-d6

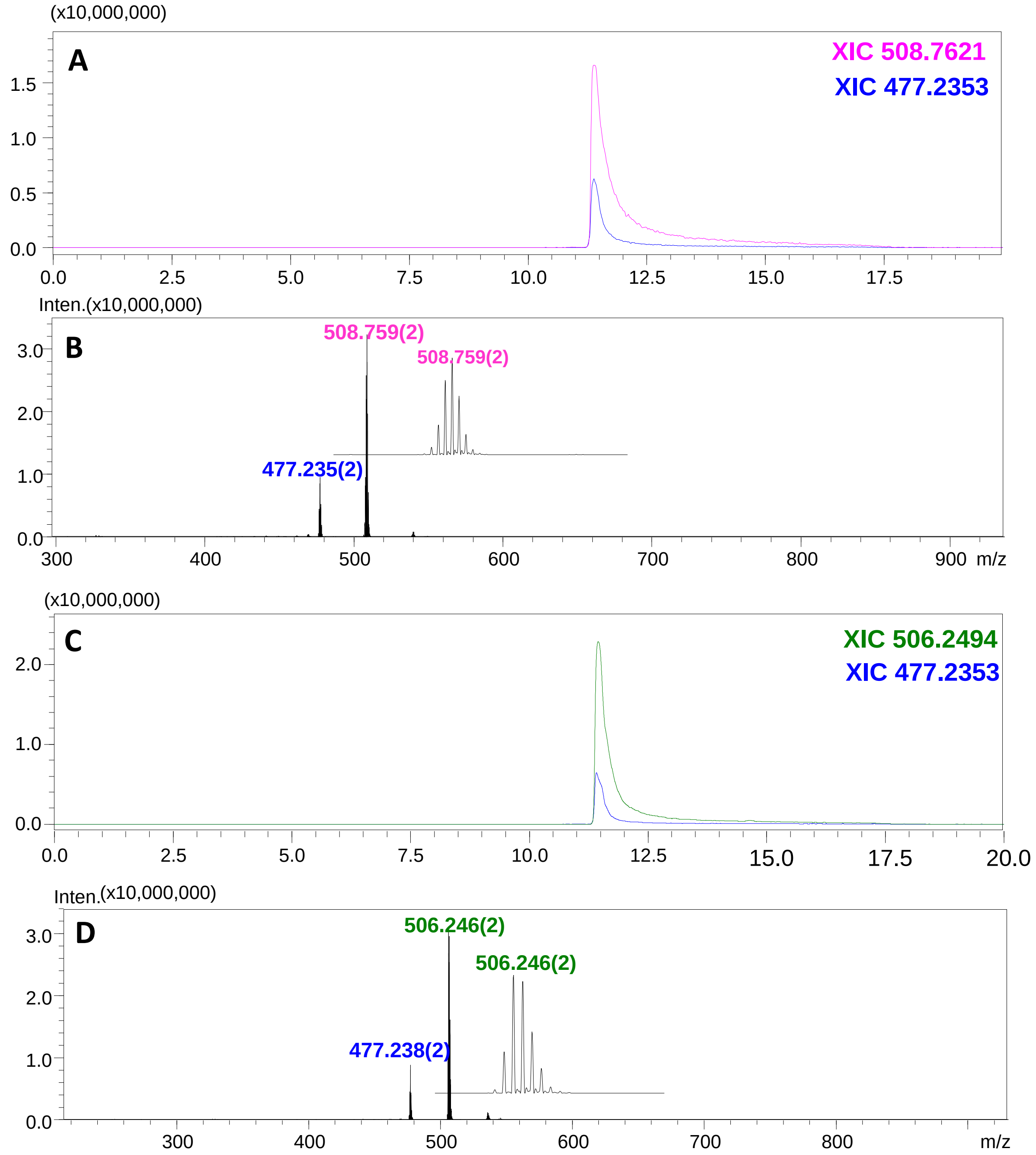

Figure S92 LC-MS analysis of incubation of pure **5** in acetone-d_6 for 4 days (MS value *m/z* = 508.759; *m/z* = 477.235) (A) and the same mixture diluted with water for 10 days (MS value *m/z* = 506.246; *m/z* = 477.235) (B); ESI-MS spectra (C, D);

4.25 LC-MS of products after reduction of 4a

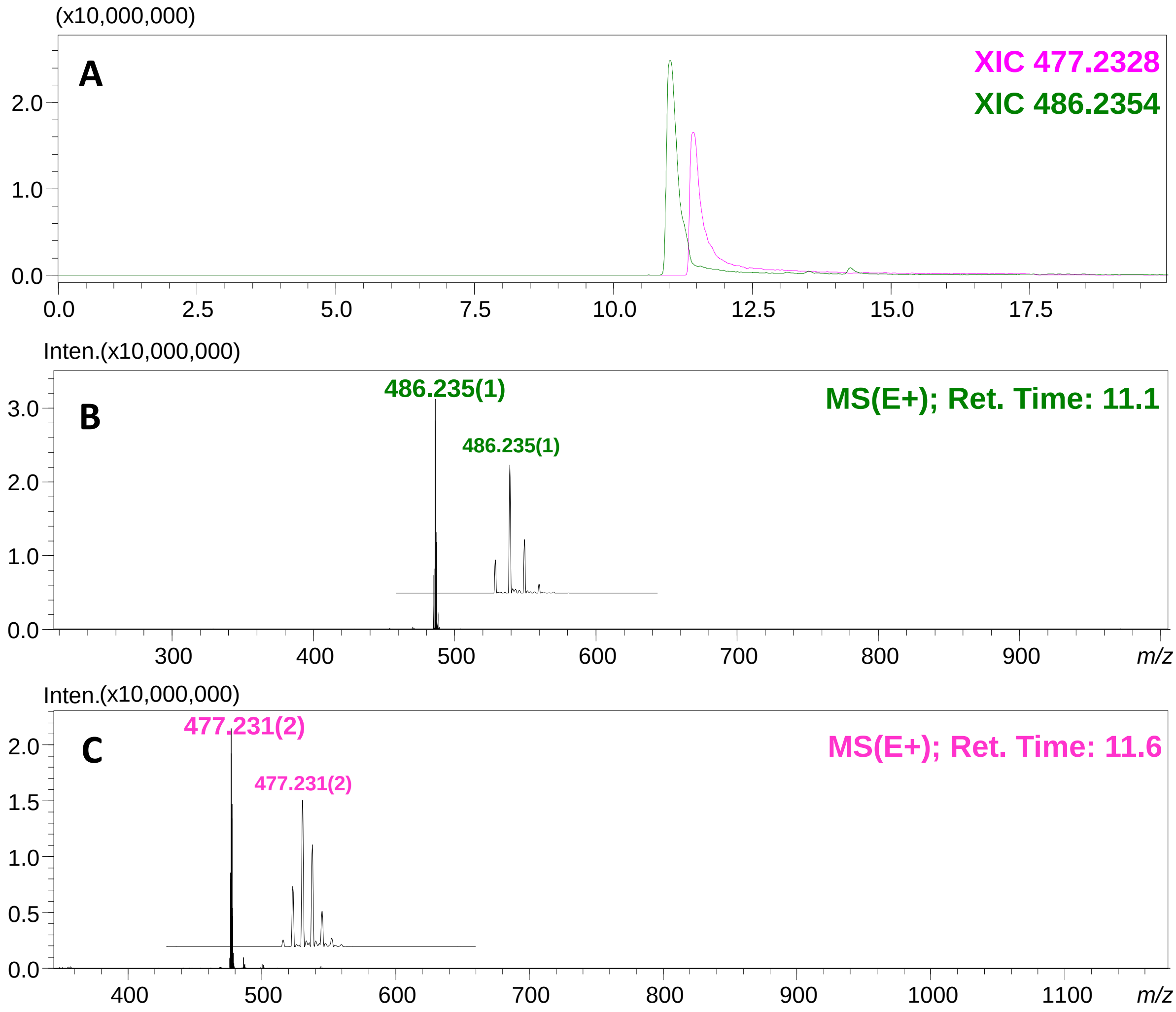

Figure S93 LC-MS analysis of mixture after reduction of **4a** using amalgamate (MS value *m/z* = 486.235; *m/z* = 477.231) (A) ; ESI-MS spectra (B,C).

4.26 LC-MS of products after incubation of 4a and 4b

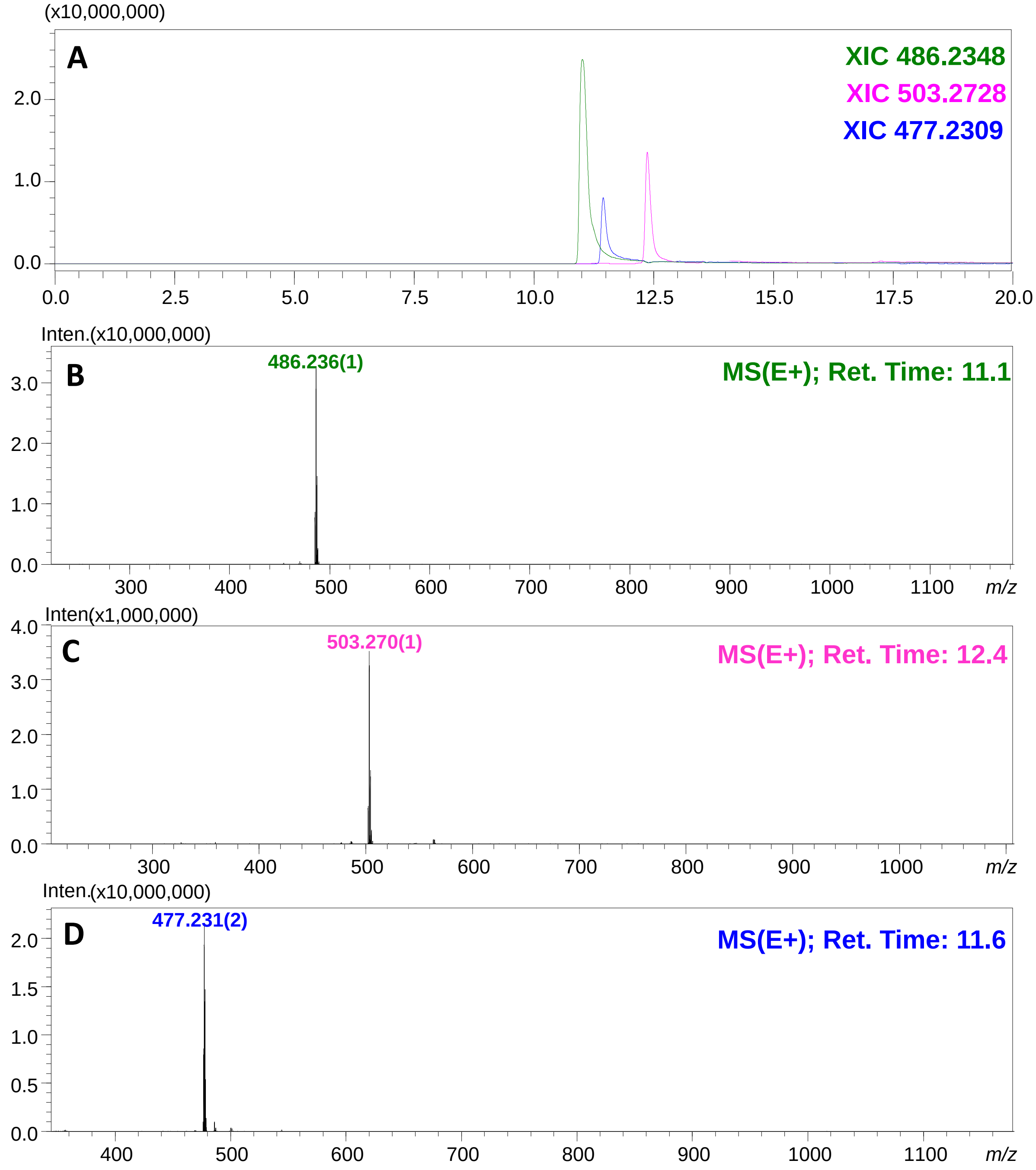

Figure S94 LC-MS analysis of mixture after incubation of **4a** and **4b** in $CDCl_3$ in 60°C for 12 h (MS value *m/z* = 486.236; *m/z* = 503.270; *m/z* = 477.231) (A) ; ESI-MS spectra (B-D).

4.27 LC-MS of crude products (Procedure 2a)

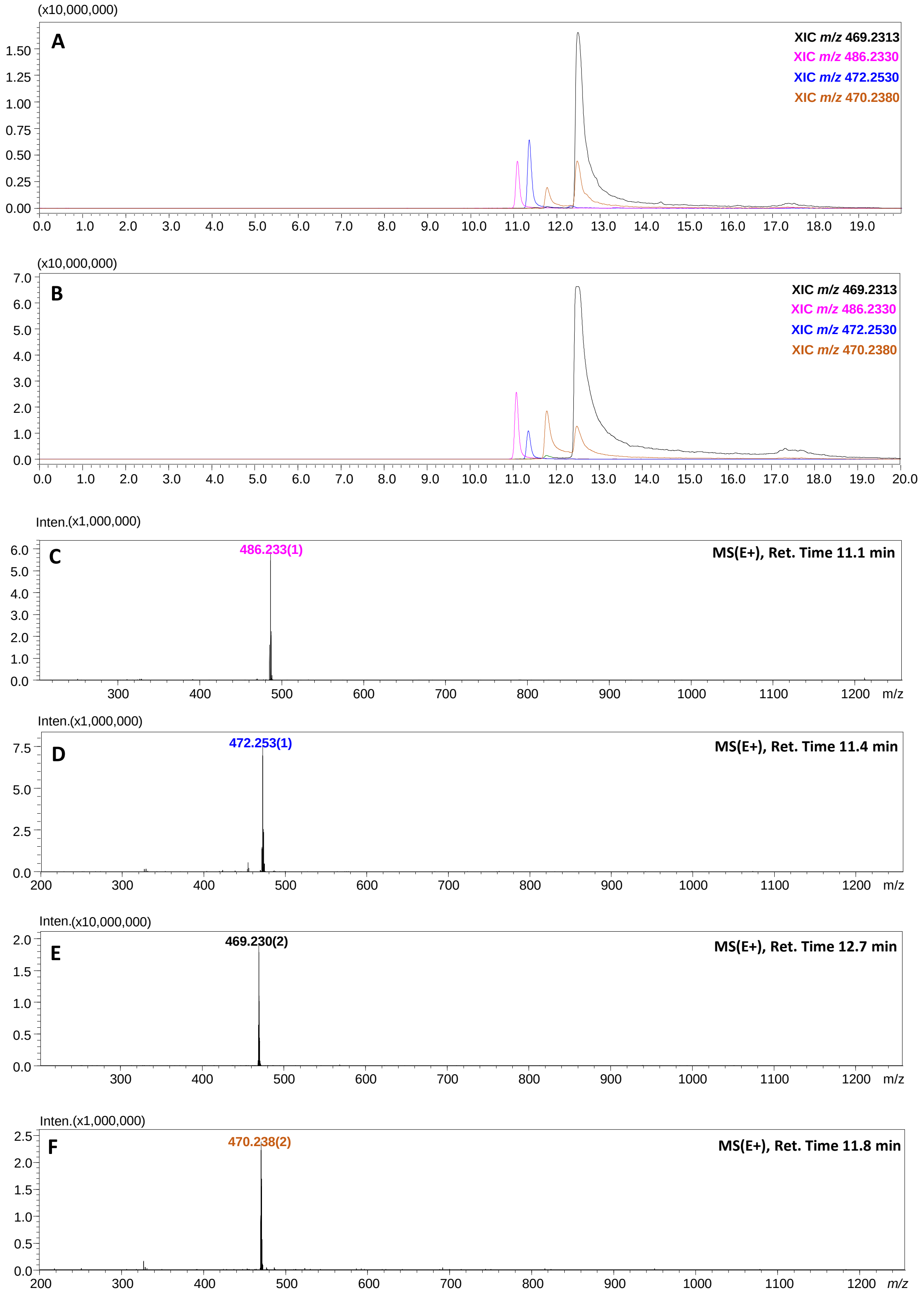

Figure S95 LC-MS analysis of crude mixture after the reaction with BBr_3 (MS value *m/z* = 486.236; *m/z* = 472.253; *m/z* = 469.230; *m/z* = 470.238) (A); after 5 days (B); ESI-MS spectra (C-F).

4.28 LC-MS of products before and after purification (Procedure 3)

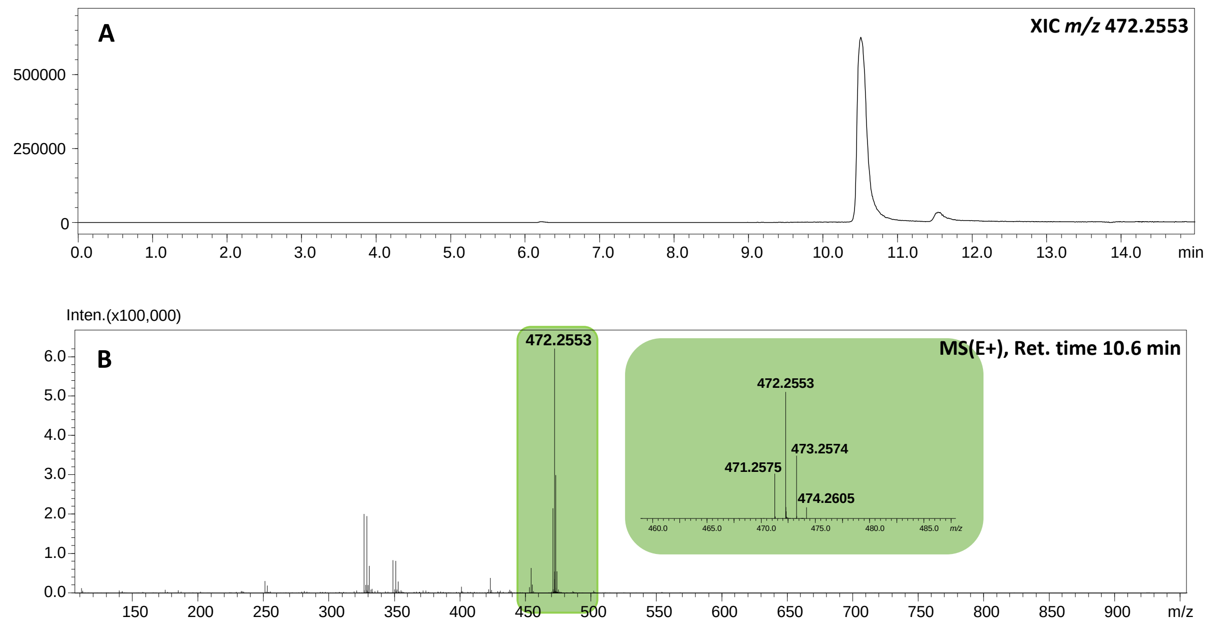

Figure S96 LC-MS analysis of crude product (MS value *m/z* = 472.2553) (A) ; ESI-MS spectra (B).

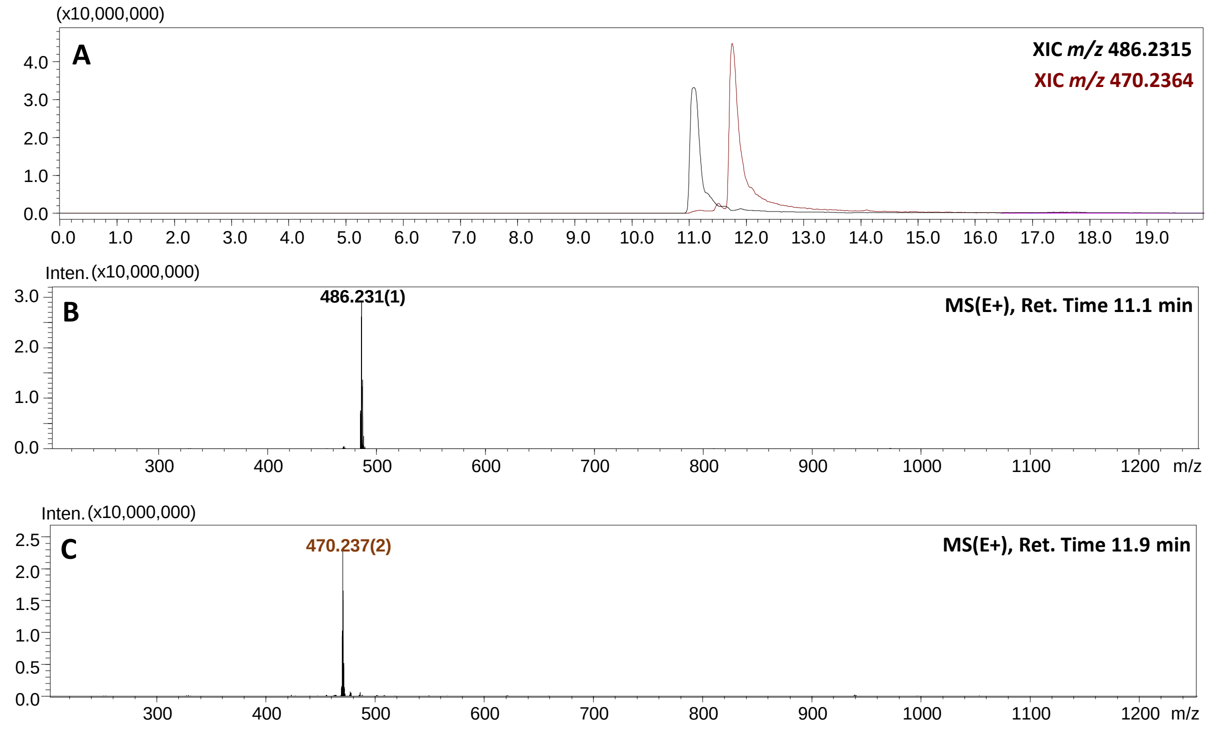

Figure S97 LC-MS analysis of products after purification (MS value *m/z* = 486.231, *m/z* = 470.237) (A) ; ESI-MS spectra (B,C).

5. UV-Vis spectra

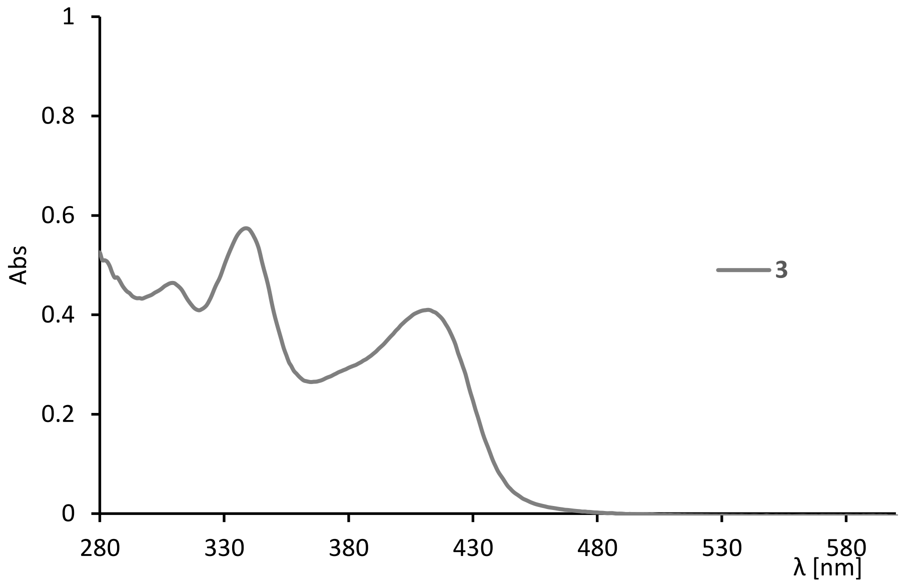

Figure S98. UV-Vis spectrum of **3** (DCM, 298K)

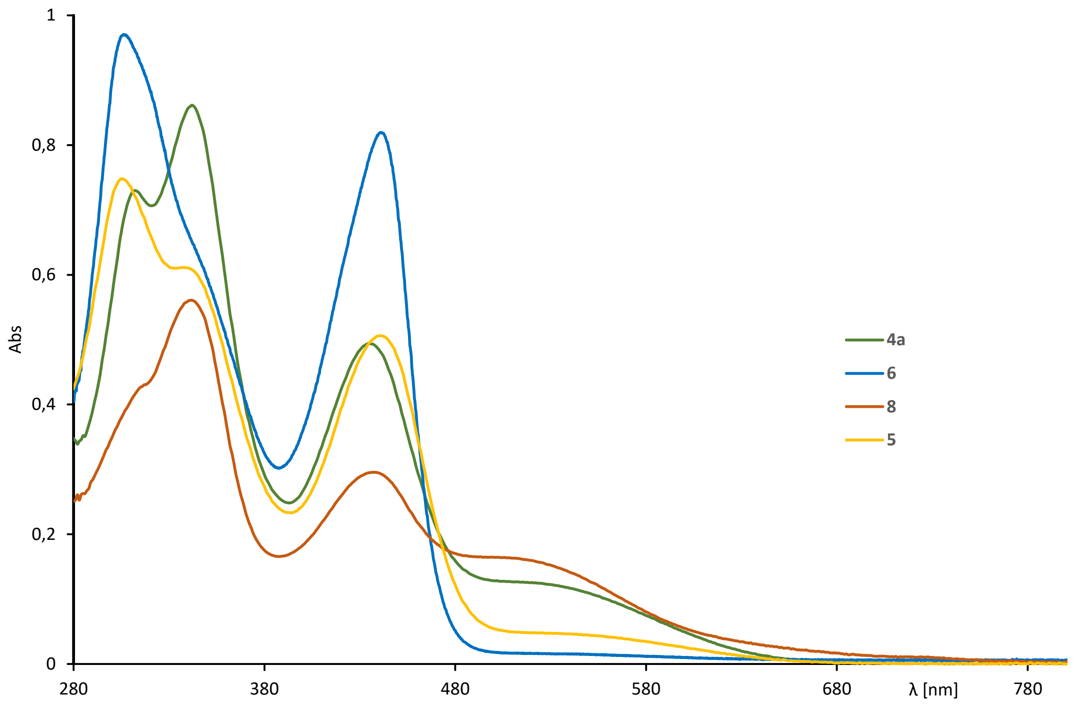

Figure S99. UV-Vis spectra (MeCN, 298K)

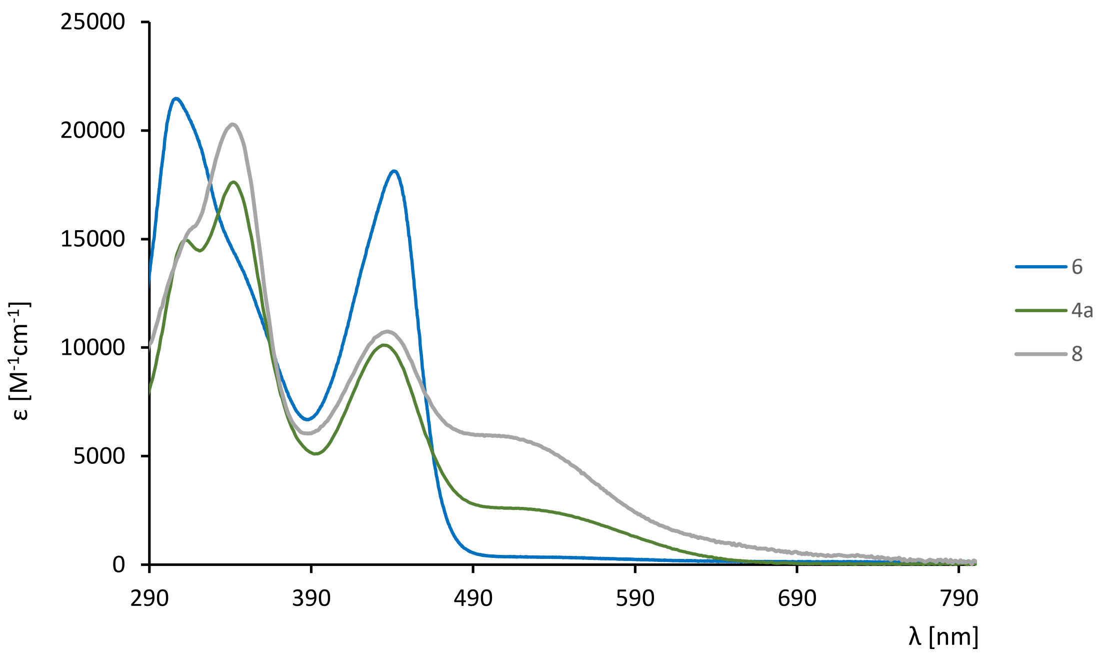

Figure S100. Extinction spectra (MeCN, 298K)

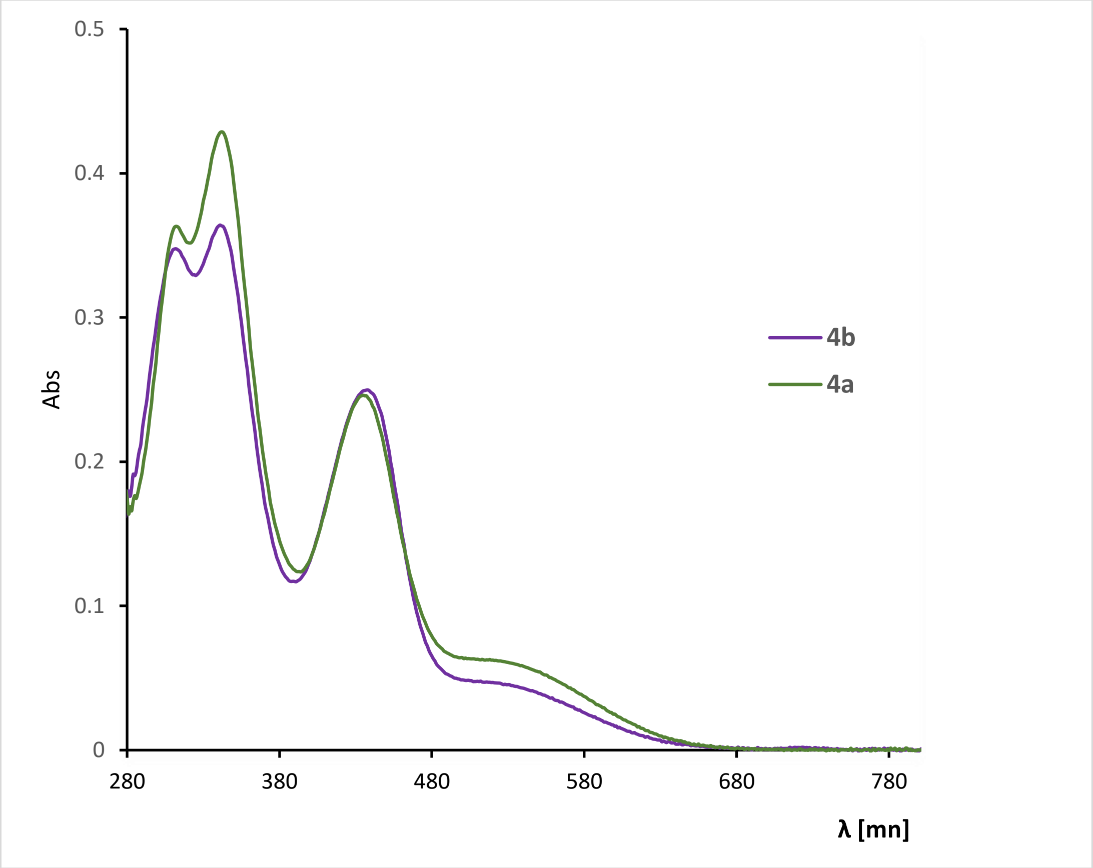

Figure S101. UV-Vis spectra of **4a** and **4b** (MeCN, 298K)

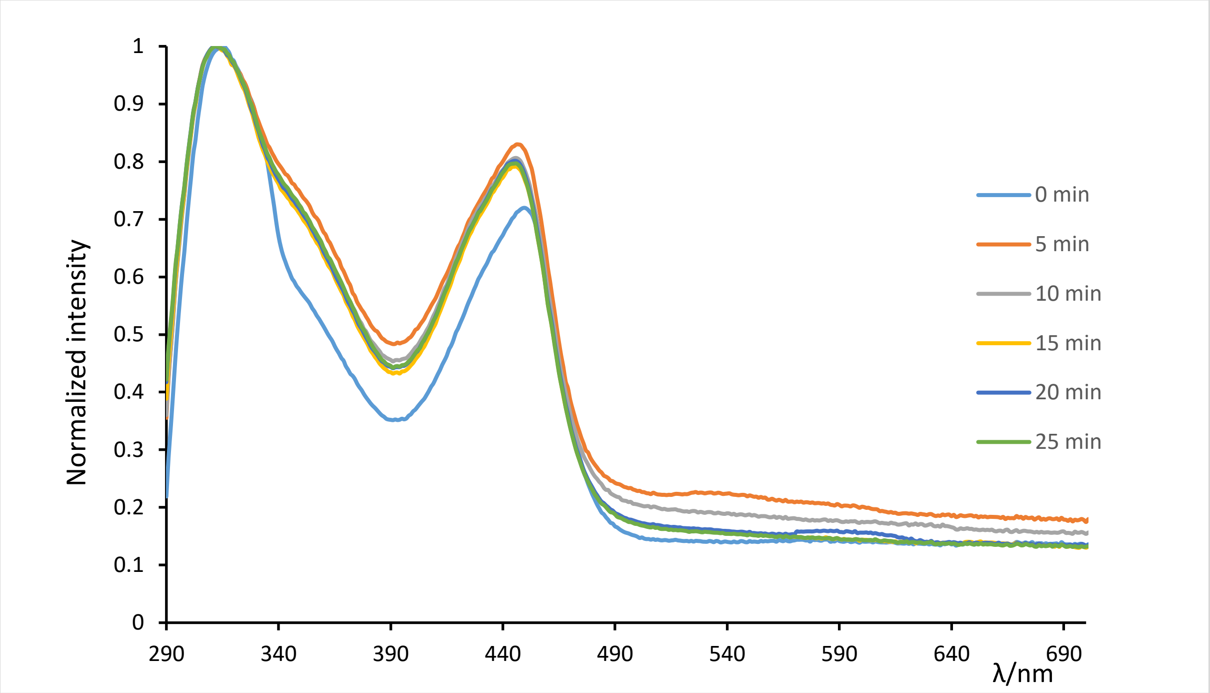

Figure S102. UV-VIS spectra of **6** under UV radiation (MeCN, 298K)

6. Emission spectra

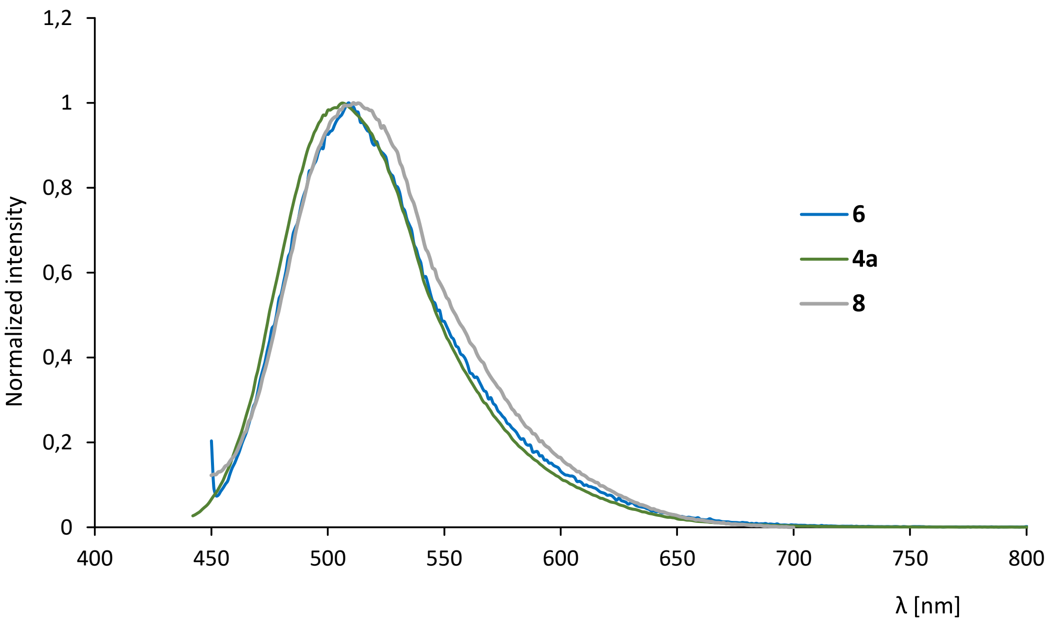

Figure S103. Emission spectra of **6** (blue), **4a** (green), **8** (grey) (MeCN, 298 K)

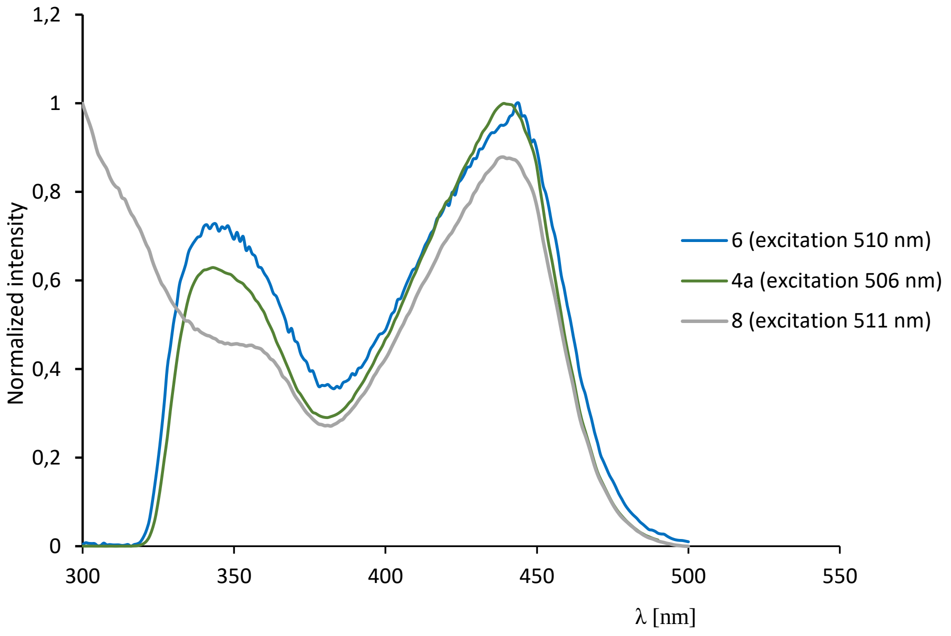

Figure S104. Excitation spectra of **6** (blue; emission detection 510 nm), **4a** (green; emission detection 506 nm) and 8 (grey; emission detection 511 nm) (MeCN, 298 K)

7. Crystal Data

Identification code	**3** kijba	**4a** mkij01a	**4a-Ac** mkij8pa	**6** mkijdibra	**8** mkij5a
CCDC number					
Empirical formula	$C_{31}H_{29}N_3O$•1.2 (CH_2Cl_2)•0.5(H_2O)	$[C_{31}H_{29}BN_3O_2]Cl$• 2$(CHCl_3)$	$[C_{34}H_{35}BN_3O_3]C_2F_3O_2$•$C_3H_6O$	$[C_{62}H_{56}B_2N_6O_2]Br_2$ •6$(CHCl_3)$	$[C_{62}H_{58}B_2N_6O_2](C_2F_3O_2)_2$ •2.75(C_3H_6O)•(H_2O)
Formula weight	570.49	760.57	715.56	1814.77	1344.53
Temperature/K	100	100	80	100	100
Crystal system	monoclinic	monoclinic	triclinic	monoclinic	triclinic
Space group	$P2_1/c$	$P2_1/c$	P-1	$C2/c$	P-1
a/Å	16.088(5)	15.180(8)	15.015(3)	23.9735(6)	12.012(3)
b/Å	15.757(4)	14.412(8)	16.747(3)	13.8399(3)	13.164(2)
c/Å	11.772(3)	15.751(9)	16.884(3)	24.1518(9)	21.540(4)
α/°			117.50(3)		77.28(2)
β/°	109.16(3)	102.04(4)	106.23(3)	104.620(3)	89.74(3)
γ/°			91.96(2)		86.42(3)
Volume/Å^3	2818.9(14)	3370(3)	3548.7(15)	7753.9(4)	3315.8(12)
Temperature/K	100(2)	100(2)	80(2)	100(2)	100(2)
Z	4	4	4	4	2
ρ_{calc}g/cm^3	1.344	1.499	1.339	1.555	1.347
μ/mm^{-1}	0.30	5.68	0.84	7.42	0.83
F(000)	1198	1560	1504	3656	1412
Crystal size/mm	0.45×0.31×0.12	0.07×0.06×0.03	0.36×0.22×0.05	0.16×0.03×0.02	0.10×0.05×0.01
Radiation type	MoK$_\alpha$(λ = 0.71073)	CuK$_\alpha$(λ = 1.54184)	CuK$_\alpha$(λ = 1.54184)	CuK$_\alpha$(λ = 1.54184)	CuK$_\alpha$(λ = 1.54184)
2Θ range for data collection/°	2.9 to 27.5	4.2 to 68.0	3.0 to 75.8	3.7 to 68.0	2.1 to 67.5
Index ranges	−20 ≤ h ≤ 20 −20 ≤ k ≤ 19 −15 ≤ l ≤ 15	−14 ≤ h ≤ 18 −17 ≤ k ≤ 17 −18 ≤ l ≤ 18	−18 ≤ h ≤ 17 −20 ≤ k ≤ 20 −20 ≤ l ≤ 21	−28 ≤ h ≤ 28 −16 ≤ k ≤ 16 −28 ≤ l ≤ 28	−14 ≤ h ≤ 14 −13 ≤ k ≤ 15 −25 ≤ l ≤ 25
Reflections collected	22853	14997	68131	12449	55770
Independent reflections	6415	6014	14546	12449	11842
R_{int}	0.0672	0.0851	0.0277		0.0482
Final R_1 values (I > 2σ(I))	0.0676	0.0779	0.0444	0.0504	0.0821
Final wR(F^2) values (I > 2σ(I))	0.1649	0.1683	0.1219	0.1045	0.2231
Final R_1 values (all data)	0.0965	0.1169	0.0491	0.0967	0.0956
Final wR(F^2) values (all data)	0.1901	0.1889	0.1256	0.1155	0.2331
Goodness of fit on F^2	1.038	1.062	1.071	0.818	1.067
Largest diff. peak/hole /eÅ^{-3}	0.37, −0.55	0.48, −0.49	0.56, −0.38	0.74, −0.65	0.87, −0.53

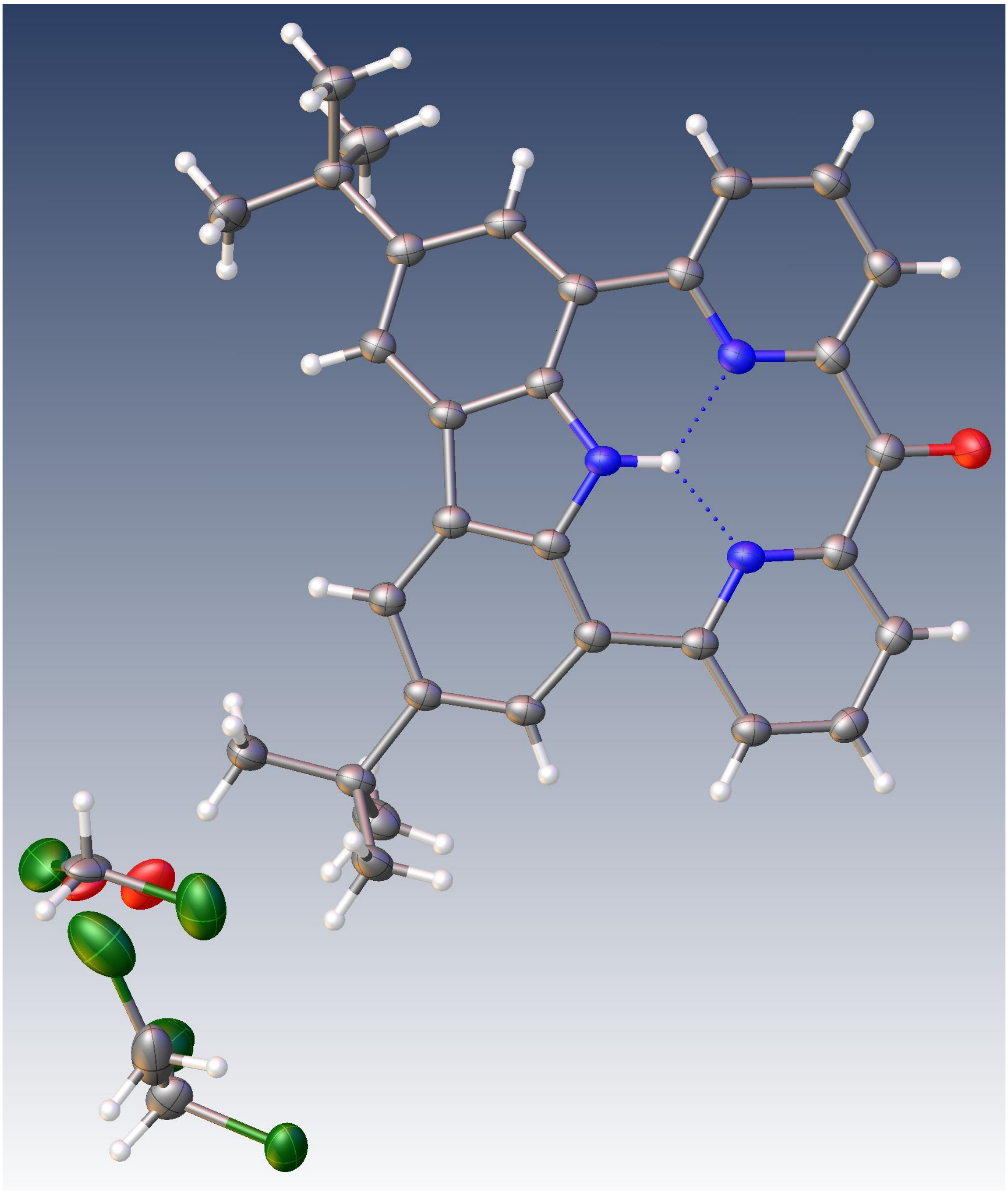

Figure S105. Crystal structure of **3**. Thermal ellipsoids present 50% probability

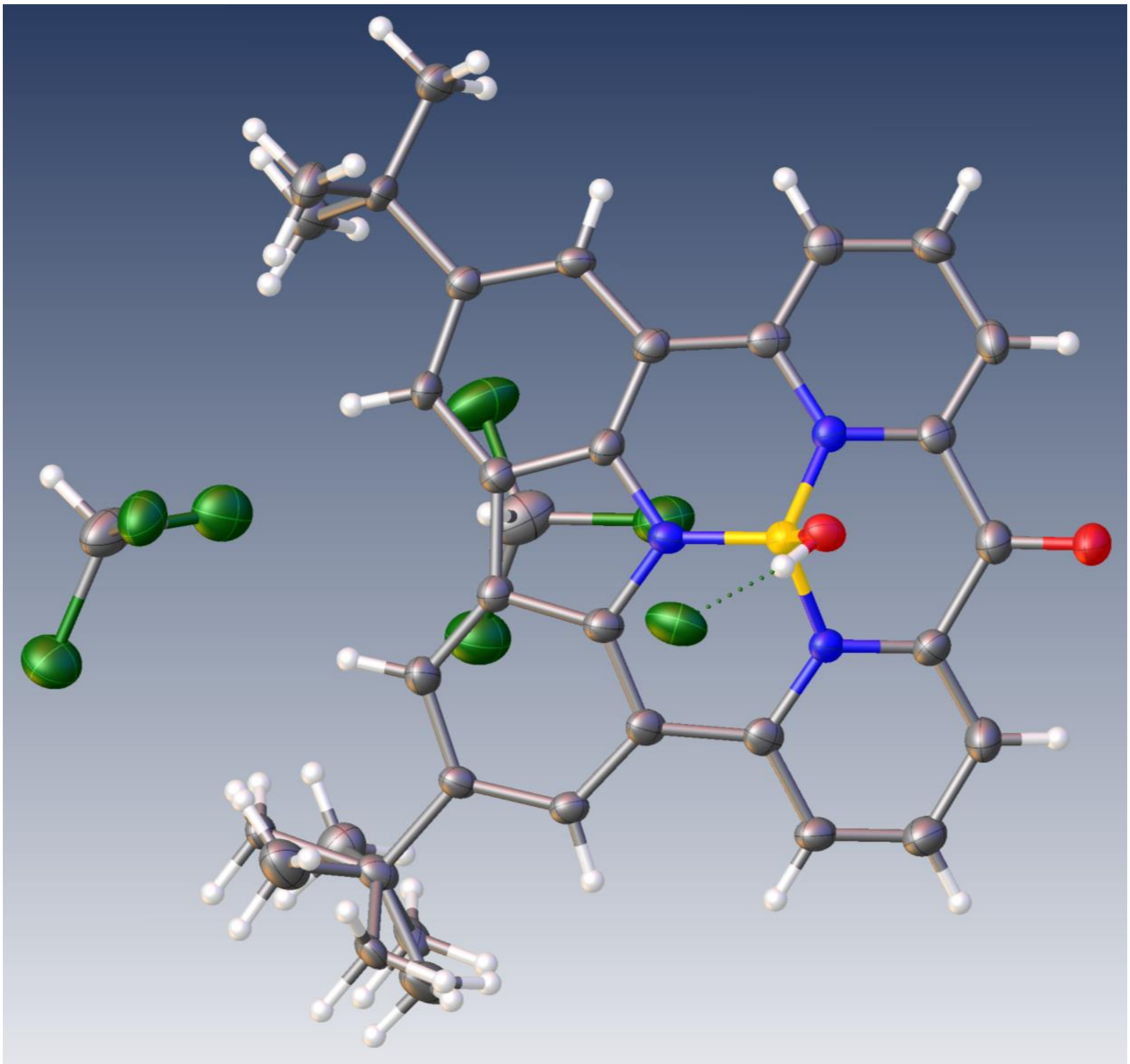

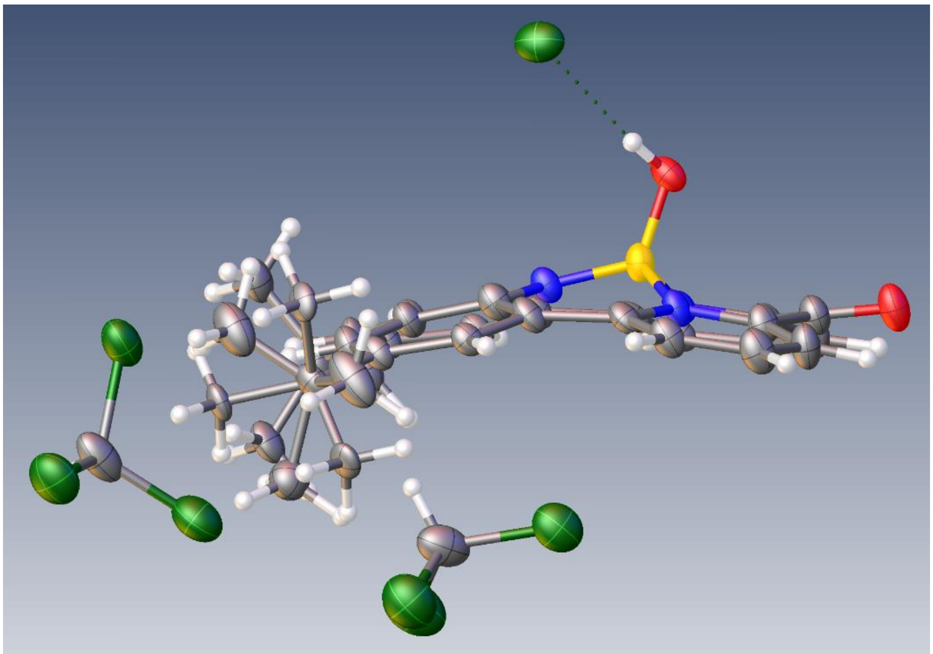

Figure S106. Crystal structure of **4a**. Thermal ellipsoids present 50% probability.

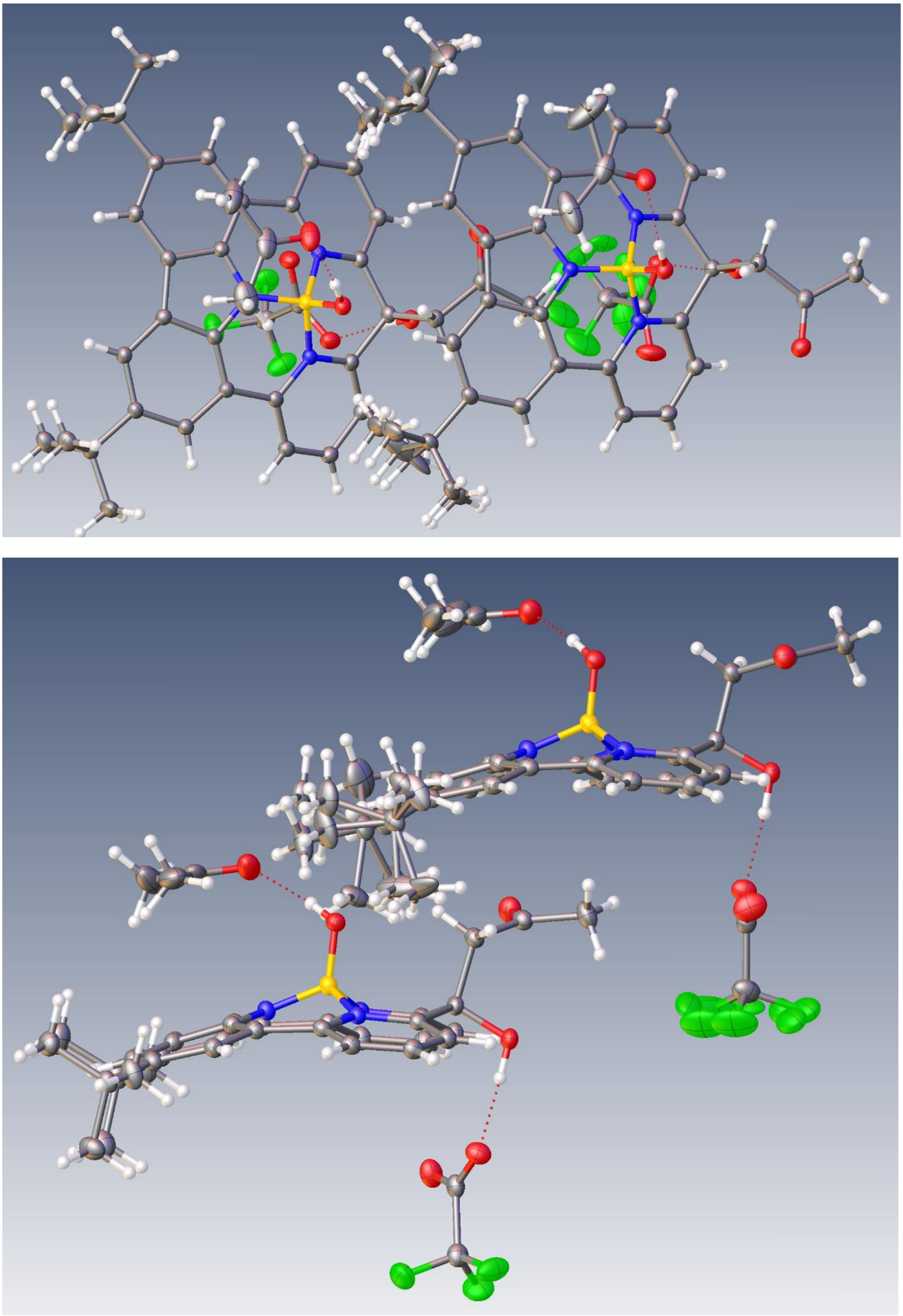

Figure S107. Crystal structure of **4a**_*Ac*. Thermal ellipsoids present 50% probability.

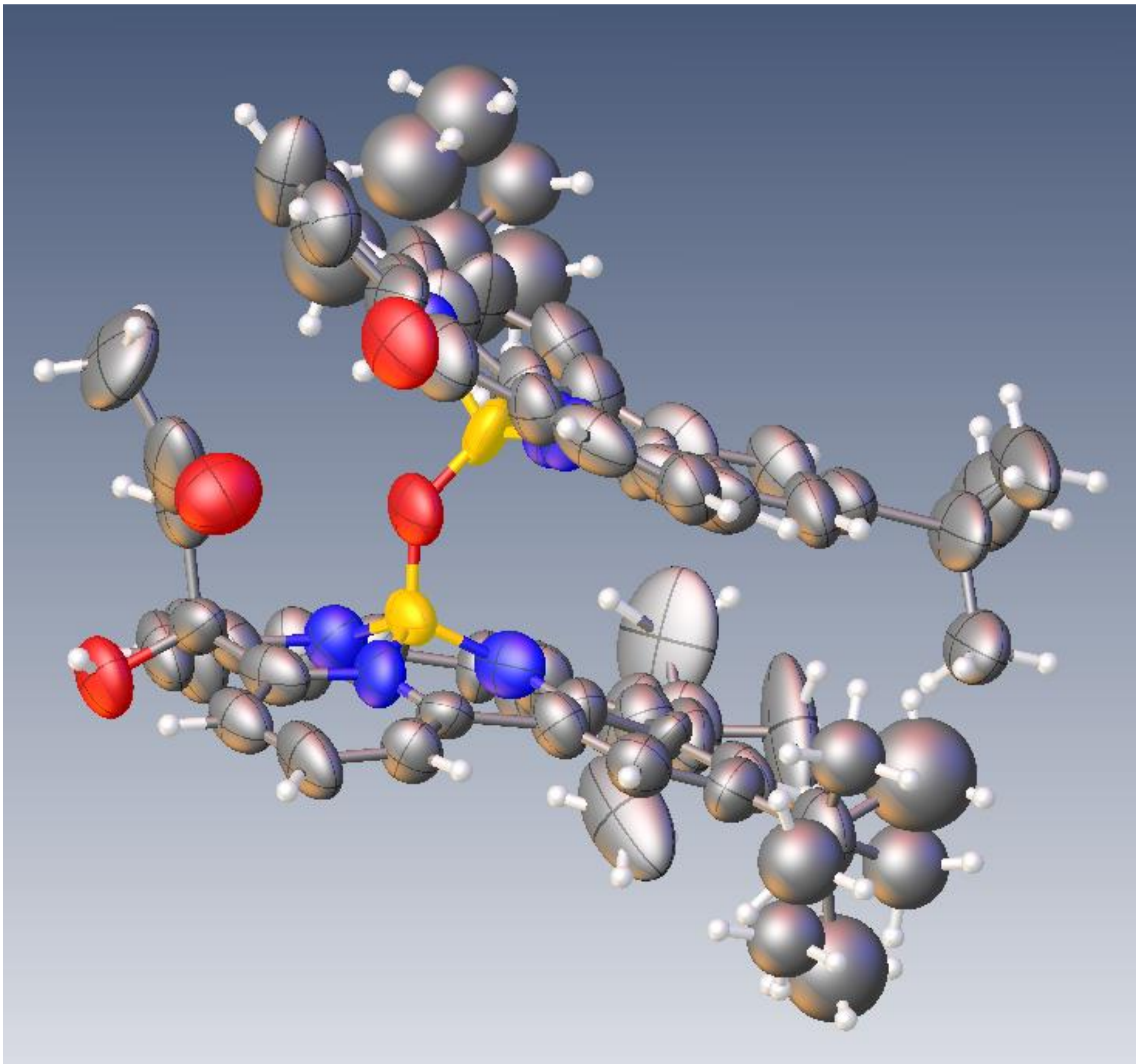

Figure S108. Model of **5-Ac.** Thermal ellipsoids present 50% probability

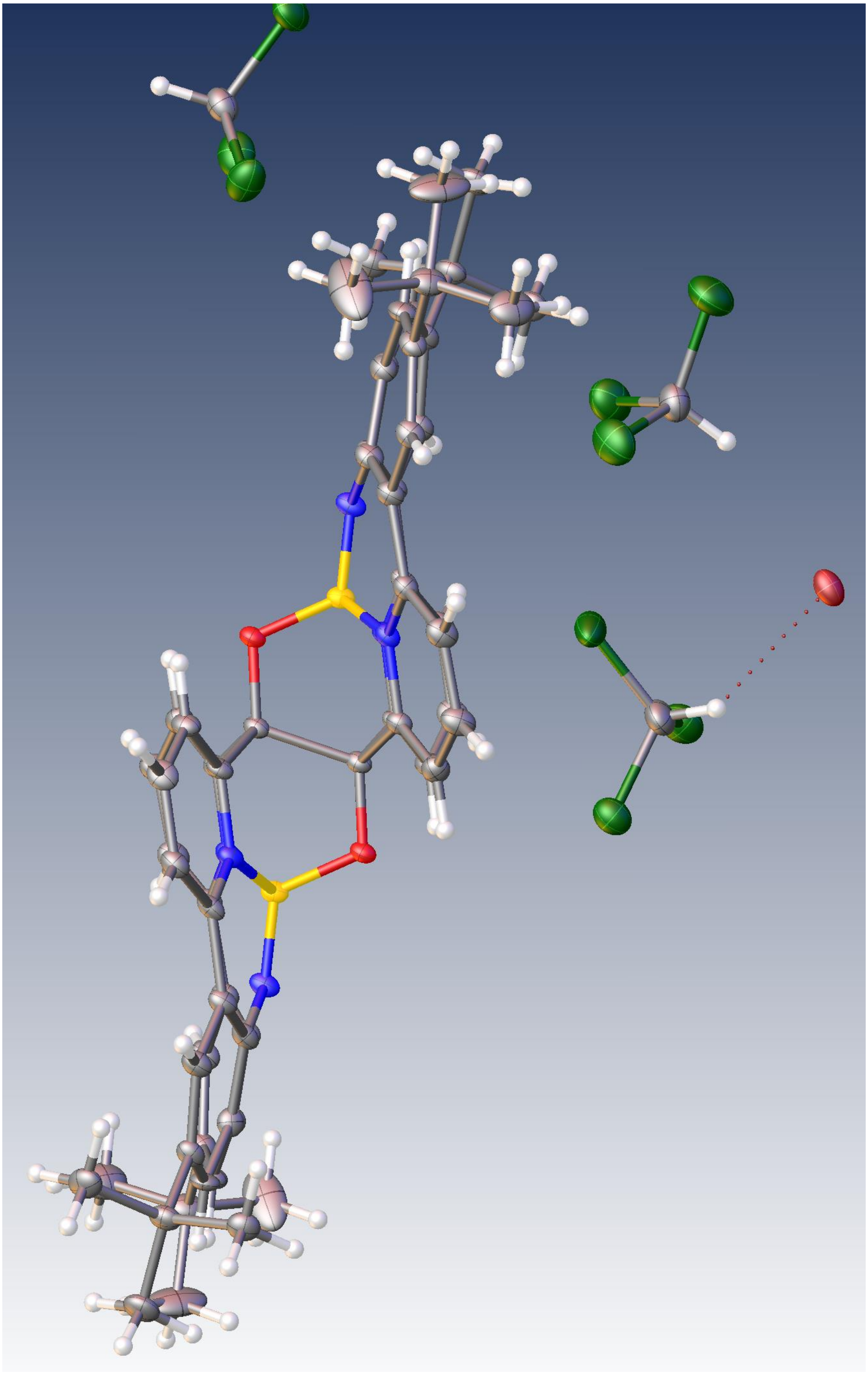

Figure S109. Crystal structure of **6**. Thermal ellipsoids present 50% probability

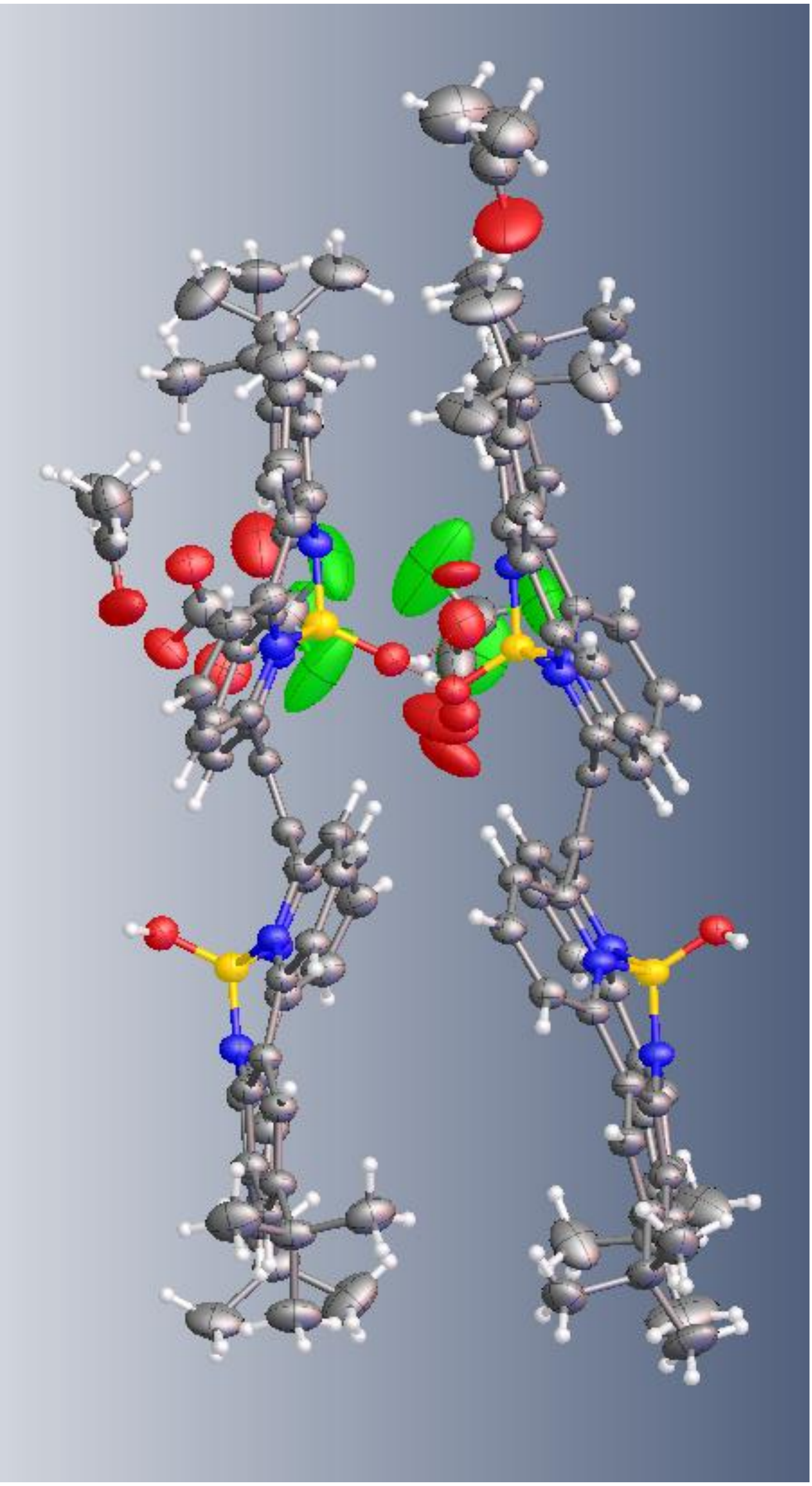

Figure S110. Crystal structure of **8**. Thermal ellipsoids show 50% probability.
